# Balancing Bulkiness in Gold(I) Phosphino‐triazole Catalysis

**DOI:** 10.1002/ejoc.201900850

**Published:** 2019-07-30

**Authors:** Yiming Zhao, Matthew G. Wakeling, Fernanda Meloni, Tze Jing Sum, Huy van Nguyen, Benjamin R. Buckley, Paul W. Davies, John S. Fossey

**Affiliations:** ^1^ School of Chemistry University of Birmingham Edgbaston B15 2TT Birmingham West Midlands UK; ^2^ Department of Chemistry Loughborough University LE11 3TU Loughborough Leicestershire UK

**Keywords:** Click chemistry, Gold, Homogeneous catalysis, Phosphane ligands, Triazoles

## Abstract

The syntheses of a series of 1‐phenyl‐5‐phosphino 1,2,3‐triazoles are disclosed, within which, the phosphorus atom (at the 5‐position of a triazole) is appended by one, two or three triazole motifs, and the valency of the phosphorus(III) atom is completed by two, one or zero ancillary (phenyl or cyclohexyl) groups respectively. This series of phosphines was compared with tricyclohexylphosphine and triphenylphosphine to study the effect of increasing the number of triazoles appended to the central phosphorus atom from zero to three triazoles. Gold(I) chloride complexes of the synthesised ligands were prepared and analysed by techniques including single‐crystal X‐ray diffraction structure determination. Gold(I) complexes were also prepared from 1‐(2,6‐dimethoxy)‐phenyl‐5‐dicyclohexyl‐phosphino 1,2,3‐triazole and 1‐(2,6‐dimethoxy)‐phenyl‐5‐diphenyl‐phosphino 1,2,3‐triazole ligands. The crystal structures thus obtained were examined using the *SambVca (2.0)* web tool and percentage buried volumes determined. The effectiveness of these gold(I) chloride complexes to serve as precatalysts for alkyne hydration were assessed. Furthermore, the regioselectivity of hydration of but‐1‐yne‐1,4‐diyldibenzene was probed.

## Introduction

Bulky phosphines offer significant and well‐documented advantages as ligands in metal‐catalysed reactions. The landmark contributions of Buchwald and co‐workers have led to a deeper understanding of the synthetic chemistry enabled by such bulky ligands, and ligands such as S‐Phos and X‐Phos are an often‐required component of the toolbox of today's synthetic chemists.^1^ There is emerging interest in the importance of categorising and evaluating steric and electronic parameters^2^ of these types of bulky phosphines^3^ in order to correlate and ultimately predict their suitability for use in metal‐mediated catalysis.^4^ The Tolman cone angle has been used to describe the bulkiness of ligands,^5^ this descriptor has been complemented by Nolan describing bulkiness in terms of a percentage buried volume (%*V*_bur_).^6^ The %*V*_bur_ described by a ligand can be calculated by using Cavallo and co‐workers' web tool *SambVca (2.0)*.^7^

**Figure 1:**
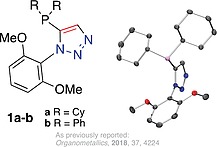
Selected examples of a previously reported 1‐aryl‐5‐phosphino triazoles (left) (**1a**–**b**) and ***single‐crystal X‐ray*** diffraction structure of **1a** (right), as reported elsewhere, H‐atoms omitted for clarity.^8^

Some authors of this report previously detailed the synthesis, and application to palladium‐catalysed Suzuki–Miyaura cross‐coupling reactions, of 1,2,3‐triazole‐containing phosphines including analogues of the aforementioned Buchwald‐type ligands, such as **1a** (Figure 1).^8^ Analysis of the steric parameters (buried volume)^6^, ^9^ using the *SambVca (2.0)* web tool,^7^ confirmed that the bulkier ligands of those tested were the most effective in said catalysis.^8^

**Figure 2:**
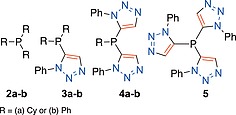
The series of phosphines conceived to probe the influence the number of 1‐phenyl‐5‐phosphino triazole fragments about the phosphorus centre upon their “bulkiness” as ligands.

The 1‐aryl‐5‐phosphino‐triazole ligands, such as **1a**, present their 1‐aryl fragment in the same orientation as the phosphorus lone pair (of the free ligand) or in the direction of the metal (of a phosphorus‐metal complex thereof), thus significantly impacting the determined buried volume.^8^ Noting that *ortho*‐aryl tris‐phenylene phosphines (first reported, and somewhat overlooked, in 1940)^10^ can impart favourable properties as ligands in catalysis,^11^ it struck us that a bulky, and thus possibly superior, version of the triazole phosphine ligand is possible if the phosphorus atom were to be flanked by up to three, rather than one, triazole. A series of ligands ranging from zero to three triazoles, for comparison of the manifested bulk about the phosphorus centre by 1,5‐disubstituted‐triazole motif(s), was proposed. As such, a series of phosphines comprising of triphenylphosphine (**2a**) and tricyclohexylphosphine (**2b**) along with mono‐triazole‐appended phosphines **3a** and **3b** (available from a previous study), and the previously unprepared bis‐triazoles **4a** and **4b** and 1‐phenyl‐5‐phosphino‐tris‐triazole, **5** (Figure 2), was conceived.

## Results and Discussion

The substituents attached to phosphorus and the triazole nitrogen, could in principle be varied significantly. To provide a benchmark in this initial investigation cyclohexyl (**a**) and phenyl (**b**) groups attached to phosphorus were selected and the substituent attached at the 1‐*N*‐position of the triazole was restricted to phenyl in the first instance (Figure 2). As such two series of aryl‐ and alkyl‐substituted phosphines were planned.

### Phosphine Synthesis

**Scheme 1:**
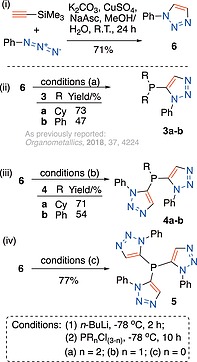
(i) The synthesis of triazole **6**. Deprotonation of **6** and reaction with: (ii) dicyclohexyl‐ or diphenyl‐phosphorus chloride depicting the previously reported synthesis of **3a** and **3b**; (iii) cyclohexyl‐ or phenyl‐phosphorus dichloride for the synthesis of **4a** and **4b** by respectively; (iv) phosphorus trichloride for the synthesis of tristriazole phosphine **5**.

Dicylohexylphosphino‐ and 5‐diphenylphosphino‐ 1‐phenyl triazoles, **3a** and **3b** respectively, were available from an earlier study. Their synthesis required **6** [Scheme 1 (i)] to be deprotonated with *n*‐butyllithium^12^ and reacted with dicyclohexyl‐ or diphenyl‐ phosphorus chloride, Scheme 1 (ii) (see earlier reports, and citations therein^8^, ^13^).

The same protocol for selective deprotonation of **6** at the 5‐position,^12^ was followed by addition of cyclohexyl‐ or phenyl‐ phosphorus dichloride leading to the formation and subsequent isolation of **4a** and **4b** in 71% and 54% yield respectively [Scheme 1 (iii)]. When a third of an equivalent of phosphorus trichloride was added to deprotonated **6**, the expected tris‐1‐phenyl 5‐phosphino triazole **5**, resulted and was subsequently isolated in 77% yield [Scheme 1 (iv)]. The proton and carbon NMR spectrums of **5** (in [D]chloroform at ambient temperature) displayed a single set of well‐defined resonances, corresponding to the three equivalent triazole arms of the **5**, suggesting a high degree of symmetry in solution on the NMR timescale.

**Figure 3:**
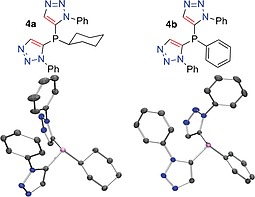
(a) A molecule of **4a** determined by single‐crystal X‐ray diffraction structure analysis. *Ortep* representation, ellipsoid probability 50% (rendered in *PovRay*, H‐atoms omitted for clarity) (lower) and a chemical drawing of **4a** (upper). (b) A molecule of **4b** determined by single‐crystal X‐ray diffraction structure analysis. *Ortep* representation, ellipsoid probability 50% (rendered in *PovRay*, H‐atoms omitted for clarity) (lower) and a chemical drawing of **4b** (upper).

**Figure 4:**
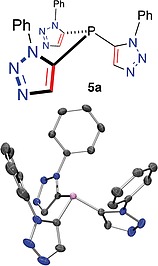
A molecule of **5** determined by single‐crystal X‐ray diffraction structure analysis. *Ortep* representation, ellipsoid probability 50% (rendered in *PovRay*, H‐atoms omitted for clarity) (lower) and a chemical drawing of **5** in approximately the same orientation (upper).

Both **4a** and **4b** provided single crystals suitable for structural analysis by XRD (Figure 3a and Figure 3b, respectively, and supplementary material). In both cases the 1‐phenyl substituents of the triazole components point broadly in the same general direction as the phosphorus lone pair, thus boding well for a systematic study of the effect of modulating the steric crowding or bulkiness about a coordinated metal in a catalytically relevant complex. For compound **5**, a single crystal XRD structure was obtained (Figure 4), the solid‐state conformation contains two unique molecules in the unit cell, both of which show the aryl arms of the triazole are directed along the same orientation as the phosphorus lone pair, creating a bowl‐shaped ligand.

### Gold(I) Chloride Complex Synthesis and Structural Analysis

**Scheme 2:**
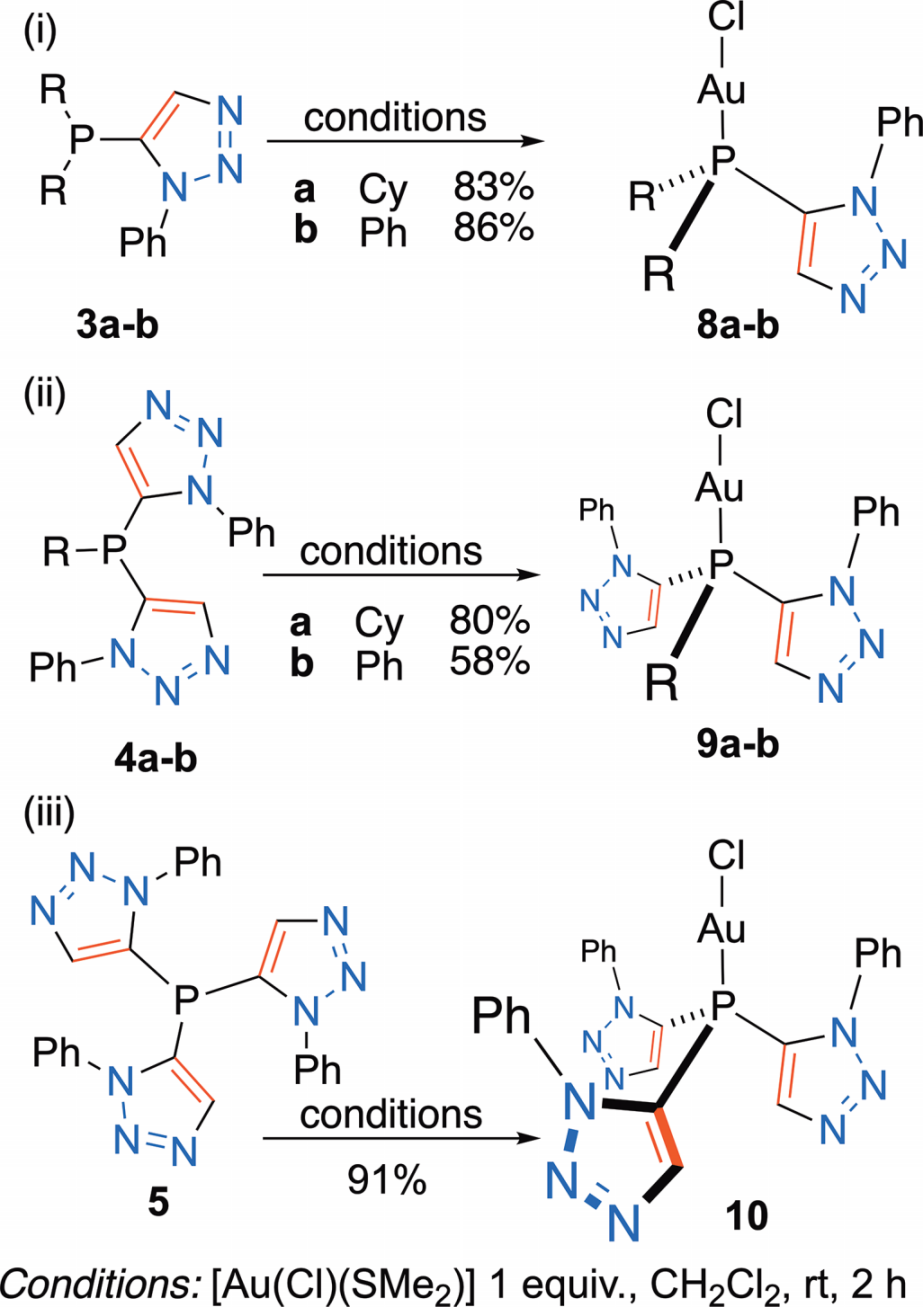
Synthesis of gold(I) chloride complexes (i) **8a**–**b**; (ii) **9a**–**b**; and (iii) **10**.

Complemented by commercially sourced tricyclohexylphosphine **2a** and triphenylphosphine **2b**, two ligand sets corresponding to Figure 2 (R = Cy or Ph) were therefore available for comparison in the complexation of a chosen metal. Owing to the importance of gold(I) phosphines in catalysis,^14^ and that X‐ray crystal structures of gold(I) chloride complexes **2a** and **2b** have been previously reported by others,^15^ the gold(I) chloride complexes of the ligands in the series presented in Figure 2 were compared. Ligation of the triazole‐phosphines (**3–5**) to gold(I), through phosphorus, was achieved by performing dimethyl sulfide‐phosphine exchange reactions on chloro(dimethyl sulfide)gold(I) (Scheme 2). Isolated yields of the corresponding gold(I) chloride complexes ranged from 58 to 91%. Triphenylphosphine and tricyclohexylphosphine gold(I) chloride complexes (**7a** and **7b**) were purchased from commercial suppliers.

**Figure 5:**
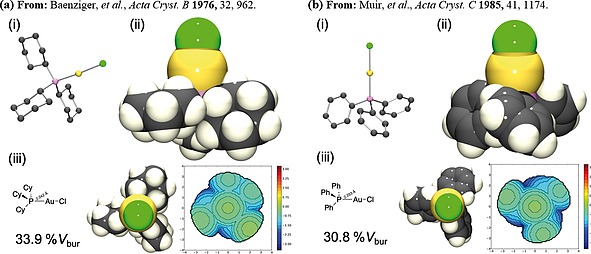
(a) Single‐crystal X‐ray diffraction structure of arising from literature deposited CIF file of **7a**: (i) *Ortep* representation, ellipsoid probability 50% (rendered in *PovRay*, H‐atoms omitted for clarity); (ii) Space‐filling representation. (iii) Percentage buried volume determined from the crystal structure of **7a** (33.9%) steric map of ligand depicted (right).[15a] (b) Single‐crystal X‐ray diffraction structure of **7b** arising from literature deposited CIF file: (i) *Ortep* representation, ellipsoid probability 50% (rendered in *PovRay*, H‐atoms omitted for clarity). (ii) Space‐filling representation. (iii) Percentage buried volume determined from the crystal structure of **7b** (30.8%) steric map of ligand depicted (right).[15b]

**Figure 6:**
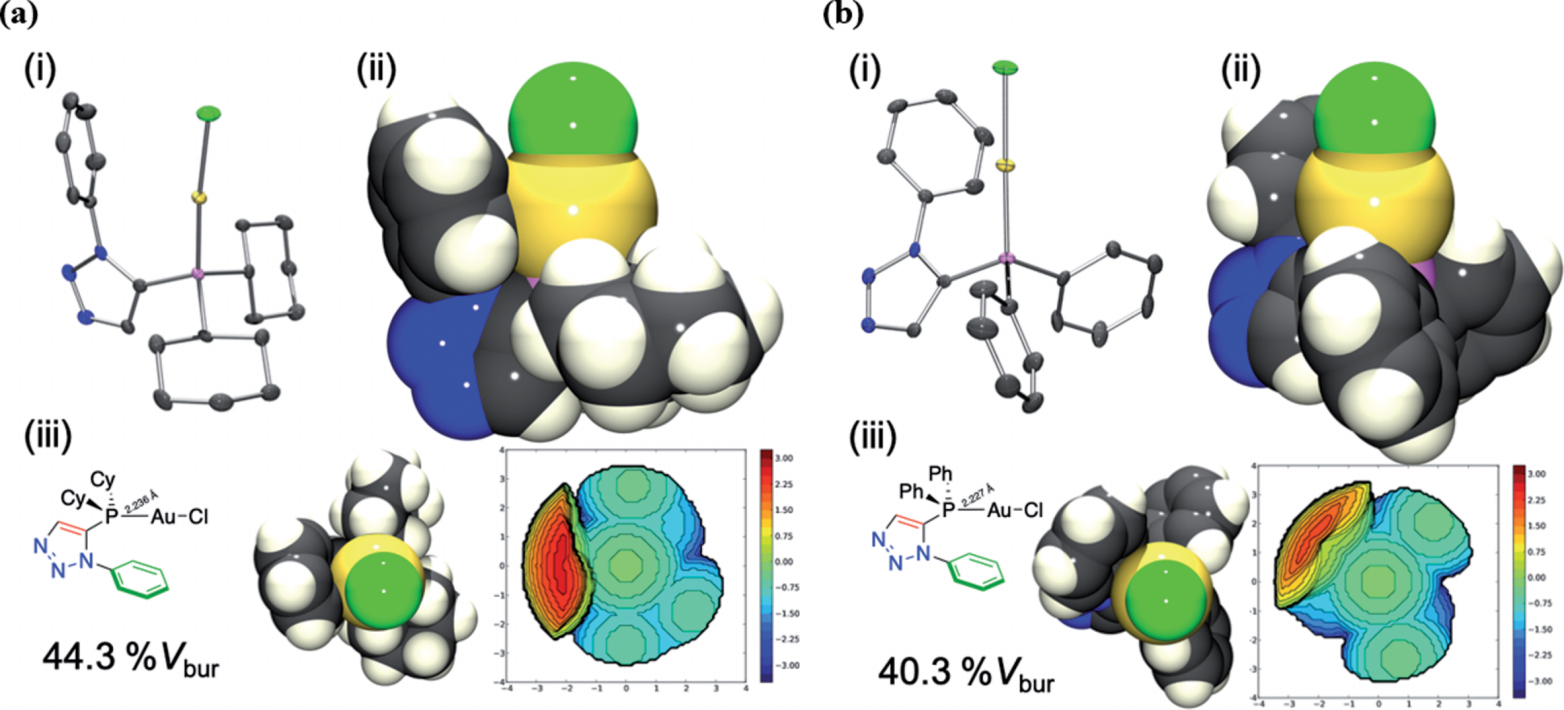
(a) Single‐crystal X‐ray diffraction structure of **8a**: (i) *Ortep* representation, ellipsoid probability 50% (rendered in *PovRay*, H‐atoms omitted for clarity). (ii) Space‐filling representation. (iii) Percentage buried volume determined from the crystal structure of **8a** (44.3%) steric map of ligand depicted (right). (b) Single‐crystal X‐ray diffraction structure of **8b**: (i) *Ortep* representation, ellipsoid probability 50% (rendered in *PovRay*, H‐atoms omitted for clarity). (ii) Space filing representation. (iii) Percentage buried volume determined from the crystal structure of **8b** (40.3%) steric map of ligand depicted (right).

**Figure 7:**
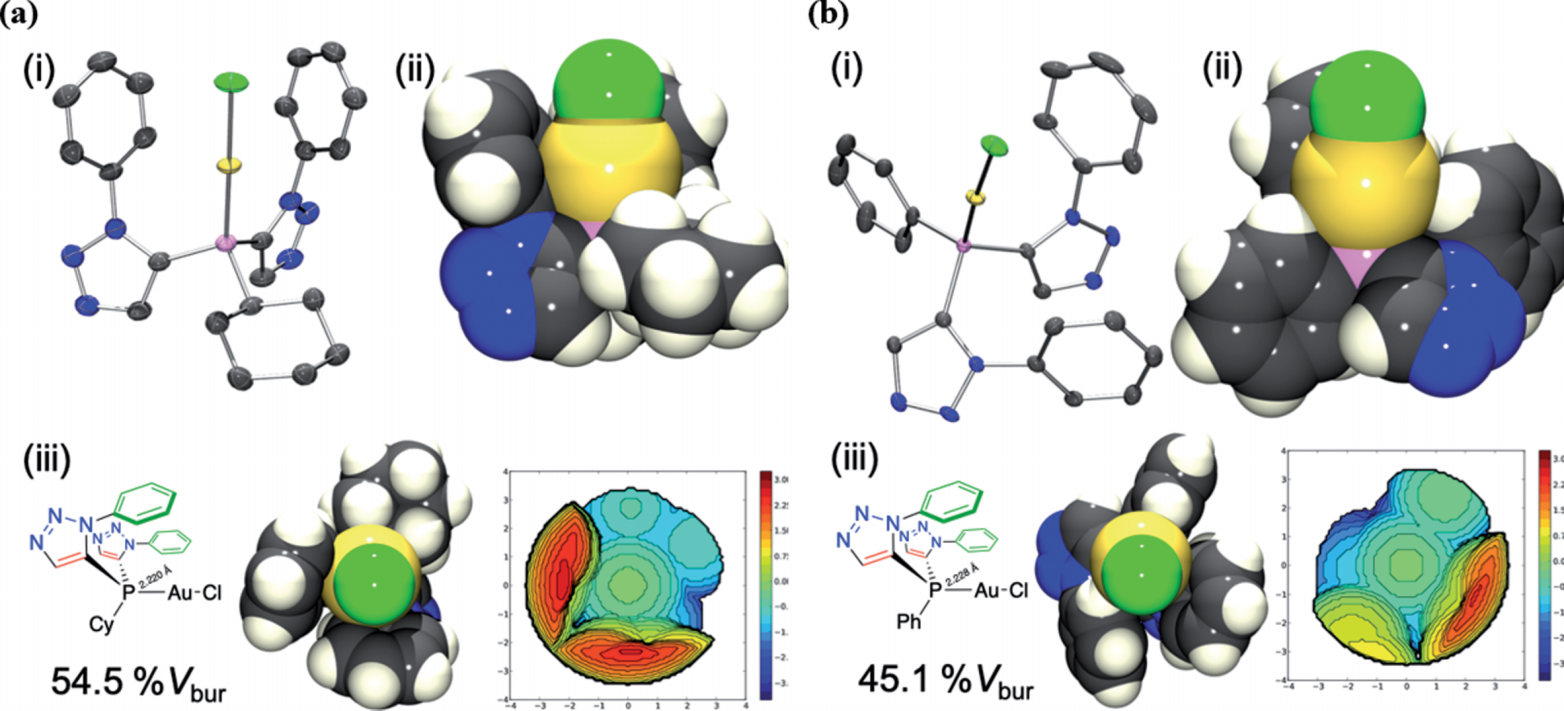
(a) Single‐crystal X‐ray diffraction structure of **9a**: (i) *Ortep* representation, ellipsoid probability 50% (rendered in *PovRay*, H‐atoms omitted for clarity). (ii) Space‐filling representation. (iii) Percentage buried volume determined from the crystal structure of **9a** (54.5%) steric map of ligand depicted (right); (b) Single‐crystal X‐ray diffraction structure of **9b**: (i) *Ortep* representation, ellipsoid probability 50% (rendered in *PovRay*, H‐atoms omitted for clarity). (ii) Space‐filling representation. (iii) Percentage buried volume determined from the crystal structure of **9b** (45.1%) steric map of ligand depicted (right).

**Figure 8:**
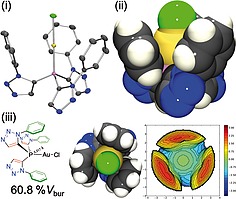
Single crystal X‐ray diffraction structure of **10**: (i) *Ortep* representation ellipsoid probability 50% (rendered in *PovRay*, H‐atoms omitted for clarity). (ii) Space‐filling representation. (iii) Percentage buried volume determined from the crystal structure of **10** (60.8%) steric map of ligand depicted (right). The unit cell contains two molecules of the complex wherein the phenyl ring centroid to gold distances are on average 3.58 Å (3.456 Å, 3.581 Å and 3.634 Å in the one molecule and 3.351 Å, 3.692 Å and 3.759 Å in the other). Between the two molecules of the unit cell a head to tail arrangement is present and the closest intermolecular Au**···**Cl intermolecular distances are 7.365 Å (see supplementary material for depictions).

**Figure 9:**
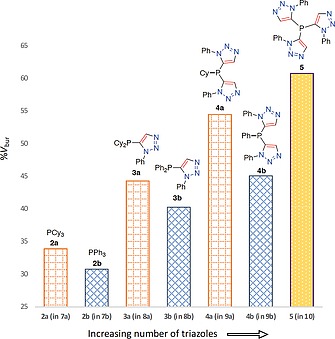
Cyclohexyl and phenyl replacement by 1‐phenyl triazole motif and corresponding; triazole number *vs*. XRD‐determined percentage buried volume [*SambVca (2.0)*].

**Figure 10:**
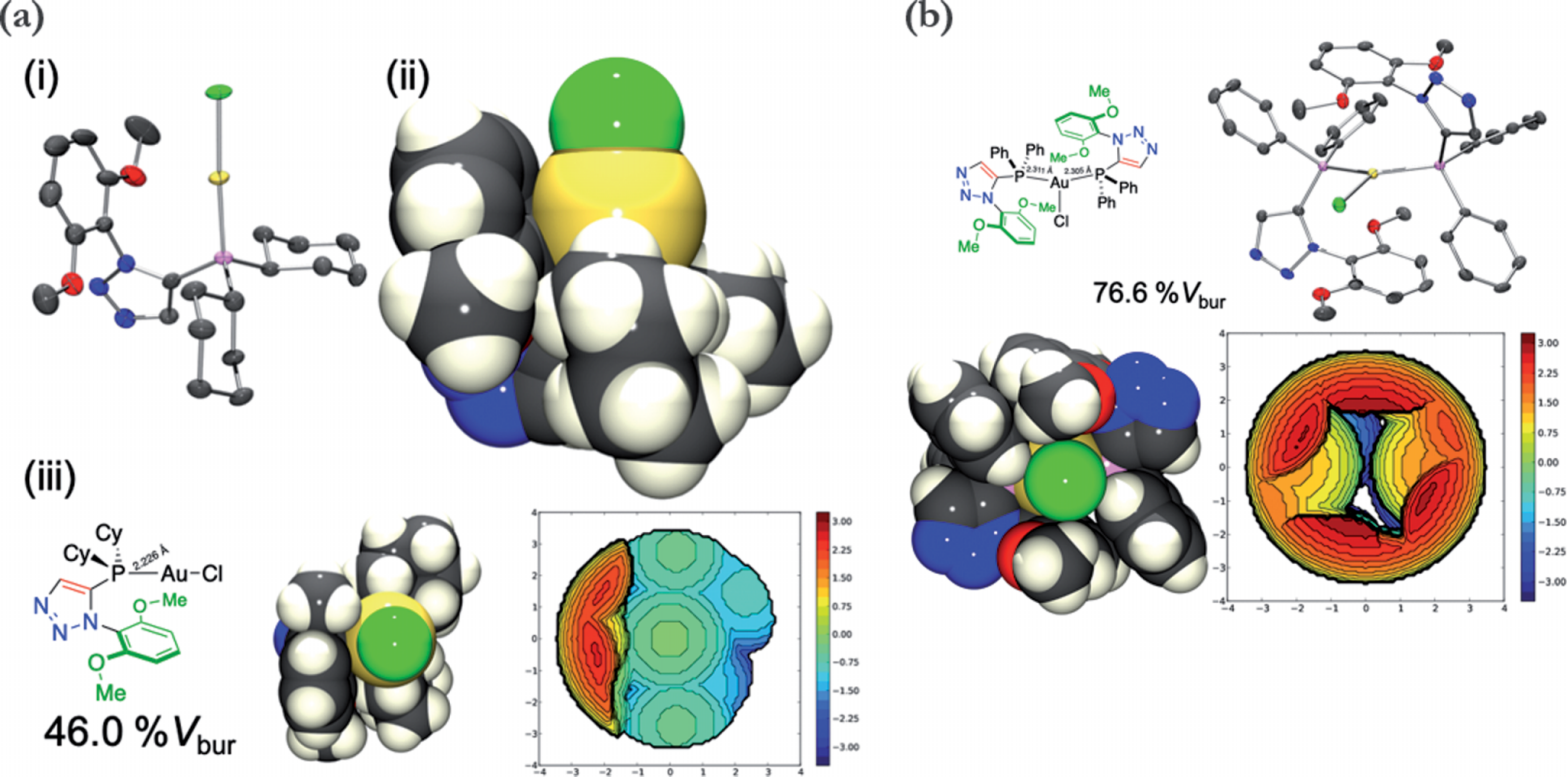
(a) Single‐crystal X‐ray diffraction structure of **11**: (i) *Ortep* representation, ellipsoid probability 50% (rendered in *PovRay*, H‐atoms omitted for clarity). (ii) Space‐filling representation. (iii) Percentage buried volume determined from the crystal structure of **11** (46.0%) steric map of ligand depicted (right). Compound **11**. ***(b)*** Single‐crystal X‐ray diffraction structure of **12**: Molecular drawing and *Ortep* representation, ellipsoid probability 50% (rendered in *PovRay*, H‐atoms omitted for clarity) upper; space‐filling representation and steric map [*SambVca (2.0)*] from which a 76.6% buried volume was determined.

The single‐crystal X‐ray structures of gold(I) chloride complexes **7a** and **7b** [gold(I) chloride complexes of tricyclohexylphosphine and triphenylphosphine] have been previously reported in the literature.^15^ The deposited PDB files were used to render images [*Ortep III for Windows* and *PovRay*, Figure 5a and Figure 5b, (i) and (ii) respectively] and determine the percentage buried volume using *SambVca (2.0)* [alternative representations of the XRD structure and a steric map thus resulting are shown in part (iii) of the corresponding figures ]. Tricyclohexylphosphine gold(I) chloride **7a** has a 33.9%*V*_bur_ whereas the triphenylphosphine gold(I) chloride complex **7b** has a 30.8%*V*_bur_. The single‐crystal X‐ray diffraction structures of **8a** (Figure 6a), **8b** (Figure 6b), **9a** (Figure 7a), **9b** (Figure 7b) and **10** (Figure 8) were obtained from the complexes synthesised herein, and similarly analysed to determine the corresponding buried volumes described by the ligands in their complexes with gold(I) chloride (presented in Figure 9). The crystal structures of the gold‐phosphorus bond lengths used in the buried volume determinations were those obtained crystallographically.

Since previously prepared mono‐triazole‐containing phosphines **1a** and **1b** were readily available to this programme of study, and as **1a** was shown previously to have been among the superior ligands of the mono‐triazole set in earlier palladium‐mediated Suzuki–Miyaura catalysis, the synthesis of gold(I) chloride complexes thereof was also attempted. The gold(I) chloride complex of **1a** (**11**) was prepared by the aforementioned dimethyl sulfide ligand exchange reaction, in good yield (95%, 0.25 mmol scale). A single crystal XRD structure of **11** was obtained (Figure 10a), and closely matched (by visual inspection) the structure of **9a**, with a 46.0%*V*_bur_. Attempts to prepare a 1:1 complex of **1b** and gold(I) chloride under the same conditions were inconclusive. However, a trigonal 2:1 ligand/gold(I) chloride complex **12** was identified by single‐crystal X‐ray diffraction crystal structure determination (Figure 10b) from a mixture of otherwise unidentified products. A 76.6%*V*_bur_ (Figure 10b) was determined from the obtained crystal structure. Attempts to prepare **12** by employing two equivalents of ligand **1b** gave inconclusive results. It is noteworthy that this 2:1 trigonal gold(I) structure is similar to crystal structures reported for [(Ph_3_P)_2_Au^(I)^Cl]^16^ and [(Ph_3_P)_2_Au^(I)^(SCN)].^17^ Furthermore a linear cationic gold(I) chloride complex [(Ph_3_P)_2_Au^+^][Cl^–^] bearing two triphenyl phosphine ligands with a fully dissociated chloride counterion also been reported.[15b] Therefore, the crystal structure of **12** is included for completeness. Attempts to prepare two and three 2,6‐dimethoxyl phenyl triazole‐containing variants of **4**(**a** or **b**) and **5** failed to deliver any desired products.^18^

### Catalysis

With the set of gold chloride complexes of Figure 2 (**7a**–**b**, **8a**–**b**, **9a**–**b** and **10**) along with complex **11** in hand, their effectiveness as precatalysts for gold‐catalysed alkyne hydration was probed (Table 1).^19^ Catalytically active cationic gold(I) species may be generated by silver‐mediated halide abstraction, and choice of appropriate counter‐anion has been shown to modulate catalytic effects.^20^ In this case, triflate was selected as a counter‐anion across all cationic gold(I) catalytic systems studied. Initially the gold‐catalysed hydration of dec‐1‐yne (**13a**) was performed by *in situ* preactivation of 0.5 mol% of the corresponding gold complex through silver triflate‐mediated halide abstraction, using an excess of silver triflate in methanol (or as explained below, dichloromethane in the case of **10**), alkyne **13a** was stirred in methanol to which the preactivated catalyst solution was added. Since complex **10** was not well solubilised in methanol a protocol of activation in dichloromethane and dilution in methanol was deployed. To facilitate comparison in the subsequent reactions with lower catalyst loadings (0.25 and 0.05 mol% of gold complex), the dichloromethane activation and methanol dilution protocol was used in those cases. Following the addition of water (2 equiv.) all reactions were heated at 80 °C in a sealed tube for two hours and conversions to ketone **14a** were determined by gas chromatography.

**Table 1:** Screening of ligands for gold‐catalysed hydration of dec‐1‐yne (**13a**) to 2‐decanone (**14a**)

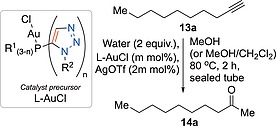							
Entry	L‐	R^1^	R^2^	Triazole	Conversion [%] (mmol%)		
	AuCl			No. (*n*)	0.50^a^	0.25^b^	0.05^b^
1	**7a**	Cy	–	0	> 99	86	10
2	**8a**	Cy	Ph	1	> 99	***> 99***	9
3	**9a**	Cy	Ph	2	> 99	46	3
4	**10**	–	Ph	3	32^b^	11	4
5	**9b**	Ph	Ph	2	> 99	26	2
6	**8b**	Ph	Ph	1	> 99	57	4
7	**7b**	Ph	–	0	> 99	52	3
8	**11**	Cy	2,6‐DMP^c^	1	> 99	***> 99***	10

a Methanol used as solvent, unless otherwise started.

b Catalyst precursor initially solubilised in dichloromethane and dispersed in methanol, such that the resulting solvent composition was 10% CH_2_Cl_2_ and 90% MeOH.

c 2,6‐DMP = 2,6‐dimethoxyphenyl. These conditions were deployed across all tests experiments at 0.25 and 0.05 mol% gold(I) complex loading.

At the highest catalyst loading of 0.5 mol% only the bulkiest complex (**10**) failed to give complete conversion to product **14a** (Table 1, entry 10 *vs*. other entries in same table ). When lower catalyst loadings were probed 0.05 mol% of gold(I) complexes proved to be too low to achieve satisfactory conversion within two hours (the maximum conversion observed across the set was 10% under these conditions). Good gradation across the test series was seen at a 0.25 mol% catalyst loading (Table 1) and confirmed the superior ligands, for this gold‐catalysed transformation, to be those with one triazole substituent. The dicyclohexylphosphine‐containing complex **8a** afforded quantitative conversion to **14a** (Table 1, entry 2). The corresponding mono‐triazole‐diphenylphosphine‐containing complex **8b** gave the best conversion to **14a** among the phenyl‐appended phosphine series of 57% (Table 1, entry 6). The three triazole congener (**10**) (Table 1, entry 4) gave only 11% conversion under the same conditions. Pleasingly, however, catalysts derived by halide abstraction in the same manner from complex **11** gave quantitative conversion of **13a** to **14a** (Table 1, entry 8).

Synthetic modification of phosphorus‐containing ligands can result in significant differences in the outcomes of reactions catalysed by their corresponding cationic gold(I) complexes.[4c], [19a], 21 The regioselectivity of hydration of unsymmetrical internal alkynes has been previously probed by Nolan and co‐workers who identified anti‐Markovnikov‐selective gold(I)‐carbene complex‐catalysed hydration.^22^ As such, a model reaction, namely the hydration of unsymmetrical internal alkyne **15** was selected to probe selectivity that might arise from the structural modifications across the series of gold(I) complexes of phosphino‐triazoles prepared in this study (Table 2). The reaction of water at the benzylic position (**15**‐a) represents the generally expected (Markovnikov) outcome, with selective reaction at the alternate alkyne position (**15**‐b, anti‐Markovnikov) being more challenging.

**Table 2:** Selectivity of the hydration of unsymmetrical alkyne **15** to give ketones **16a** and/or **16b**

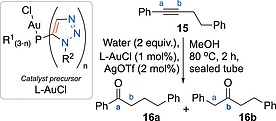						
Entry	L‐AuCl	R^1^	R^2^	Triazole	Conv.	Ratio^a^
				No. (*n*)	[%]	(**16a**/**16b**)
1	7a	Cy	–	0	> 99	3.5:1
2	8a	Cy	Ph	1	> 99	3.2:1^b^
3	9a	Cy	Ph	2	> 99	4.3:1^b^
4	10	–	Ph	3	64	3.8:1
5	9b	Ph	Ph	2	> 99	3.3:1
6	8b	Ph	Ph	1	> 99	4.2:1
7	7b	Ph	–	0	> 99	4.0:1
8	11	Cy	2,6‐DMP^d^	1	95^c^	2.3:1

a (a) Ratio determined by GC analysis of reaction mixture.

b Ratio determined by analysis of the proton NMR spectrums of mixtures of **16a** and **16b** obtained from the reactions.

c Complete consumption of **16** was observed, the product mixture contained 5% of a dimethyl acetal adduct as determined by GC/GC–MS.

d 2,6‐DMP = 2,6‐dimethoxyphenyl.

Compound **15** was added to a mixture of catalyst precursor (1 mol%) (which had undergone silver triflate (2 mol%)‐mediated *in situ* halide abstraction) in a degassed methanol/water mixture.^23^ Consumption of **15** and the ratio of products **16a**:**16b** was determined by ^1^H NMR spectroscopy or gas chromatography. That quantitative conversion was observed in all‐but‐one case (tris‐triazole complex **10**, Table 2 entry 4),[19a] is in keeping with the results from the hydration of dec‐1‐yne **13a** (Table 1). All complexes showed a preference for Markovnikov addition at the benzylic position of the alkyne (position [a]). Whilst all cases gave product **16a** in preference to **16b** (ranging from 4.2:1 to 2.3:1 ratios of products **16a**/**16b**), it is noteworthy that use of complexes **8a** and **11** as catalyst precursors (Table 2, entries 2 and 8 respectively) afforded a greater proportion of the challenging anti‐Markovnikov product **16b**.

Having established that, for gold(I)‐mediated hydration of alkynes, cationic gold(I) complexes bearing dicyclohexyl monotriazole ligands **1a** and **3a** (complexes **11** and **8a** respectively) were superior in terms of activity (Table 1); and the **1a** derived catalyst gave regioselectivity of <3:1 for **16a**:**16b** (Table 2); complex **11** was deployed in a brief substrate scope survey (Table 3).

**Table 3:**
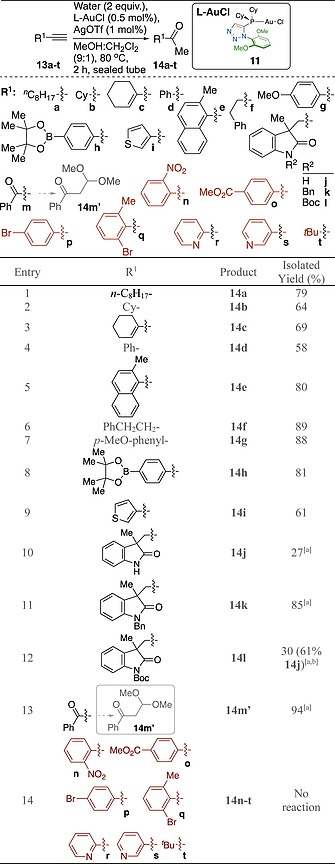
Substrate scope survey for triazole‐appended phosphine ligate gold(I) catalysed alkyne hydration

1 mol% L‐AuCl and 2 mol% AgOTf.

Significant Boc‐deprotection observed, isolated yield of **14j** given in parenthesis.

Linear alkyl‐alkynes **13a** and **13f** (Table 3, entries 1 and 6) gave slightly higher yields than the cyclic alkane ethynylcyclohexane **13b** (Table 3, entry 2), 79 and 89% *vs*. 64% respectively. 1‐Ethynylcyclohex‐1‐ene **13c** (Table 3, entry 3) gave a similar yield (69%) to **13b**. Phenylacetylene **13d** (Table 3, entry 4) gave 58% yield, with the yield for the methylnaphthyl derivative **13e** being somewhat better (80% yield, Table 3, entry 5). The yield for use of *para*‐methoxy phenyl acetylene **13g** was better than for phenylacetylene (88 *vs*. 58% yield, Table 3, entries 7 & 4 respectively). Pleasingly an aryl boronic ester **13g** was accommodated under the reaction conditions employed (81% yield, Table 3, entry 8), as was thiophene derivative **13i**, albeit in a slightly lower isolated yield (61% yield, Table 3, entry 9). Quaternary oxindoles containing alkynes have been of interest to co‐authors of this report and their availability to this programme allowed **13j**–**l** to be probed as substrates for gold(I) catalysed hydration (Table 3, entries 10 to 12).^24^ To obtain appreciable conversion to isolable oxindole‐containing products catalyst loading had to be increased to 1 mol%. The N‐H bearing substrate **13j** was converted to product **14j** in relatively low conversions (27%, Table 3, entry 10). The *N*‐benzyl analogue **13k** fared better giving 85% isolated yield (Table 3, entry 11). A major side product arose from *in situ* removal of the Boc‐group upon reaction of **13l** (Table 3, entry 12) giving rise to a mixture of desired product **14l** (in 30% isolated yield) and deprotected product **14j** (61% isolated yield). The reaction of **14m** gave the dimethyl acetal of overall reaction at the alkyne terminus in 94% isolated yield (**14m′**, Table 3, entry 13). Nitroarene **13n**, aryl ester **13o**, aryl bromides **13p** and **13q**, pyridyl substrates **13r** and **13s** and tertiary butyl **13t** appended alkynes all failed to give any detectable hydration products under the conditions employed (Table 3, combined entry 14). Further optimisation to facilitate these transformations was not conducted.

## Conclusions

1,2,3‐Triazoles bearing 1‐aryl and 5‐phosphino functionalities were prepared, wherein the phosphorus atom was appended to one, two or three such triazole motifs. In addition, the corresponding gold(I) chloride coordination complexes bearing phosphino triazoles [with ancillary cyclohexyl or phenyl groups on phosphorus to complete the valence requirement of phosphorus(III) where required] were also prepared and their relative bulkiness determined using the online tool *SambVca (2.0)*. Two more gold(I) chloride complexes were prepared from ligands containing a phosphorus atom appended by one triazole of the same general formula, where the 1‐aryl substituent on the triazole was 2,6‐dimethoxy phenyl. These complexes, along with complexes of tricyclohexyl phosphine and triphenyl phosphine were *in situ* converted into their corresponding cationic gold(I) triflate phosphine ligated congeners and compared as alkyne hydration catalysts. Increasing the “triazole number” about the phosphorus atom confirmed that, among the triazole appended phosphines investigated, the monotriazole‐containing phosphines were the most effective ligands for cationic gold(I)‐catalysed hydration of alkynes.

In summary, the data derived from the above experimentation show triazole‐phosphine ligand **1a** to be the most promising of the ligands reported. The ease of synthesis and modification of the triazole ligand framework should prove useful in future ligand design, screening and optimisation campaigns.

**Supporting Information** (see footnote on the first page of this article): General and experimental procedures are available as supporting information, which also includes a summary of the crystallographic data collection and analysis, and further analysis of structural features of the crystal structures of the complexes discussed. All crystal structures disclosed in this report have had the corresponding data deposited at the CCDC with deposition numbers 1922101–1922111. Experimental procedures, including the synthesis of non‐commercially available alkynes, characterisation data and spectroscopic information are available in supporting information as are selected, preliminary palladium‐catalysis findings. Citations therein refer to methods, data and software used.^25^ A pre‐peer‐review version of this manuscript has been submitted to a preprint repository.^26^

CCDC 1922101 (for **4a**), 1922102 (for **4b**), 1922103 (for **5**), 1922104 (for **8a**), 1922105 (for **8b**), 1922106 (for **9a**), 1922107 (for **9b**), 1922108 (for **10**), 1922109 (for **11**), 1922110 (for **12**), and 1922111 (for **S5** (see supplementary material)) contain the supplementary crystallographic data for this paper. These data can be obtained free of charge from The Cambridge Crystallographic Data Centre.

**Author Contributions**

All authors contributed in varying degrees to planning the experiments, evaluating results and writing of the manuscript, specific contributions in addition to this are listed for each co‐author in alphabetical order: BRB helped direct aspects of the research and gave input and critical assessment throughout the progress of the project; PWD suggested experiments, supervised and provided critical assessment for aspects of the work; JSF led and co‐conceived the project, providing critical assessment of data, day‐to‐day project management and oversight, directed most aspects throughout, supervised most of the experimental work and wrote the majority of the manuscript; FM and TJS synthesised some of the alkynes used in Table 3; HvN contributed to preliminary studies detailed in the supplementary material and some aspects of mass spectrometry of complexes; MGW advised on the preparation of gold complexes and conducted experiments of Table 2; YZ co‐conceived aspects of the project, conducted all ligand synthesis and most of the reactions, drafted a proportion of the ESI, offered critical suggestions and conducted the XRD data collection and analysis herein.

## Supporting information

Supporting Information
