# Supplementary material for: Balancing Bulkiness in Gold(I) Phosphino‐triazole Catalysis
Source: European J Org Chem. 2019 Jul 30;2019(31-32):5540–8. doi: 10.1002/ejoc.201900850 (PMC6774259; doi:10.1002/ejoc.201900850)
Supplement: Supplementary file 1 — Supporting Information [file EJOC-2019-5540-s001.pdf]

EurJOC
European Journal of Organic Chemistry

Supporting Information

Balancing Bulkiness in Gold(I) Phosphino-triazole Catalysis

Yiming Zhao, Matthew G. Wakeling, Fernanda Meloni, Tze Jing Sum, Huy van Nguyen, Benjamin R. Buckley, Paul W. Davies, and John S. Fossey*

ejoc201900850-sup-0001-SupMat.pdf

GENERAL EXPERIMENTAL 1
SYNTHETIC PROCEDURES AND PROTOCOLS 3
GENERAL PROCEDURES 3
Phosphine Preparation Method (PPM) 3
Preparation of Phosphine Gold(I) Chloride Complexes (PGC) 3
SYNTHETIC PROTOCOLS AND EXPERIMENTAL DETAILS 3
*Synthesis of dimethylsulfide gold(I)chloride (**S1**)* 3
*Synthesis of 5,5'-(cyclohexylphosphanediyl)bis(1-phenyl-1H-1,2,3-triazole) (**4a**)* 3
*Synthesis of 5,5'-(penylphosphanediyl)bis(1-phenyl-1H-1,2,3-triazole) (**4b**)* 4
*Synthesis of tris(1-phenyl-1H-1,2,3-triazol-5-yl)phosphane (**5**)* 4
*Synthesis of chloro[5-(dicyclohexylphosphanyl)-1-phenyl-1H-1,2,3-triazole] gold(I) (**8a**)* 4
*Synthesis of chloro[5-(diphenylphosphanyl)-1-phenyl-1H-1,2,3-triazole] gold(I) (**8b**)* 5
*Synthesis of chloro [5,5'-(cyclohexylphosphanediyl)bis(1-phenyl-1H-1,2,3-triazole)] gold(I) (**9a**)* 5
*Synthesis of chloro[5,5'-(penylphosphanediyl)bis(1-phenyl-1H-1,2,3-triazole)] gold(I) (**9b**)* 5
*Synthesis of chloro[tris(1-phenyl-1H-1,2,3-triazol-5-yl)phosphane] gold(I) (**10**)* 5
*Synthesis of chloro[5-(dicyclohexylphosphanyl)-1-(2,6-dimethoxyphenyl)-1H-1,2,3-triazole] gold(I) (**11**)* 6
MATERIALS PREPARED FOR SUBSTRATE SCREENING 6
*Synthesis of 2-methyl-1-naphthaldehyde (**S2**)* 6
*Synthesis of dimethyl-1-diazo-2-oxopropylphosphonate (**S3**)* 6
*Synthesis of 1-ethynyl-2-methylnaphthalene (**13e**)* 7
*Synthesis of 3-methyl-3-(prop-2-yn-1-yl)indolin-2-one (**13j**)*[13] 7
*Analysis of 3-methyl-1,3-di(prop-2-yn-1-yl)indolin-2-one (**S4**) obtained as described above* [13] 8
*Synthesis of 1-benzyl-3-methyl-3-(prop-2-yn-1-yl)indolin-2-one (**13k**)*[13] 8
*Synthesis of tert-butyl 3-methyl-2-oxo-3-(prop-2-yn-1-yl)indoline-1-carboxylate (**13l**)*[13] 8
ANALYSIS OF MATERIALS RESULTING FROM SUBSTRATE SCREENING IN CATALYSIS 9
*Analysis of decan-2-one (**14a**)*[14] 9
*Analysis of 1-cyclohexylethan-1-one (**14b**)* 9
*Analysis of 1-(cyclohex-1-en-1-yl)ethan-1-one (**14c**)* 9
*Analysis of acetophenone (**14d**)*[14] 9
*Analysis of 1-(2-methylnaphthalen-1-yl)ethan-1-one (**14e**)* 10
*Analysis of 4-phenylbutan-2-one (**14f**)*[15] 10
*Analysis of 1-(4-methoxyphenyl)ethan-1-one (**14g**)*[14] 10
*Analysis of 1-(4-(4,4,5,5-tetramethyl-1,3,2-dioxaborolan-2-yl)phenyl)ethan-1-one (**14h**)* 10
*Analysis of 1-(thiophen-3-yl)ethan-1-one (**14i**)* 10
*Analysis of 3-methyl-3-(2-oxopropyl)indolin-2-one (**14j**)* 10
*Analysis of 1-benzyl-3-methyl-3-(2-oxopropyl)indolin-2-one (**14k**)* 11
*Analysis of tert-butyl 3-methyl-2-oxo-3-(2-oxopropyl)indoline-1-carboxylate (**14l**)* 11
*Analysis of 3,3-dimethoxy-1-phenylpropan-1-one (**14m'**)* 11
MATERIALS SYNTHESISED FOR OR ARISING FROM REGIOSELECTIVITY STUDY 11
*Synthesis of but-1-yne-1,4-diyldibenzene (**15**)* 11
*Procedure resulting in mixtures of **16a** and **16b*** 12
*Analysis of 1,4-diphenylbutan-1-one (**16a**)* 12
*Analysis of 1,4-diphenylbutan-2-one (**16b**)* 12
PRELIMINARY PALLADIUM-CATALYSED CROSS-COUPLING FINDINGS 13
General procedure for palladium-catalysed C-N cross-coupling 14
*Synthesis of N,4-dimethyl-N-phenylaniline **S8a*** 14
*Synthesis of N,N-dibenzyl-4-methylaniline **S8b*** 15
*Synthesis of N-butyl-4-methylaniline **S8c*** 15
*Synthesis of 4-(p-tolyl)morpholine **S8d*** 15
NMR SPECTRUMS 16
X-RAY CRYSTALLOGRAPHIC INFORMATION 63
*Compound **4a*** 63
*Compound **4b*** 63
*Compound **5*** 63
*Compound **8a*** 64
*Compound **8b*** 64
*Compound **9a*** 65
*Compound **9b*** 65
*Compound **10*** 65
*Compound **11*** 66
*Compound **12*** 67
*Compound **S5** (S-Phos Gold(I) chloride complex)* 68
SUPPLEMENTARY REFERENCES 69

General Experimental

Unless otherwise stated, commercially available solvents and reagents were used as obtained, without further purification. At the University of Birmingham, ^{1}H NMR spectrums were recorded on Bruker AVIII300/400/500 NMR spectrometers at 300, 400 and 500 MHz respectively. Proton decoupled ^{19}F NMR spectrums were recorded on a Bruker AVIII300 NMR spectrometer at 282 MHz. Both proton-coupled and proton-decoupled ^{31}P NMR spectrums were recorded on a Bruker AVIII300 NMR spectrometer at 131 MHz. The ^{31}P chemical shifts are reported, unless

otherwise stated, as obtained from the proton decoupled spectrum, where used for additional structural corroboration both proton-coupled and proton-decoupled spectral data are reported, and where both were obtained both processed spectra are included. Proton decoupled ^{13}C NMR spectrums were recorded at room temperature on Bruker AVIII300/400/500 NMR spectrometers at 75, 101 and 126 MHz respectively.[1] The proton decoupled ^{13}C NMR signal quality was superior when using the UDEFT pulse sequence, as such ^{13}C NMR signals are typically reported as obtained by use of the UDEFT technique.[2] Furthermore, assignments of ^{13}C NMR spectroscopy signals in some cases was facilitated by obtaining spectrums using the J-MOD pulse sequence, the sign relating to proton substitution is deployed as follows: Positive *C*H and *C*H_3 (denoted [+]); and negative C_{quat} and *C*H_2 (denoted [-]), relative in this case to solvent *C*DCl_3 [-].[3] The UDEFT pulse sequence was also used to enhance intensity of ^{13}C signals in some cases, where a signal was observed utilising UDEFT but not observed with J-MOD the signal is give designation [u]. At Loughborough University, ^1H proton and ^{13}C (proton decoupled) carbon NMR spectra were measured at 400 and 100 MHz respectively, using either a Bruker Avance 400 or Jeol ECS 400 spectrometer (**13j-l** and **S4**). In all cases relevant coupling constants (*J*) are expressed in Hertz (Hz).[4] Multiplicities are reported as singlet (s), doublet (d), triplet (t), quartet (q), septet (sept), multiplet (m) and broad (br), the designation app is used to describe the *apparent* appearance of a signal believed to consist of overlapping signals or displaying other phenomena to reveal an apparent signal inconsistent with expected multiplicity.[5] NMR spectroscopic data was processed using *Mestrenova v10.02.-15465* and/or *Topspin v3.5*. The chemical shifts for each signal in the proton NMR spectrums are reported as chemical δ (ppm) relative to either tetramethylsilane (TMS) where $\delta_{(TMS)}$ = 0.00 or a residual solvent peak.[6] The chemical shifts for each signal in the ^{13}C NMR spectrums are reported relative to signals of the solvent employed.[6] Mass spectrometry was conducted using a *Waters LCT Time of Flight Mass Spectrometer* (electrospray), a *Waters GCT Premier Time of Flight Mass Spectrometer* (EI GC/MS) or using *Bruker micrOTOF-QII* (ESI+) by direct injection from *LC ultimate 3000*. Infrared spectra were recorded at room temperature on either a *PerkinElmer 100FT-IR* spectrometer or a *Varian 660-IR* spectrometer with ATR attachments; a corresponding thin film was obtained by evaporation of dichloromethane solvent from a given sample. Melting points are uncorrected and were carried out in triplicate using a *Stuart SMP10* melting point apparatus and average values reported as a range. Column (flash) chromatography was carried out using an *Isco Combiflash EZ Prep* or *Interchim PuriFlash XS 420* automated chromatography apparatus. Mobile and stationary phases are described in the general methods or the experimental procedures. Chromatographic traces were recorded at two wavelengths (254 nm and 288nm). Neighbouring tubes identified as pure were double-checked by separate TLC analysis, prior to being combined into single fractions and evaporated to dryness *in vacuo*. Compounds synthesised that have been previously reported gave satisfactory correlation with spectroscopic observations given in the literature and cited herein. Compounds **1a**, **3a**, **3b** and **6**, were derived from the same batch as that prepared for and reported in a previous publication.[7]

Synthetic Procedures and Protocols

General Procedures

Phosphine Preparation Method (PPM)

Under anhydrous conditions and protected from air, a solution of the corresponding triazole (1 equiv.) in tetrahydrofuran (0.04 M) was stirred at -78 °C. To which *n*-butyl lithium (1.2 equiv., 2.0 M in tetrahydrofuran) was added dropwise.[8] The reaction mixture was stirred for two hours at this temperature and then the phenyl- or cyclohexyl- phosphine dichloride (0.5 equiv.) was carefully added. After addition was complete, the reaction mixture was allowed to slowly warm to room temperature. After stirring at room temperature for a further ten hours, the volatiles were removed *in vacuo* to afford a crude residue. The residue thus obtained, was purified by flash chromatography (*Isco CombiFlash,* silica 50-60 µm, or *Interchim XS 420*, 20 µm, 4 or 12 g column, gradient elution; hexane (100%) to hexane (20%): ethyl acetate (80%), detection absorption at 254 and 288 nm) to afford the corresponding products.

Preparation of Phosphine Gold(I) Chloride Complexes (PGC)

Dimethylsulfide gold(I)chloride **S1** and corresponding phosphine (1 equiv.) were added to a dried flask. Dichloromethane was added and the solution thus obtained (to achieve a concentration of 0.1 M), which was stirred at room temperature, under argon, for two hours. After this time an at least equal volume amount of hexane was added resulting formation a white precipitate. The precipitate was collected *via* filtration and washed with a little hexane to yield the desired complexes.

Synthetic Protocols and Experimental Details

Synthesis of dimethylsulfide gold(I)chloride (**S1**)

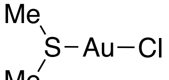

S1

Under anhydrous conditions and protected from air, a solution of the potassium tetrachloroaurate (377 mg, 1.0 mmol 1 equiv.) in methanol (1 mL) was added to a solution of dimethyl sulfide (0.22 mL, 3.0 mmol 3 equiv.) in methanol (4 mL). The resulting mixture was stirred at room temperature, protected from light, for two hours. The resulting white solid was collected by filtration and washed with cold methanol (~2 mL) to deliver the desired product as a white solid (248 mg, 84%).

Synthesis of 5,5'-(cyclohexylphosphanediyl)bis(1-phenyl-1*H*-1,2,3-triazole) (**4a**)

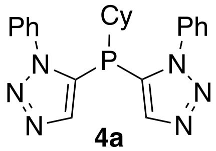

4a

Following *PPM General Procedure* on a 1.47 mmol scale (triazole **6** used in synthesis): White solid (143 mg, 71%); m.p. 169-171 °C; ^{1}H NMR (400 MHz, $CDCl_3$) δ 7.78 (d, *J* 0.7, 2H, Trz*H*), 7.53-7.48 (m, 2H), 7.44 (t, *J* 7.4, 4H), 7.21-7.18 (m, 4H), 2.40-2.30 (m, 1H), 1.73-1.59 (m, 5H), 1.28-1.03 (m, 6H); ^{31}P NMR (121 MHz, $CDCl_3$) δ 65.94; ^{13}C NMR (101 MHz. $CDCl_3$) δ 139.04, 136.38, 132.79 (d, $^{1}J_{CP}$ 19.2), 129.96, 129.29, 125.87 (d, $^{4}J_{CP}$ 4.0), 37.82 (d, $^{1}J_{CP}$ 4.4), 29.42 (d, J_{CP} 16.1), 26.10 (d, J_{CP} 12.5), 25.74; IR ν (cm^{-1}) 3112, 3062, 2927, 2852, 1596, 1498, 1450; TOF MS ES+ m/z: 403.2 $[M+H]^+$, 375.2 [M+H-

2N]$^+$; HR-MS calc. $[C_{22}H_{24}N_6P]^+$ 403.1795 obs. 403.1799. Structure corroborated by single crystal X-ray diffraction determination.

Synthesis of 5,5'-(penylphosphanediyl)bis(1-phenyl-1*H*-1,2,3-triazole) (**4b**)

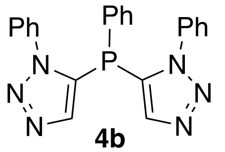

4b

Following *PPM General Procedure* on a 1.0 mmol scale (triazole **6** used in synthesis): White solid (104 mg, 54%); m.p. 165-167 °C; ^{1}H NMR (400 MHz, $CDCl_3$) δ 7.47-7.38 (m, 12H, Ar*H*), 7.28-7.24 (m, 5H, Ar*H*);^{31}P NMR (121 MHz, $CDCl_3$) δ 64.31; ^{13}C NMR (126 MHz. $CDCl_3$) δ 140.64, 136.32, 133.69 d, J_{CP} 23.0), 132.63 (d, J_{CP} 12.2), 131.27, 129.95, 129.60, 129.53, 129.42, 125.00 (d, J_{CP} 4.3); IR ν (cm^{-1}) 3107, 3058, 1596, 1498, 1436, 1286, 1229; TOF MS ES+ m/z: 397.1 [M+H]$^+$, 419.1 [M+Na]$^+$; $[C_{22}H_{18}N_6P]^+$ 397.1325 obs. 397.1330. Structure corroborated by single crystal X-ray diffraction determination.

Synthesis of tris(1-phenyl-1*H*-1,2,3-triazol-5-yl)phosphane (**5**)

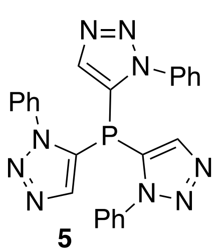

5

Under anhydrous conditions and protected from air, a solution triazole **6** (145 mg, 1.0 mmol) in tetrahydrofuran (0.04 M) was stirred at -78 °C. To which *n*-butyl lithium (1.2 equiv., 2.0 M in tetrahydrofuran) was added dropwise.[8] The reaction mixture was stirred for two hours at this temperature and then phosphorus trichloride (0.33 equiv.) was carefully added. After addition was complete, the reaction mixture was allowed to slowly warm to room temperature. After stirring at room temperature for a further ten hours, the volatiles were removed *in vacuo* to afford a crude residue. The residue thus obtained, was purified by flash chromatography (*Isco CombiFlash,* silica 50-60 μm, gradient elution hexane (100%) to hexane (0%): ethyl acetate (100%), detection 254 and 288 nm absorption) to afford the corresponding product. White solid (108 mg, 77%); mp. 186-188 °C; ^{1}H NMR (400 MHz, $CDCl_3$) δ 7.63 (d, $^3J_{CP}$ 0.9, 3H, Trz*H*), 7.49 (tt, *J* 7.4, 1.2, 3H), 7.42 (t, *J* 7.9, 6H), 7.17-7.14 (m, 6H); ^{31}P NMR (121 MHz, $CDCl_3$) δ -93.52; ^{13}C NMR (101 MHz, $CDCl_3$) δ 141.07, 135.72, 130.45, 129.72, 129.43 (d, $^1J_{CP}$ 4.2), 124.90 (d, $^4J_{CP}$ 4.3); IR ν (cm^{-1}) 3105, 1595, 1497; TOF MS ES+ m/z: 464.2 [M+H]$^+$, 486.1 [M+Na]$^+$, 949.3 [2M+Na]$^+$; HR-MS calc. $[C_{24}H_{18}N_9PNa]^+$ 486.1315, obs. 486.1323. Structure corroborated by single crystal X-ray diffraction determination.

Synthesis of chloro[5-(dicyclohexylphosphanyl)-1-phenyl-1H-1,2,3-triazole] gold(I) (**8a**)

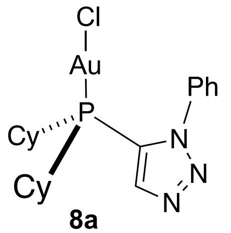

8a

Following *PGC General Procedure* on a 0.1 mmol scale. White solid (48 mg, 83%); m.p. >250 °C; ^{1}H NMR (400 MHz, $CDCl_3$) δ 7.99 (s, 1H, Trz*H*), 7.73 (t, *J* 7.51, 1H), 7.63 (t, *J* 7.8, 2H), 7.35 (d, *J* 7.6, 2H), 2.21-2.07 (m, 2H), 2.01-1.92 (m, 2H), 1.92-1.80 (m, 4H), 1.79-1.64 (m, 4H), 1.43-1.10 (m, 10H); ^{31}P NMR (121 MHz, $CDCl_3$) δ 22.14; ^{13}C NMR (101 MHz. $CDCl_3$) δ 139.18 (d, J_{CP} 7.2), 135.78, 131.65, 130.15, 127.54, 126.17 (d, J_{CP} 52.8), 35.63 (d, J_{CP} 35.8), 29.78 (d, J_{CP} 3.5), 28.58, 26.28 (d, J_{CP} 5.0), 26.13, 25.41; IR ν (cm^{-1}) 2927, 2852, 1595, 1497, 1448, 1287; TOF MS ES+ m/z: 574.10 [M+H]$^+$; HR-MS calc. $[C_{20}H_{29}AuClN_3P]^+$ 574.1448 obs. 574.1445. Structure corroborated by single crystal X-ray diffraction determination.

Synthesis of chloro[5-(diphenylphosphanyl)-1-phenyl-1H-1,2,3-triazole] gold(I) (**8b**)

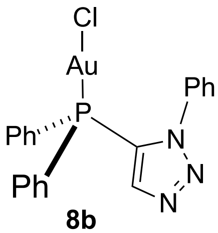

8b

Following *PGC General Procedure* on a 0.1 mmol scale. White solid (43 mg, 86%); m.p. 230 °C (decomp); ^{1}H NMR (400 MHz, $CDCl_3$) δ 7.68-7.51 (m, 12H), 7.44 (t, *J* 7.9, 2H), 7.19 (d, *J* 7.4, 2H); ^{31}P NMR (121 MHz, $CDCl_3$) δ 8.61; ^{13}C NMR (101 MHz. $CDCl_3$) δ 141.53 (d, J_{CP} 10.6), 135.55, 133.97 (d, J_{CP} 15.2), 133.15 (d, J_{CP} 2.5), 131.23, 129.86, 129.82, 129.73, 126.33, 126.55 (d, J_{CP} 67.3); IR ν (cm^{-1}) 3056, 2923, 1595, 1498, 137, 1287, 1102; TOF MS ES+ m/z: 562.06 $[M+H]^+$; HR-MS calc. $[C_{20}H_{17}AuClN_3P]^+$ 562.0509 obs. 562.0499. Structure corroborated by single crystal X-ray diffraction determination.

Synthesis of chloro [5,5'-(cyclohexylphosphanediyl)bis(1-phenyl-1*H*-1,2,3-triazole)] gold(I) (**9a**)

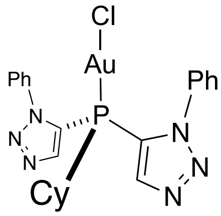

9a

Following *PGC General Procedure* on a 0.1 mmol scale. White solid (51 mg, 80%); m.p. 235-237 °C; ^{1}H NMR (400 MHz, $CDCl_3$) δ 7.84 (s, 2H, Trz*H*), 7.69 (t, *J* 7.6, 2H), 7.55 (t, *J* 7.9, 4H), 7.08 (d, *J* 7.3), 2.50-2.39 (m, 1H), 1.97-1.86 (m, 2H), 1.82-1.73 (m, 3H), 1.49-1.24 (m, 5H); ^{31}P NMR (121 MHz, $CDCl_3$) δ -11.87; ^{13}C NMR (101 MHz. $CDCl_3$) δ 140.42 (d, $^{2}J_{CP}$ 13.3), 135.01, 131.88, 130.16, 126.97, 125.74 (d, J_{CP} 67.1), 38.15 (d, J_{CP} 41.0), 29.40 (d, J_{CP} 4.6), 25.79 (d, J_{CP} 16.7), 25.03; IR ν (cm^{-1}) 3110, 3063, 2932, 2854, 1594, 1496, 1452; TOF MS ES+ m/z: 635.07 $[M+H]^+$; HR-MS calc. $[C_{22}H_{24}AuClN_6P]^+$ 635.1149 obs. 635.1143. Structure corroborated by single crystal X-ray diffraction determination.

Synthesis of chloro[5,5'-(penylphosphanediyl)bis(1-phenyl-1*H*-1,2,3-triazole)] gold(I) (**9b**)

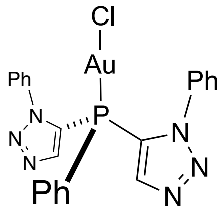

9b

Following *PGC General Procedure* on a 0.1 mmol scale. White solid (37 mg, 58%); m.p. 176-178 °C; ^{1}H NMR (400 MHz, $CDCl_3$) δ 7.91-7.44 (m, 13H, Ar*H*), 7.19-7.02 (m, 4H, Ar*H*); ^{31}P NMR (121 MHz, $CDCl_3$) δ -16.87; ^{13}C NMR (126 MHz. $CDCl_3$) δ 141.43 (d, J_{CP} 10.8), 135.04, 134.40, 133.99 (d, J_{CP} 17.3), 131.70, 131.40 (d, J_{CP} 13.6), 130.10, 127.09, 126.49, 126.28; IR ν (cm^{-1}) 3111, 3059, 1594, 1467, 1438, 1289; TOF MS ES+ m/z: 397.10 $[M\text{-}Au\text{-}Cl+H]^+$, 629.01 $[M+H]^+$; HR-MS calc. $[C_{22}H_{18}AuClN_6P]^+$ 629.0679 obs. 629.0688; elemental analysis calc. $C_{22}H_{17}AuClN_6P$: C 42.02, H 2.73, N 13.37%, found C 42.25, H 2.69, N 13.52%. Structure corroborated by single crystal X-ray diffraction determination.

Synthesis of chloro[tris(1-phenyl-1*H*-1,2,3-triazol-5-yl)phosphane] gold(I) (**10**)

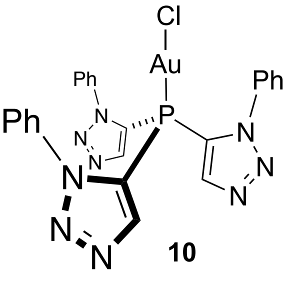

10

Following *PGC General Procedure* on a 0.1 mmol scale. White solid (63 mg, 91%); m.p. 230 (deco); ^{1}H NMR (400 MHz, $CDCl_3$) δ 7.91 (s, 3H, Trz*H*), 7.61 (t, *J* 7.6, 3H), 7.47 (t, *J* 7.94, 6H), 7.62 (d, *J* 7.6, 6H); ^{31}P NMR (121 MHz, $CDCl_3$) δ -44.19; ^{13}C NMR (101 MHz. $CDCl_3$) δ 141.75 (d, $^{2}J_{CP}$ 12.0), 134.45, 132.16, 130.34, 126.22, 124.87 (d, J_{CP} 89.32); IR ν (cm^{-1}) 2923, 2853, 1759, 1496; TOF MS ES+ m/z: 464.11 [M-Au-

Cl+H]$^+$, 696.03 [M+H]$^+$; HR-MS calc. [$C_{24}H_{19}AuClN_9P$]$^+$ 696.0850 obs. 696.0866. Structure corroborated by single crystal X-ray diffraction determination.

Synthesis of chloro[5-(dicyclohexylphosphanyl)-1-(2,6-dimethoxyphenyl)-1H-1,2,3-triazole] gold(I) (**11**)

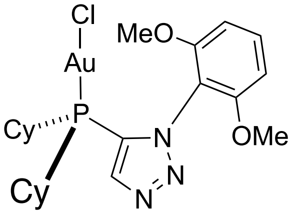

11

Following *PGC General Procedure* on a 0.1 mmol scale. White solid (48 mg, 86%); m.p. 260); ^{1}H NMR (500 MHz, $CDCl_3$) δ 7.97 (s, 1H), 7.60 (t, *J* 8.6, 1H), 6.72(d, *J* 8.6), 3.75 (s, 6H), 2.15-2.07 (m, 2H), 1.94-1.68 (m, 10H), 1.37-1.17 (m, 10H); ^{31}P NMR (121 MHz, $CDCl_3$) δ 22.03; ^{13}C NMR (126 MHz, $CDCl_3$) δ 156.17, 138.23 (d, *J* 7.2), 133.07, 127.11 (d, *J* 58.6), 113.41, 104.78, 55.72, 35.53 (d, *J* 36.4), 29.34 (d, *J* 3.5), 28.53, 26.44 (d, *J* 3.9), 26.33, 25.52 (d, *J* 1.5); IR ν (cm^{-1}); 2928, 2852, 1600, 1483, 1447, 1262; TOF MS ES+ m/z: 634.11 [M+H]$^+$; HR-MS calc. [$C_{22}H_{33}AuClN_3O_2P$]$^+$ 634.1659 obs. 634.1657. Structure corroborated by single crystal X-ray diffraction determination.

Materials prepared for substrate screening

Synthesis of 2-methyl-1-naphthaldehyde (**S2**)

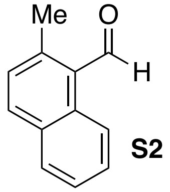

S2

To an oven-dried, two-necked, round bottom flask, 1-bromo-2-methylnaphthalene (4 mL, 26.0 mmol) was dissolved in tetrahydrofuran (50 mL). The mixture was cooled to -78 °C, and *n*-BuLi (2.5 M in hexane, 15.5 mL, 31.0 mmol) was added dropwise. After stirring at that temperature for one hour, *N*,*N*-dimethylformamide (3 mL, 39.0 mmol) was added dropwise and the reaction mixture was allowed to warm to room temperature. Saturated ammonium chloride (30 mL, aqueous) was added and the mixture extracted with dichloromethane (3 × 15 mL). The combined organic fractions were dried over anhydrous magnesium sulphate, filtered, and concentrated *in vacuo* to afford a residue that was purified by flash chromatography (*CombiFlash Rf*, gradient elution hexane:ethyl acetate 1:0 to 0:1). Yellow solid, 3.53 g (80% yield); IR (neat) ν_{max} (cm^{-1}) 3332, 3107, 2880, 2777, 1671, 1593; ^{1}H NMR (300 MHz, $CDCl_3$) δ 10.96 (s, 1H), 8.97 (dd, *J* 8.7 & 0.6, 1H), 7.94 (d, *J* 8.4, 1H), 7.83 (d, *J* 8.6, 1H), 7.62 (ddd, *J* 8.6, 6.9 & 1.5, 1H), 7.50 (ddd, *J* 8.0, 6.9 & 1.1, 1H), 7.34 (d, *J* 8.4, 1H), 2.81 (s, 3H). ^{13}C NMR (101 MHz, $CDCl_3$) δ 193.3, 142.6, 134.3, 132.5, 131.5, 129.8, 128.8, 128.44, 128.41 126.0, 124.4, 20.1. TOF MS EI$^+$ *m/z* 141 [M-CO]$^+$, 170 [M]$^+$. Data are consistent with those described in the literature.[9]

Synthesis of dimethyl-1-diazo-2-oxopropylphosphonate (**S3**)

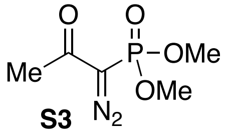

S3

Following a reported procedure,[10] to a cooled solution of dimethyl (2-oxopropyl)phosphonate (970 μL, 5.81 mmol) in anhydrous acetonitrile (6 mL) potassium carbonate (1.044 g, 7.55 mmol) was added, followed by tosyl azide (30% v/v in hexane, 1.26 g, 6.39 mmol) at 0 °C protect from the atmosphere (argon). The reaction mixture was stirred at 0 °C until consumption of the starting material was judged complete (TLC analysis). Diethyl ether (30 mL) was added and the reaction mixture was filtered through a pad of celite and concentrated *in vacuo* to afford a residue that was purified by flash chromatography

(*CombiFlash Rf*, gradient elution hexane:ethyl acetate 1:0 to 0:1). Pale yellow oil, 937 mg (84% yield); IR (neat) ν_{max} (cm^{-1}) 2120, 1655, 1260; ^{1}H NMR (400 MHz, $CDCl_3$) δ 3.85 (d, *J* 11.9, 6H), 2.28 (s, 3H); ^{13}C NMR (101 MHz, $CDCl_3$) δ 189.9 (d, *J* 13.2), 128.0 (d, *J* 325.8), 53.6 (d, *J* 5.5), 27.1 (d, *J* 1.3); ^{31}P NMR (162 MHz, $CDCl_3$) δ 14.3 (sept, *J* 11.9). TOF MS ES^{+} *m/z* 165 [M-2N]$^{+}$. Data are consistent with those described in the literature.[11]

Synthesis of 1-ethynyl-2-methylnaphthalene (**13e**)

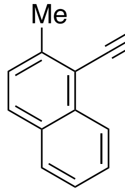

13e

To an oven-dried two-necked flask, 2-methyl-1-naphthaldehyde **S2** (4.30 mmol) and anhydrous methanol (25 mL, anhydrous) were added, under an argon flow. To this solution potassium carbonate (10.75 mmol) was added, followed by dimethyl-1-diazo-2-oxopropylphosphonate **S3** (5.16 mmol). The reaction was stirred at room temperature for ten hours. Diethyl ether (30 mL) was added and the reaction mixture was washed sodium hydrogen carbonate solution (0.1 M, aqueous, 1 x 15 mL) and brine (1 × 15mL). The organic fraction was dried over anhydrous magnesium sulphate, filtered, and concentrated *invacuo* to afford a residue that was purified by flash chromatography (*CombiFlash Rf*, gradient elution hexane:ethyl acetate 1:0 to 0:1). Data are consistent with those described in the literature.[12] Pale pink oil, 520 mg (72% yield); IR (neat) ν_{max} (cm^{-1}) 3291, 3054, 2919, 2098, 1508. ^{1}H NMR (300 MHz, $CDCl_3$) δ 8.35 (d, *J* 8.4, 1H), 7.80 (d, *J* 8.1, 1H), 7.75 (d, *J* 8.3, 1H), 7.6-7.49 (m, 1H), 7.51-7.36 (m, 1H), 7.34 (d, *J* 8.4, 1H), 3.71 (s, 1H), 2.66 (s, 3H). ^{13}C NMR (101 MHz, $CDCl_3$) δ 140.2, 133.91, 131.5, 128.6, 128.1, 128.0, 127.0, 125.8, 125.6, 118.3, 86.4, 80.6, 21.4. TOF MS EI^{+} *m/z* 165 [M-H]$^{+}$, 166 [M]$^{+}$.

Synthesis of 3-methyl-3-(prop-2-yn-1-yl)indolin-2-one (**13j**)[13]

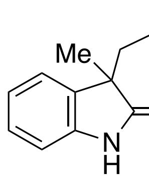

13j

n-Butyl lithium in hexanes (9.0 mL, 18.0 mmol, 2.0 M) was transferred into a nitrogen flushed flask. Anhydrous tetrahydrofuran (20 mL) was added and the solution cooled to -78 °C. A solution of 3-methyl-2-oxindole (2.02 g, 13.8 mmol) dissolved in tetrahydrofuran (20 mL) was added dropwise over 10 min to the stirred solution. The reaction mixture was stirred for a further 10 min before propargyl bromide (1.40 mL, 15.7 mmol) was added. The solution was allowed to warm to room temperature and stirred under a nitrogen atmosphere for 24 h. Methanol (20 mL) was added to quench any residual organometallic reagent. The solution was concentrated *in vacuo* and the residual oil was resuspended in ethyl acetate (50 mL) and washed with water (2 × 100 mL), brine (2 × 100 mL), dried over anhydrous magnesium sulphate, filtered and evaporated to dryness. The crude residue was purified by flash column chromatography (silica, petroleum ether:ethylacetate / 4:1) to afford the desired title compound 3-methyl-3-(prop-2-yn-1-yl)indolin-2-one (0.85 g, 33%, **13j**) as a cream powdery solid. Compound **13j** was synthesised multiple times during the course of this study in similar yields. The major product was however the doubly alkynylated 3-methyl-1,3-di(prop-2-yn-1-yl)indolin-2-one (1.33 g, **S4**) as a dark yellow-brown viscous oil, this material was retained for use in another study, and preliminary analysis given below. TLC R_f = 0.60 (silica, petroleum ether:ethylacetate / 1:1); ^{1}H NMR (400 MHz, $CDCl_3$): δ 9.14 (1H, brs, NH), 7.42 (1H, d, *J* 8.0, C_{Ar}*H*), 7.24 (1H, app td, *J* 8.0 & 1.2, C_{Ar}*H*), 7.06 (1H, app td, *J* 7.6 & 0.8, C_{Ar}*H*), 6.97 (1H, d, *J* 8.0, C_{Ar}*H*), 2.71 (1H, dd, *J* 16.4 & 2.8, CH*H*CCH), 2.56 (1H, dd, *J* 16.4 & 2.4, C*H*HCCH), 1.98 (1H,

t, J 2.4, CCH), 1.49 (3H, s, CH_3);. ^{13}C NMR (100 MHz, $CDCl_3$): δ 182.1, 140.2, 133.4, 128.2, 123.5, 122.5, 110.0, 79.5, 70.8, 47.2, 27.5, 21.9. Data are consistent with those described in the literature.[13]

Analysis of 3-methyl-1,3-di(prop-2-yn-1-yl)indolin-2-one (**S4**) obtained as described above [13]

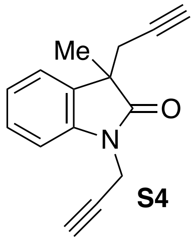
S4

Dark yellow-brown viscous oil; TLC R_f = 0.88 (silica, petroleum ether:ethylacetate / 1:1); ^{1}H NMR (400 MHz, $CDCl_3$): δ, 7.47 (1H, dt, J 7.4 & 0.7, $C_{Ar}H$), 7.34 (1H, app td, J 7.8 & 1.3, $C_{Ar}H$), 7.13 (1H, app td, J 7.6 & 1.0, $C_{Ar}H$), 7.10 (1H, d, J 7.8, $C_{Ar}H$), 4.53 (2H, d, J 2.5, CH_2CCH), 2.71 (1H, dd, J 16.6 & 2.7, CHHCCH), 2.53 (1H, dd, J 16.5 & 2.6, CHHCCH), 2.25 (1H, t, J 2.5, $CCH_{(b)}$), 1.98 (1H, t, J 2.6, $CCH_{(a)}$), 1.48 (3H, s, CH_3); ^{13}C NMR (100 MHz, $CDCl_3$): 178.4, 141.1, 132.8, 128.3, 123.3, 123.0, 109.1, 79.4, 77.0, 72.4, 71.0, 46.6, 29.3, 27.7, 21.8. Data are consistent with those described in the literature.[13]

Synthesis of 1-benzyl-3-methyl-3-(prop-2-yn-1-yl)indolin-2-one (**13k**)[13]

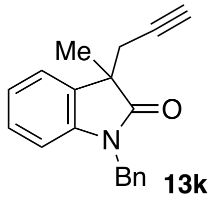
13k

Sodium hydride (60% dispersion) (275 mg, 11.4 mmol) was stirred in anhydrous tetrahydrofuran (50 mL) under a nitrogen atmosphere. The reaction mixture was cooled to 0 °C (ice slush-bath) and stirred vigorously while a solution of **13j** (752 mg, 4.06 mmol) in tetrahydrofuran (10 mL) was added dropwise. The resulting mixture was stirred until the visible formation of gas had ceased (~30 min), after which benzyl bromide (0.60 mL, 5.04 mmol) was added. The reaction mixture was allowed to warm to room temperature and left to stir for two hours. Water (10 mL) was added dropwise to decompose any remaining sodium hydride and the resulting solution was extracted with ethyl acetate (3 × 50 mL). The combined organic phase was washed with brine (3 × 50 mL), dried over anhydrous magnesium sulphate, filtered and concentrated *in vacuo*. The residue thus obtained was purified by flash column chromatography (silica, petroleum ether:ethylacetate / 4:1) and recrystallised from petroleum ether and ethyl acetate to afford the title compound as a pale yellow crystalline solid (799 mg, 71%). TLC R_f = 0.50 (silica, petroleum ether:ethylacetate / 4:1); ^{1}H NMR (400 MHz, $CDCl_3$): δ 7.37 (1H, d, J 7.6, $C_{Ar}H$), 7.20-7.26 (5H, m, $C_{Ar}H$), 7.13 (1H, app td, J 7.6 & 1.2, $C_{Ar}H$), 7.00 (1H, app td, J 7.6 & 0.8, $C_{Ar}H$), 6.68 (1H, d, J 7.6, $C_{Ar}H$), 5.01 (d, 1H, J 15.6 CHHPh), 4.83 (d, 1H, J 15.6 CHHPh), 2.75 dd, 1H, J 16.4 & 2.8, CHHCCH), 2.60 (dd, 1H, J 16.4 & 2.8, CHHCCH), 1.87 (1H, t, J 2.4, CCH), 1.46 (3H, s, CH_3); ^{13}C NMR (100 MHz, $CDCl_3$): δ 179.4, 142.1, 135.8, 132.9, 128.7, 128.1, 127.6, 127.3, 123.2, 122.6, 109.1, 79.7, 70.7, 46.7, 43.7, 27.7, 22.3. Data are consistent with those described in the literature.[13]

Synthesis of *tert*-butyl 3-methyl-2-oxo-3-(prop-2-yn-1-yl)indoline-1-carboxylate (**13l**)[13]

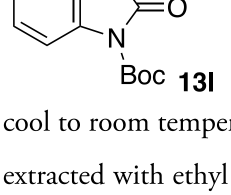
13l

To a stirred solution of **13j** (1.02 g, 5.53 mmol) in anhydrous tetrahydrofuran (50 mL) anhydrous sodium carbonate (4.75 g, 44.8 mmol) and di-*tert*-butyl dicarbonate (3.01 g, 13.8 mmol) were added, under a nitrogen atmosphere. The reaction mixture was heated at reflux starting material was consumed (as just by TLC analysis). The resulting mixture was allowed to cool to room temperature and concentrated *in vacuo*. The residue thus obtained was suspended in water (50 mL) and extracted with ethyl acetate (3 × 50 mL). The combined organic extracts were washed with brine (2 × 50 mL), dried

over anhydrous magnesium sulphate, filtered and solvent remove *in vacuo*. The residue obtained was purified by flash column chromatography (silica, petroleum ether:ethylacetate / 5:1) to the title compound as a pale yellow viscous oil (2.28 g, quant.) TLC R_f = 0.51 (silica, petroleum ether:ethylacetate / 6:1); ^{1}H NMR (400 MHz, $CDCl_3$): δ 7.86 (1H, d, *J* 8.0, $C_{Ar}H$), 7.44 (1H, dt, *J* 7.6 & 0.8, $C_{Ar}H$), 7.32 (1H, app td, *J* 7.6 & 1.2, $C_{Ar}H$), 7.18 (1H, app td, *J* 7.6 & 1.2, ArH), 2.70 (1H, dd, *J* 16.4 & 2.8, CH*H*CCH), 2.57 (1H, dd, *J* 16.8 & 2.8, C*H*HCCH), 1.99 (1H, t, *J* 2.8, CC*H*), 1.64 (9H, s, *t*-Bu), 1.49 (3H, s, CH_3); ^{13}C NMR (100 MHz, $CDCl_3$): δ 177.9, 149.2, 138.8, 131.7, 128.4, 124.4, 123.0, 114.9, 84.4, 79.0, 71.3, 47.0, 28.4, 28.0, 22.8. Data are consistent with those described in the literature.[13]

Analysis of materials resulting from substrate screening in catalysis

Analysis of decan-2-one (**14a**)[14]

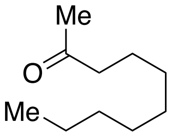

14a

Colourless oil; ^{1}H NMR (400 MHz, $CDCl_3$) δ 2.41 (t, *J* 7.5, 2H), 2.13 (s, 3H), 1.62-1.52 (m, 2H), 1.28 (brs, 10H), 0.88 (t, *J* 6.9, 3H); ^{13}C NMR (101 MHz, $CDCl_3$) δ 209.43[-], 43.84[-], 31.82[-], 29.86[+], 29.37[-], 29.20[-], 29.14[-], 23.89[-], 22.65[-], 14.10[+]; IR ν (cm^{-1}) 2924, 2854, 1716, 1464, 1358, 1162; TOF MS EI+ m/z: 156.2 $[M]^+$. Data are consistent with those described in the literature.[14]

Analysis of 1-cyclohexylethan-1-one (**14b**)

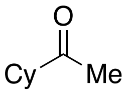

14b

Colourless oil; ^{1}H NMR (400 MHz, $CDCl_3$) δ 2.37-2.28 (m, 1H), 2.13 (s, 3H, C*H*$_3$), 1.93-1.58 (m, 5H), 1.40-1.13 (m,5H); ^{13}C NMR (101 MHz, $CDCl_3$) δ 212.15[-], 51.38[+], 28.39[-], 27.80[+], 25.82[-], 25.59[-]; IR ν (cm^{-1}) 2927, 285, 1705, 1448, 1351, 1166; TOF MS EI+ m/z: 126.1 $[M]^+$.

Analysis of 1-(cyclohex-1-en-1-yl)ethan-1-one (**14c**)

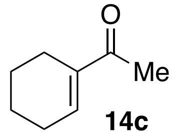

14c

Yellowlish oil; ^{1}H NMR (400 MHz, $CDCl_3$) δ 6.95-6.86 (m, 1H), 2.30-2.18 (m, 7H), 1.68-1.57 (m, 4H); ^{13}C NMR (101 MHz, $CDCl_3$) δ 199.30[-], 140.89[+], 139.63[-], 26.07[-], 25.12[+], 22.92[-], 21.89[-], 21.50[-] ; IR ν (cm^{-1}) 2932, 2860, 1662, 1432, 1224; TOF MS EI+ m/z: 124.1 $[M]^+$.

Analysis of acetophenone (**14d**)[14]

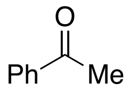

14d

Pale yellow oil; ^{1}H NMR (400 MHz, $CDCl_3$) δ 7.99 (dd, *J* 8.4, 1.3, 2H, Ar*H*), 7.56 (tt, *J* 7.3, 1.3, 1H, Ar*H*), 7.45 (t, *J* 7.3, 2H, Ar*H*), 2.59 (s, 3H, C*H*$_3$); ^{13}C NMR (101 MHz, $CDCl_3$) δ 198.12[-], 137.13[-], 133.10[+], 128.57[+], 128.30[+], 26.60[+]; IR ν (cm^{-1}) 1680, 1598, 1448, 1358, 1263; TOF MS EI+ m/z: 120.1 $[M]^+$. Data are consistent with those described in the literature.[14]

Analysis of 1-(2-methylnaphthalen-1-yl)ethan-1-one (**14e**)

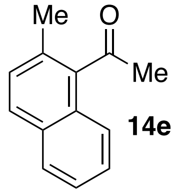

14e

Colourless oil; ^{1}H NMR (400 MHz, $CDCl_3$) δ 7.82 (d, *J* 7.5, 1H), 7.76 (d, *J* 8.5, 1H), 7.60 (d, *J* 8.5, 1H), 7.50-7.42 (m, 2H), 7.29 (d, *J* 8.4, 1H), 2.62 (s, 3H), 2.43 (s, 3H); ^{13}C NMR (101 MHz, $CDCl_3$) δ 208.25[-], 138.78[-], 131.76[-], 129.94[-], 128.90[-], 128.77[+], 128.60[+], 128.29[+], 126.91[+], 125.48[+], 123.91[+], 32.93[+], 19.39[+]; IR ν (cm^{-1}) 1697, 1508, 1419, 1350, 1209; TOF MS EI+ m/z: 184.1 $[M]^+$, 169.1 $[M-CH_3]^+$.

Analysis of 4-phenylbutan-2-one (**14f**)[15]

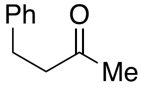

14f

Colourless oil; ^{1}H NMR (400 MHz, $CDCl_3$) δ 7.32-7.25 (m, 2H) 7.23-7.16 (m, 3H), 2.90 (t, *J* 7.7, 2H), 2.76 (t, *J* 7.5, 2H), 2.14 (s, 3H, C*H₃*); ^{13}C NMR (101 MHz, $CDCl_3$) δ 207.9[-], 141.0[-], 128.5[+], 128.3[+], 126.1[+], 45.2[-], 30.1[+], 29.8[-]; IR ν (cm^{-1}) 2924, 1714, 1496, 1357, 1161; TOF MS EI+ m/z: 148.1 $[M]^+$.

Analysis of 1-(4-methoxyphenyl)ethan-1-one (**14g**)[14]

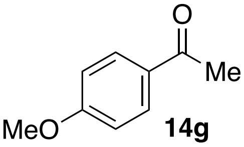

14g

Yellow solid(literature reported as yellow oil); ^{1}H NMR (400 MHz, $CDCl_3$) δ 7.92 (d, *J* 9.0, 2H, Ar*H*), 6.91 (d, *J* 8.9, 2H, Ar*H*), 3.84 (s, 3H, OCH_3), 2.53 (s, 3H, CH_3); ^{13}C NMR (101 MHz, $CDCl_3$) δ 196.7[-], 163.4[-], 130.5[+], 130.2[-], 113.6[+], 55.4[+], 26.3[+]; IR ν (cm^{-1}) 1671, 1597, 1509, 1459, 1417, 1356, 1247, 1169; TOF MS EI+ m/z: 150.1 $[M]^+$.

Analysis of 1-(4-(4,4,5,5-tetramethyl-1,3,2-dioxaborolan-2-yl)phenyl)ethan-1-one (**14h**)

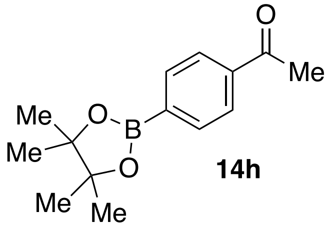

14h

White solid; ^{1}H NMR (400 MHz, $CDCl_3$) δ 7.93 (d, *J* 8.4, Ar*H*), 7.89 (d, *J* 8.4, Ar*H*), 2.61 (s, 3H, CH_3), 1.36 (s, 12H, C(CH_3)$_2$); ^{13}C NMR (101 MHz, $CDCl_3$) δ 198.5[-], 139.0[-], 134.9[+], 127.3[+], 84.2[-], 26.8[+], 24.9[+]; IR ν (cm^{-1}) 2979, 1686, 1507, 1397, 1356, 1265; TOF MS EI+ m/z: 246.2 $[M]^+$.

Analysis of 1-(thiophen-3-yl)ethan-1-one (**14i**)

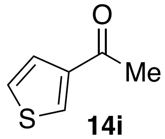

14i

White solid; ^{1}H NMR (400 MHz, $CDCl_3$) δ 8.31 (dd, *J* 2.9, 1.3, 1H), 7.54 (dd, *J* 5.1, 1.3, 1H), 7.31 (dd, *J* 5.1, 2.9), 2.53 (s, 3H, CH_3); ^{13}C NMR (101 MHz, $CDCl_3$) δ 192.3[-], 142.6[-], 132.4[+], 132.4[+], 127.0[+], 126.4[+], 27.6[+]; IR ν (cm^{-1}) 3103, 1667, 1510, 1410, 1352, 1254; TOF MS ES+ m/z: 126.0 $[M]^+$.

Analysis of 3-methyl-3-(2-oxopropyl)indolin-2-one (**14j**)

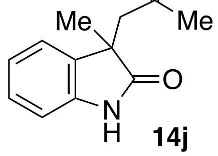

14j

White solid; m.p. 150-152 °C; ^{1}H NMR (400 MHz, $CDCl_3$) δ 7.96 (brs, 1H, N*H*), 7.18 (td, *J* 7.7, 1.3, 1H, Ar*H*), 7.12 (dt, *J* 7.4, 0.6, 1H, Ar*H*), 6.99 (td, *J* 7.5, 1.0, 1H, Ar*H*), 6.90 (d, *J* 7.7, 1H, Ar*H*), 3.12 (ABq, *J* 17.8, 2H, CH_2), 2.02 (s, 3H, COCH_3), 1.35 (s, 3H, CCH_3); ^{13}C NMR (101 MHz, $CDCl_3$) δ 204.7[-], 182.1[-], 140.7[-], 133.9[-], 127.9[+], 122.3[+], 122.2[+], 109.9[+], 50.4[-], 45.6[-], 30.0[+], 24.6[+]; IR ν (cm^{-1}) 1709, 1620, 1472, 1196; TOF MS EI+ m/z: 203.1 $[M]^+$; HRMS calc. $[C_{12}H_{13}NO_2]^+$ 203.0940, obs. 203.0945.

Analysis of 1-benzyl-3-methyl-3-(2-oxopropyl)indolin-2-one (**14k**)

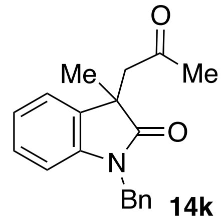

14k

White solid; m.p. 115-117 °C; ^{1}H NMR (400 MHz, $CDCl_3$) δ 7.39 (d, *J* 7.6, 2H), 7.33 (t, *J* 7.3, 2H), 7.24(t, *J* 7.1, 1H), 7.15-7.08 (m, 2H), 6.96 (td, *J* 7.5, 1.0, 1H), 6.69, (d, *J* 7.8, 1H), 4.97 (ABq, *J* 15.9, 2H, PhC*H*$_2$), 3.16 (ABq, *J* 17.8, 2H, C*H*$_2$CO), 2.01 (s, 3H, COC*H*$_3$), 1.39 (s, 3H, CC*H*$_3$); ^{13}C NMR (101 MHz, $CDCl_3$) δ 204.5[-], 180.4[-], 142.8[-], 136.2[-], 133.5[-], 128.7[+], 127.8[+], 127.4[+], 127.3[+], 122.3[+], 121.8[+], 109.4[+], 50.4[-], 45.3[-], 44.0[-], 30.0[+], 25.1[+]; IR ν (cm^{-1}) 1704, 1611, 1489, 1355, 1176; TOF MS EI+ m/z: 293.2 $[M]^+$; HRMS calc. $[C_{19}H_{19}NO_2]^+$ 293.1410, obs. 293.1417.

Analysis of *tert*-butyl 3-methyl-2-oxo-3-(2-oxopropyl)indoline-1-carboxylate (**14l**)

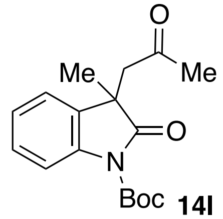

14l

White solid; m.p. 120-121 °C; ^{1}H NMR (400 MHz, $CDCl_3$) δ 7.88 (d, *J* 8.2, 1H), 7.30-7.24 (m, 1H), 7.13-7.08 (m, 2H), 3.17 (ABq, *J* 17.8, 2H), 2.00 (s, 3H, COC*H*$_3$), 1.66 (s, 9H, C(C*H*$_3$)$_3$), 1.36 (s, 3H, CC*H*$_3$);^{13}C NMR (101 MHz, $CDCl_3$) δ 204.2[-], 178.9[-], 149.5[-], 139.7[-], 132.4[-], 128.1[+], 124.2[+], 121.3[+], 115.4[+], 84.1 [-], 51.6[-], 45.5[-], 29.7[+], 28.2[+], 25.6[+]; IR ν (cm^{-1}) 1764, 1717, 1601, 1480, 1297, 1148. TOF MS ES+ m/z: 326.1 $[M+Na]^+$; HRMS calc. $[C_{17}H_{21}NO_4Na]^+$ 326.1363, obs. 326.1368.

Analysis of 3,3-dimethoxy-1-phenylpropan-1-one (**14m'**)

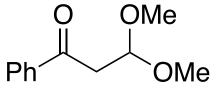

14m'

Light yellow oil; ^{1}H NMR (400 MHz, $CDCl_3$) δ 7.95 (d, *J* 8.5, 2H), 7.59 (tt, *J* 7.3, 1.3, 1H), 7.46 (t, *J* 7.6, 2H), 5.01 (t, *J* 5.5, 1H, CH_2C*H*), 3.41 (s, 6H, OC*H*$_3$), 3.28 (d, *J* 5.5, 2H, C*H*$_2$CH); ^{13}C NMR (101 MHz, $CDCl_3$) δ 196.9[-], 137.1[-], 133.3[+], 128.6[+], 128.3[+], 102.2[+], 54.2[+], 42.6[-]; IR ν (cm^{-1}) 2935, 1683, 1597, 1448, 1186, 1118; TOF MS EI+ m/z: 194.1 $[M]^+$, 179.1 $[M-CH_3]^+$.

Materials synthesised for or arising from regioselectivity study

Synthesis of but-1-yne-1,4-diyldibenzene (**15**)

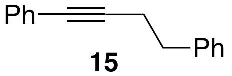

15

A dry flask was charged with bis(triphenylphosphine)palladium(II) dichloride (84.0 mg, 0.02 mmol), copper(I) iodide (45.0 mg, 0.24 mmol), tetrahydrofuran (30 mL, degassed), iodobenzene (0.67 mL, 6.01 mmol) triethylamine (5.8 mL, 41.6 mmol, degassed) and 4-phenyl-1-butyne (0.93 mL, 6.61 mmol) sequentially and stirred at room temperature for 20 h. The solvent was removed *in vacuo* and the resulting slurry was filtered through a pad of silica (3 cm) eluting with diethylether. The eluted filtrate was concentrated *in vacuo* and purified by flash chromatography (silica, hexane) to give alkyne **15** as a colourless oil (1.23 g, 99%); IR ν (cm^{-1}) 3027, 2927, 1599, 1490; ^{1}H NMR (400 MHz, $CDCl_3$): δ 7.39-7.19 (m, 10H), 2.92 (t, *J* 7.5, 2H), 2.69 (t, *J* 7.5, 2H); ^{13}C NMR (101 MHz, $CDCl_3$): δ 140.9[-], 131.7[+], 128.7[+], 128.5[+], 128.3[+], 127.8[+], 126.5[+], 124.0[+], 89.6[-], 81.5[-], 35.3[-], 21.8[-]. Spectroscopic data are consistent with those reported.[16]

Procedure resulting in mixtures of **16a** and **16b**

Following a method adapted from a literature procedure,[17] an 8 mL vial equipped with a screw cap and magnetic stirrer bar was charged with alkyne **15** (103 mg, 0.50 mmol) and purged with argon for 2 minutes. Gold(I) chloride complex (1 mol %), silver(I) triflate (2 mol %), methanol (0.5 mL, degassed) and water (18 µL, 1.0 mmol, degassed) were sequentially added and the sealed reaction vessel heated at 80 °C for 14 h. After allowing to cool to room temperature, the reaction mixture was filtered through a pad of Celite (2 cm eluted with dichloromethane) and concentrated *in vacuo*. The conversion of **15** into products **16a** and **16b** was analysed and the ratio thereof determined. For proton NMR spectroscopic analysis methyl 3,5-dinitrobenzoate was added as an internal standard (4.09 ppm, $CDCl_3$), integration of **16a** measured from quintet resonance at 2.09 ppm and integration of **16b** measured from benzylic methylene at 3.69 ppm. In some cases overlapping signals in the proton NMR spectrum prevented accurate determination of the proportion of the minor regioisomer (see Supplementary Figure S 68, Supplementary Figure S 69 (corresponding to Table 2, Entry 1, main text) and Supplementary Figure S 70 (corresponding to Table 2, Entry 2, main text)) and gas chromatography was used to confirm conversion to and ratios of **16a** and **16b** in those cases. Furthermore, isolated yields (flash chromatography) were used to determine yield and selectivity for use of **8a** and **9a** as precatalysts.

Analysis of 1,4-diphenylbutan-1-one (**16a**)

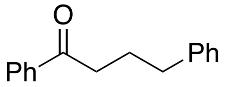

16a

White solid; IR ν (cm^{-1}) 3062, 3027, 2936, 1683, 1598 1448; ^1H NMR (300 MHz, $CDCl_3$): δ 7.97-7.89 (m, 2H), 7.59-7.52 (m, 1H), 7.49-7.41 (m, 2H), 7.34-7.26 (m, 2H), 7.25-7.17 (m, 3H), 2.99 (t, *J* 7.3, 2H), 2.73 (t, *J* 7.5, 2H), 2.09 (app. quint, *J* 7.4, 2H); ^{13}C NMR (101 MHz, $CDCl_3$): δ 200.3, 141.8, 137.1, 133.1, 128.7, 128.7, 128.5, 128.2, 126.1, 37.8, 35.3, 25.8. Data are consistent with those described in the literature.[18]

Analysis of 1,4-diphenylbutan-2-one (**16b**)

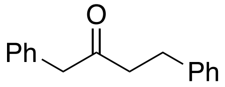

16b

Pale yellow oil; IR ν (cm^{-1}) 6063, 3028, 2926, 1710, 1496, 1454; ^1H NMR (300 MHz, $CDCl_3$): δ 7.37-7.22 (m, 5H), 7.22-7.08 (m, 5H), 3.67 (s, 2H), 2.87 (m, 2H), 2.77 (m, 2H); ^{13}C NMR (101 MHz, $CDCl_3$): δ 207.6, 141.0, 134.2, 129.5, 128.9, 128.6, 128.5, 127.2, 126.2, 50.5, 43.6, 29.9. Data are consistent with those described in the literature.[19]

Preliminary palladium-catalysed cross-coupling findings

Replicating a procedure previously employed by some of the co-authors of this report in an early disclosure of mono-triazole-containing phosphine ligands (and in which ligand **1a**, **3a** and **3b** were reported)[7, 20] the range of multi-triazole phosphines of this study were compared (alongside the three aforementioned ligands) in the **S7**-forming Suzuki-Miyaura cross-coupling reaction shown in Supplementary Figure S 1.

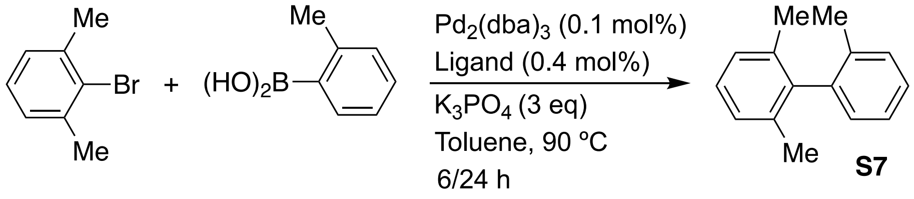

Entry	Ligand	Conversion after 6 h	Conversion after 24 h
1	**1a**	> 99%	> 99%
2	**3a**	> 99%	> 99%
3	**4a**	80%	80%
4	**5**	0 %	0%
5	**4b**	44%	68%
6	**3b**	56%	63%

Supplementary Figure S 1*. Suzuki-Miyaura palladium-catalysed cross-coupling of 2-bromo-1,3-dimethylbenzene with* o*-tolylboronic acid mediated by triazole-containing ligands.*

Disappointingly, the 'bulkiest' of the ligands tested (**5**) failed to convert any of the starting material to product **S7**, indeed decreasing the triazole number (as witnessed for the gold(I)-catalysed hydration of the main text) led to increased levels of product formation (**1a** and **3a** giving quantitative conversion to product **S7** within six hours). In the preliminary survey it may be concluded that mono-triazole-phosphines (of the probed series) are the most effective ligand constructs for palladium-catalysed Suzuki-Miyaura reactions of sterically encumbered aryl-bromides.

At the same time as probing the above reactions (Supplementary Figure S 1) it was deemed of interest to also investigate C-N cross coupling with the same ligand set. In a first test (details not shown) with the same ligand set, it was ligand **1a** that showed the most promise for palladium-catalysed C-N cross-coupling (of *p*-chloro-toluene with *N*-methyl-aniline). Since palladium-catalysed C-N cross-coupling with triazole containing ligands (of type **1**) had not previously been deployed as ligands in such reactions a short survey of preliminary activity (of **1a** as a ligand) was embarked upon, details are shown in Supplementary Figure S 2. Four different C-N cross-coupled products were synthesised but yields were not overwhelmingly promising. Further optimisation will be needed to translate the effectiveness of the **1**-type

ligands from C-C cross-coupling to C-N coupling and is beyond the scope of the current study. Experimental details of the four cross-coupled products (**S8a**-**d**) are given below and in later sections.

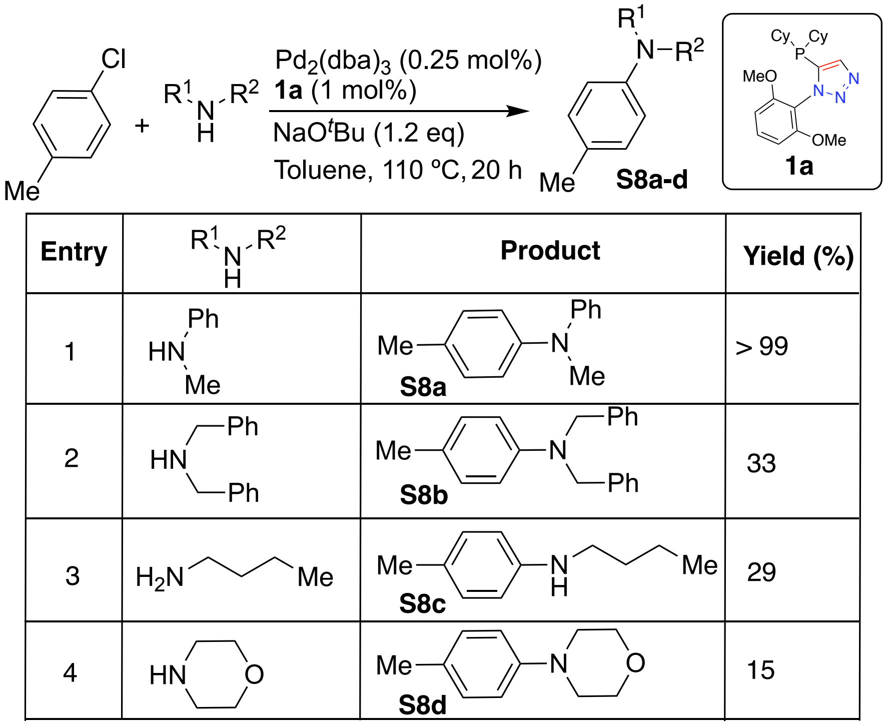

*Supplementary Figure S 2. Palladium-catalysed C-N cross-coupling facilitated by triazole-containing ligand **1a**.*

General procedure for palladium-catalysed C-N cross-coupling

To a 16 mL Kimble tube sodium tertiary butoxide (1.2 equiv.), *p*-chloro-toluene (1.0 equiv.), $Pd_2(dba)_3$ (0.25 mol%), and ligand **1a** (1 mol%) were added. The mixture was dissolved in toluene (3 mL) and the flask flushed with argon. The reaction vessel was placed into an ADS19 OCTO reaction station and heated at 110 °C for 20 hours. After which the vessel was cooled to room temperature and the mixture diluted with ethyl acetate (10 mL) and was washed with brine (6 mL). The aqueous layer was extracted further with ethyl acetate (2 × 10 mL). The combine organic fractions were dried with sodium sulphate and solvent evaporated *in vacuo*. The residues thus obtained were purified by flash chromatography (*Isco CombiFlash*, silica 50-60 µm, 4 or 12 g column, hexane (100%) to hexane (80%): ethyl acetate (20%) gradient elution, detection 254 and 288 nm absorption) to afford the corresponding products.

Synthesis of *N*,4-dimethyl-*N*-phenylaniline **S8a**

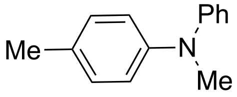

S8a

Pale Yellow liquid, >99% isolated yield; ^{1}H NMR (300 MHz, $CDCl_3$) δ 7.28-7.17 (m, 2H), 7.16-7.07 (m, 2H), 7.04-6.95 (m, 2H), 6.96-6.82 (m, 3H), 3.28 (s, 3H), 2.32 (s, 3H). ^{13}C NMR (101 MHz, $CDCl_3$) δ 149.37, 146.60, 132.07, 129.93, 129.03, 122.59, 119.77, 118.17, 40.34, 20.77. TOF MS ES+ m/z: 198.12 $[M+H]^+$. Data are consistent with those described in the literature.[21]

Synthesis of *N*,*N*-dibenzyl-4-methylaniline **S8b**

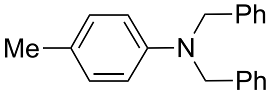

S8b

Colourless liquid, 33% isolated yield, ^1H NMR (300 MHz, $CDCl_3$) δ 7.38-7.18 (m, 10H), 6.98 (d, *J* 8.2, 2H), 6.65 (d, *J* 8.7, 2H), 4.62 (s, 4H), 2.22 (s, 3H). ^{13}C NMR (101 MHz, $CDCl_3$) δ 147.05, 138.87, 129.74, 128.60, 126.81, 126.70, 125.85, 112.63, 54.38, 20.22. TOF MS ES+ m/z: 288.17 $[M+H]^+$. Data are consistent with those described in the literature.[21]

Synthesis of *N*-butyl-4-methylaniline **S8c**

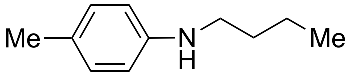

S8c

Colourless liquid, 29% isolated yield; ^1H NMR (300 MHz, $CDCl_3$) δ 7.02-6.93 (m, 2H), 6.58-6.47 (m, 2H), 3.43 (s, 1H), 3.08 (t, *J* 7.1, 2H), 2.23 (s, 3H), 1.65-1.52 (m, 2H), 1.49-1.31 (m, 2H), 0.95 (t, *J* 7.3, 3H). ^{13}C NMR (101 MHz, $CDCl_3$) δ 146.36, 129.74, 126.30, 120.85, 112.93, 44.09, 31.77, 20.36, 13.98. TOF MS ES+ m/z: 164.13 $[M+H]^+$. Data are consistent with those described in the literature.[21]

Synthesis of 4-(*p*-tolyl)morpholine **S8d**

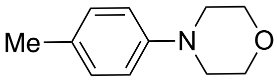

S8d

White crystalline solid, 15% isolated yield; ^1H NMR (300 MHz, $CDCl_3$) δ 7.09 (d, *J* 8.1, 2H), 6.83 (d, *J* 8.6, 2H), 3.88-3.83 (m, 4H), 3.19-2.95 (m, 4H), 2.27 (s, 3H). ^{13}C NMR (101 MHz, $CDCl_3$) δ 149.23, 129.74, 129.59, 116.06, 67.01, 49.95, 20.46. TOF MS ES+ m/z: 178.12 $[M+H]^+$. Data are consistent with those described in the literature.[21]

NMR Spectrums

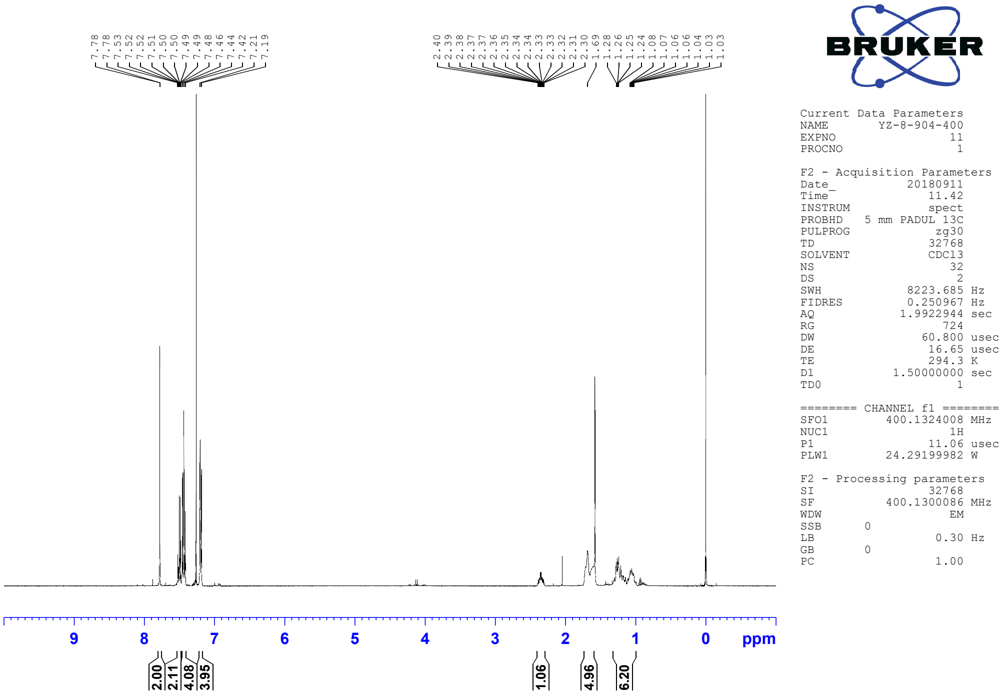

Supplementary Figure S 3. *¹H NMR spectrum of **4a**.*

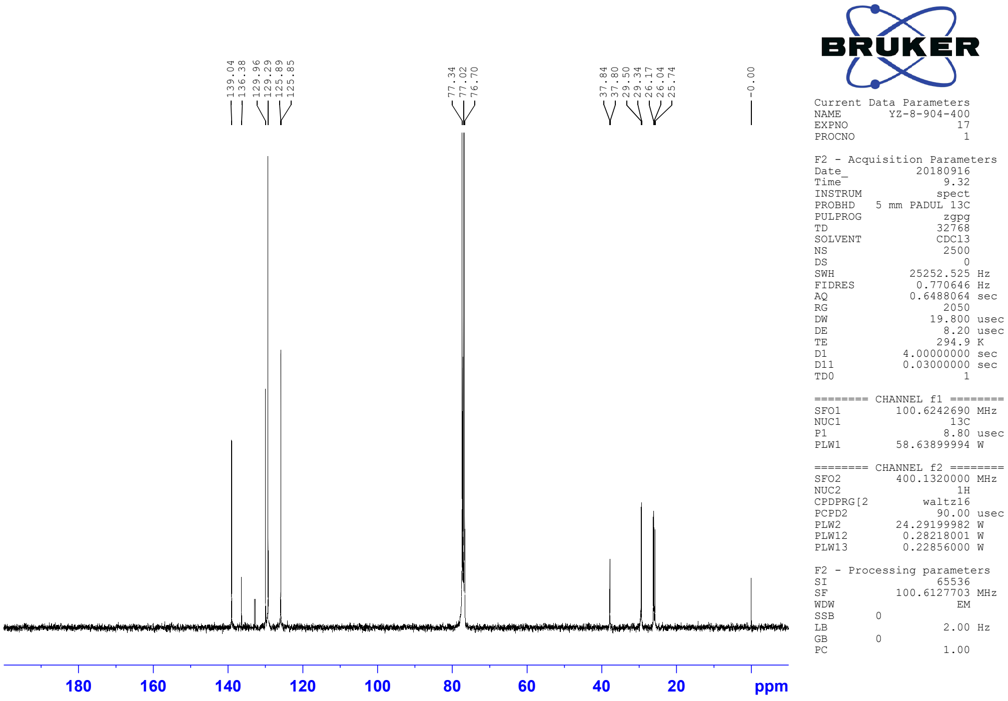

Supplementary Figure S 4. *¹³C NMR spectrum of **4a**.*

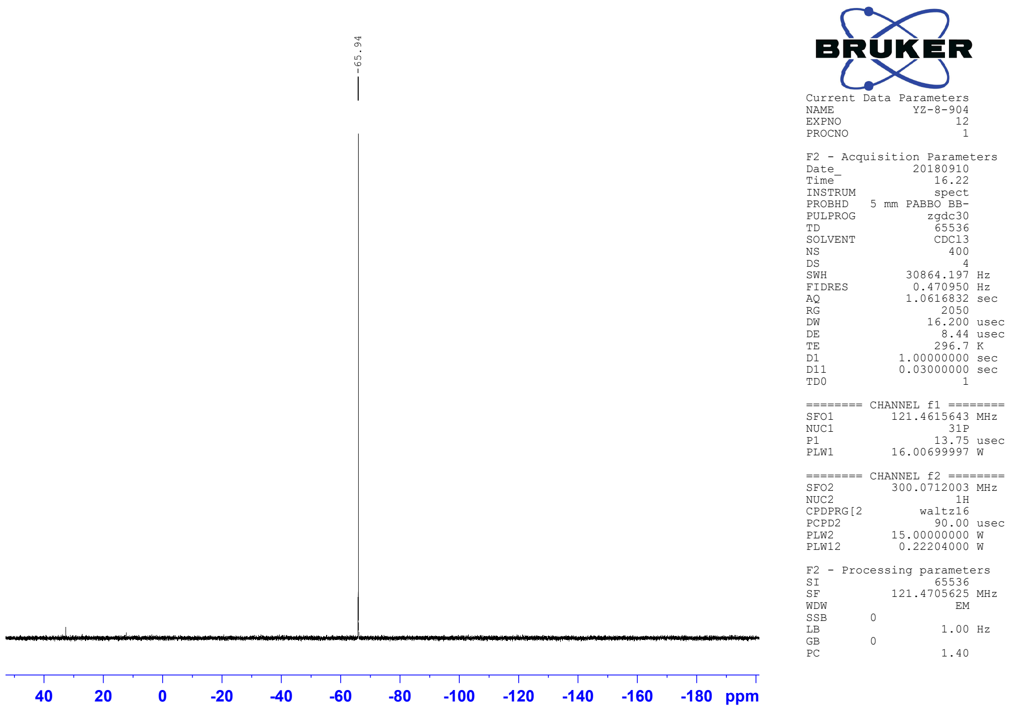

Supplementary Figure S 5. *^{31}P NMR spectrum of* ***4a***.

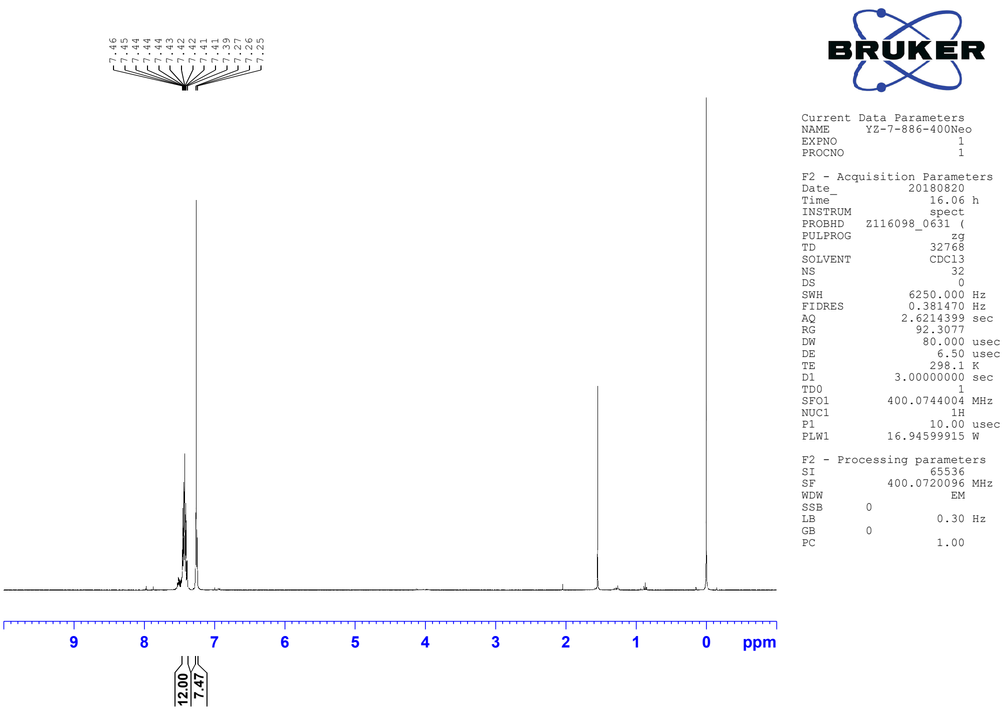

Supplementary Figure S 6. *^{1}H NMR spectrum of **4b**.*

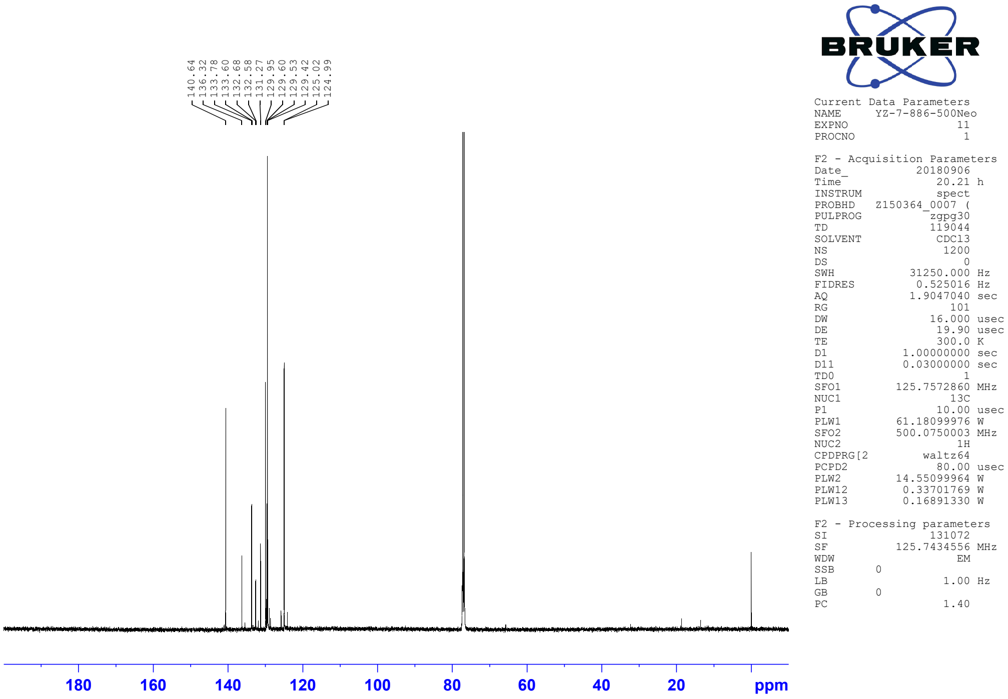

Supplementary Figure S 7. *^{13}C NMR spectrum of **4b**.*

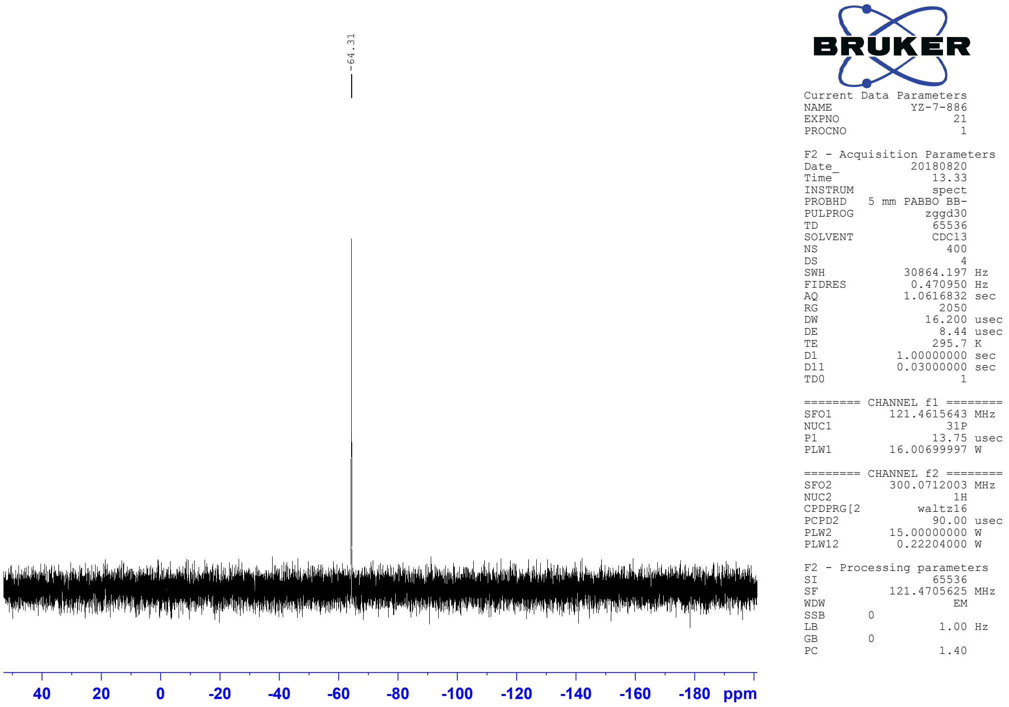

Supplementary Figure S 8. *^{31}P NMR spectrum of* ***4b***.

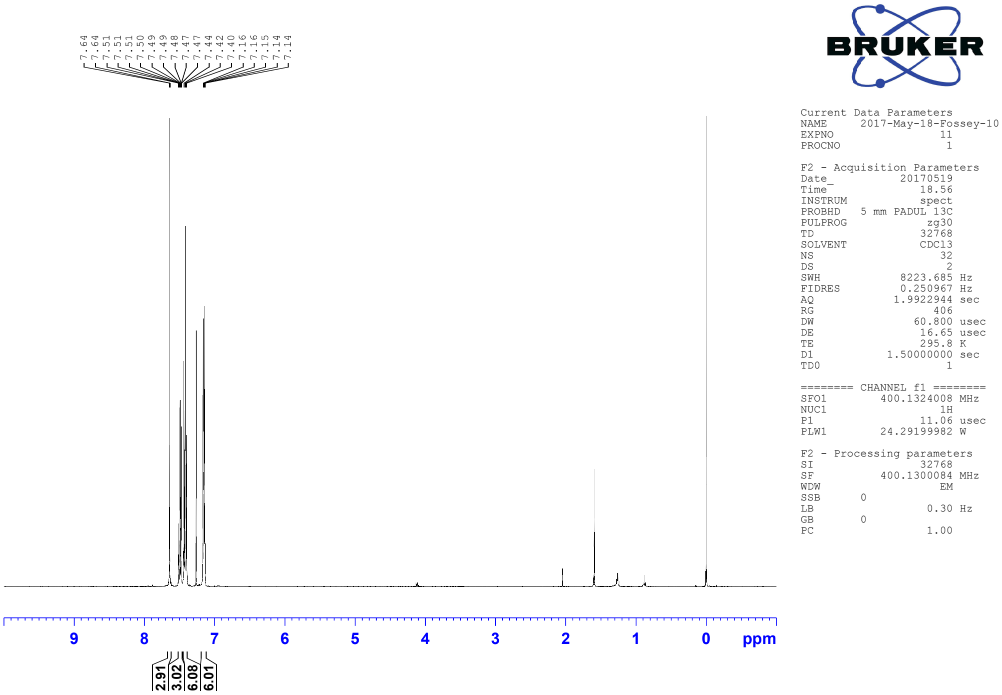

Supplementary Figure S 9. [1]*H NMR spectrum of* ***5a****.*

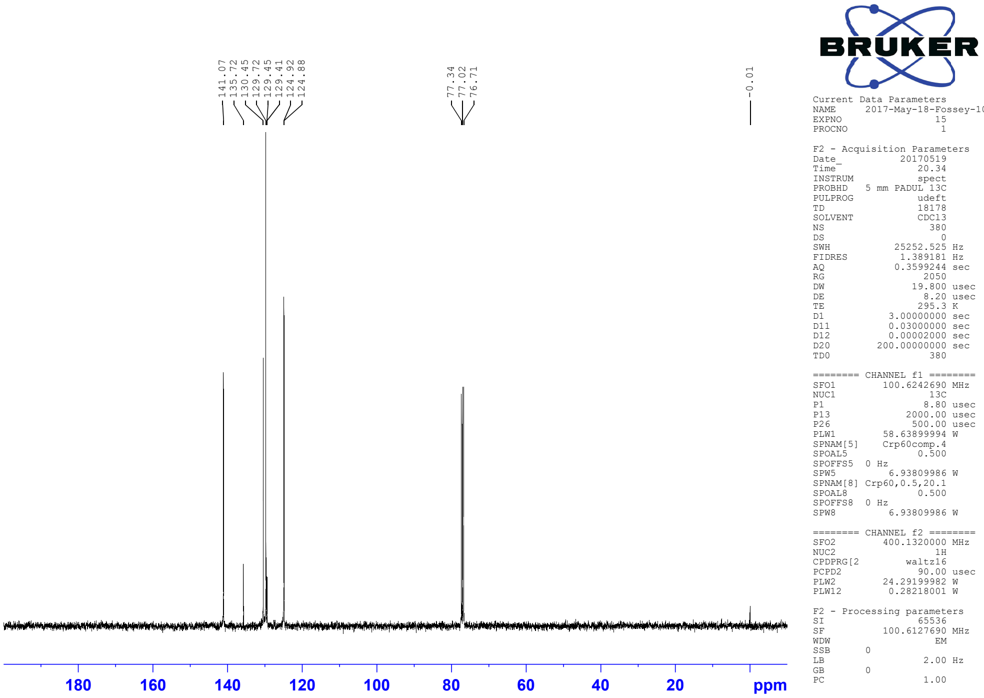

Supplementary Figure S 10. [13]*C NMR spectrum of* ***5a****.*

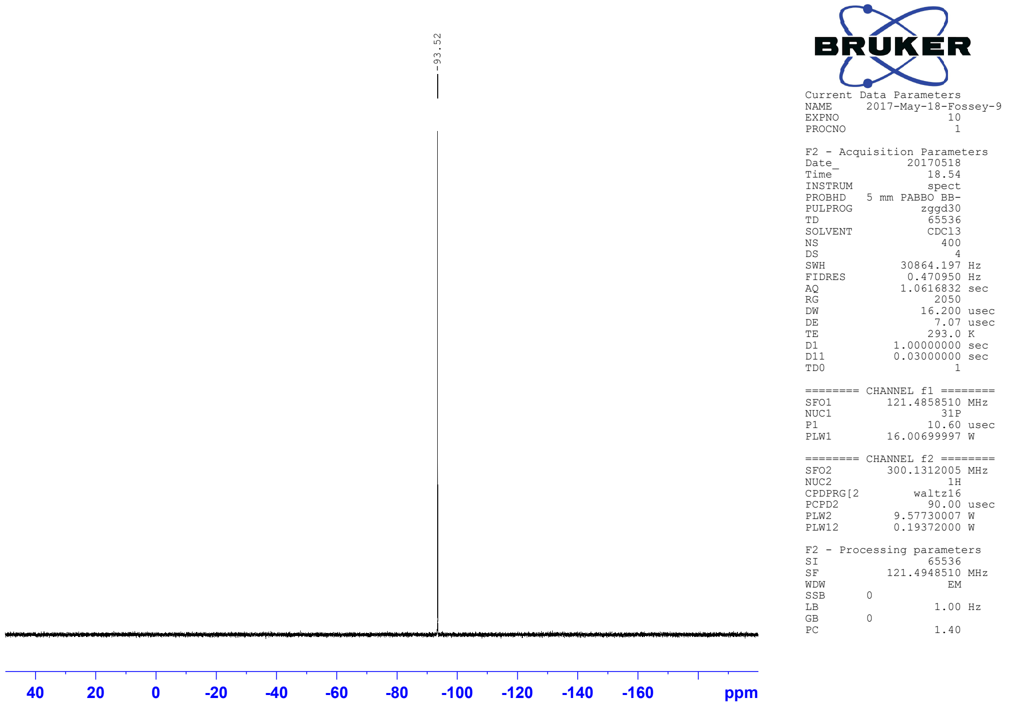

Supplementary Figure S 11. *^{31}P NMR spectrum of* ***5a***.

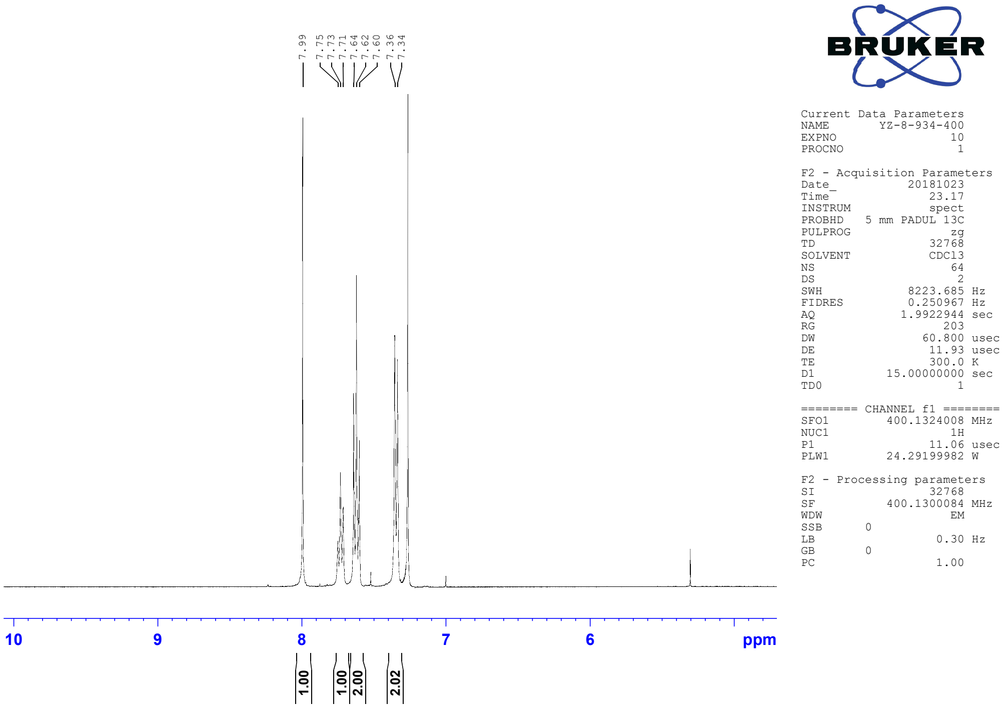

Supplementary Figure S 12. *^{1}H NMR spectrum of* ***8a***.

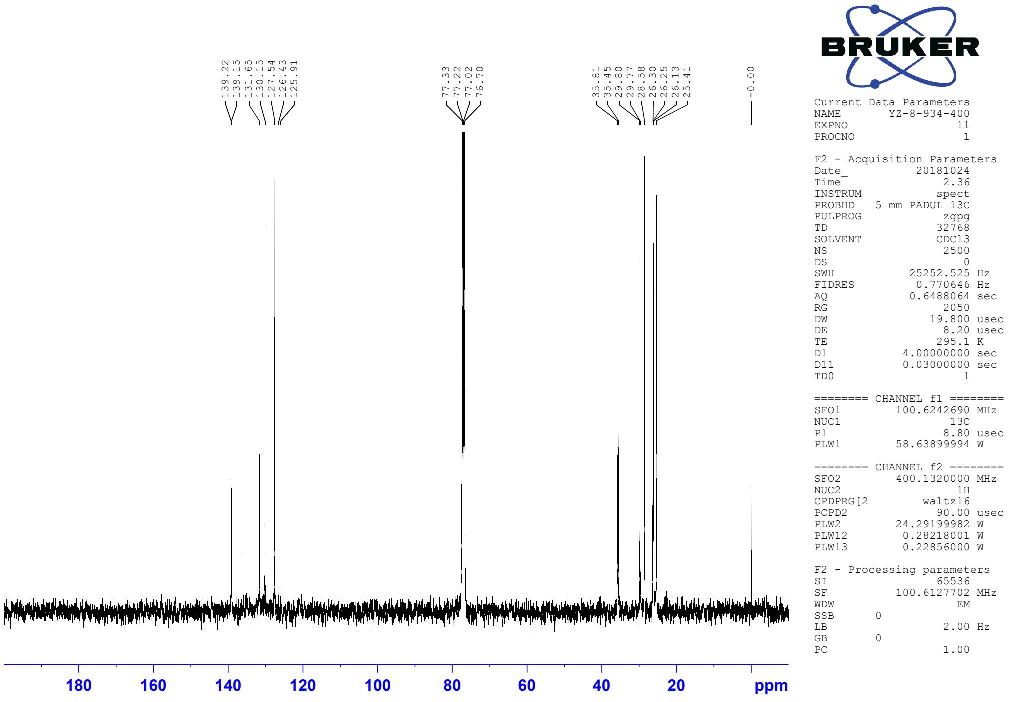

Supplementary Figure S 13. *^{13}C NMR spectrum of* ***8a***.

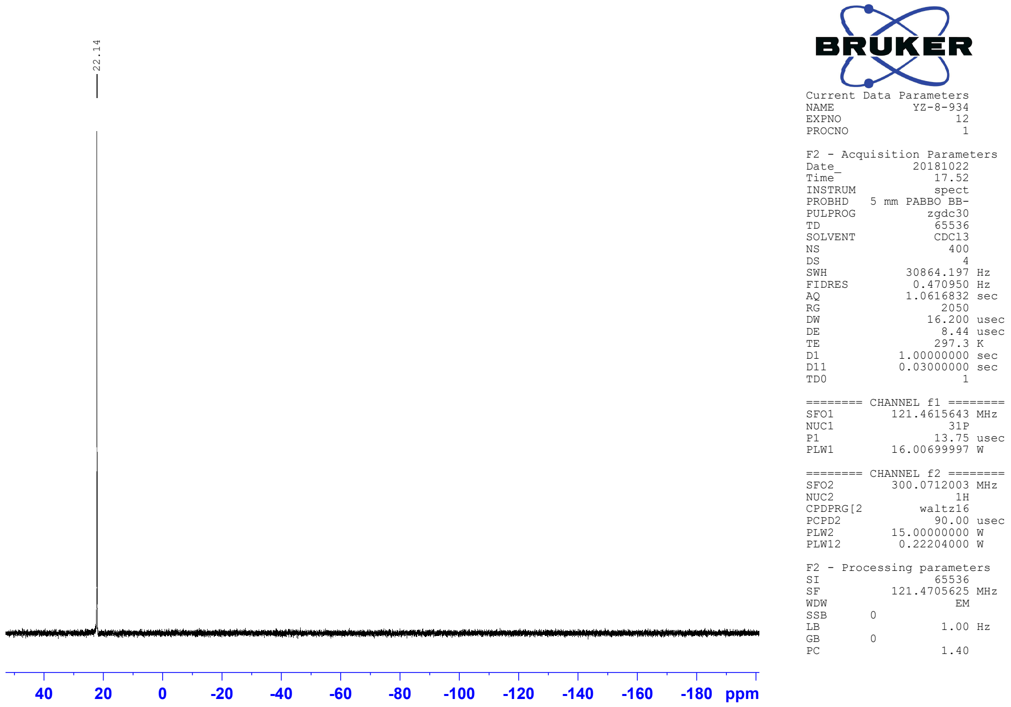

Supplementary Figure S 14. *^{31}P NMR spectrum of* ***8a***.

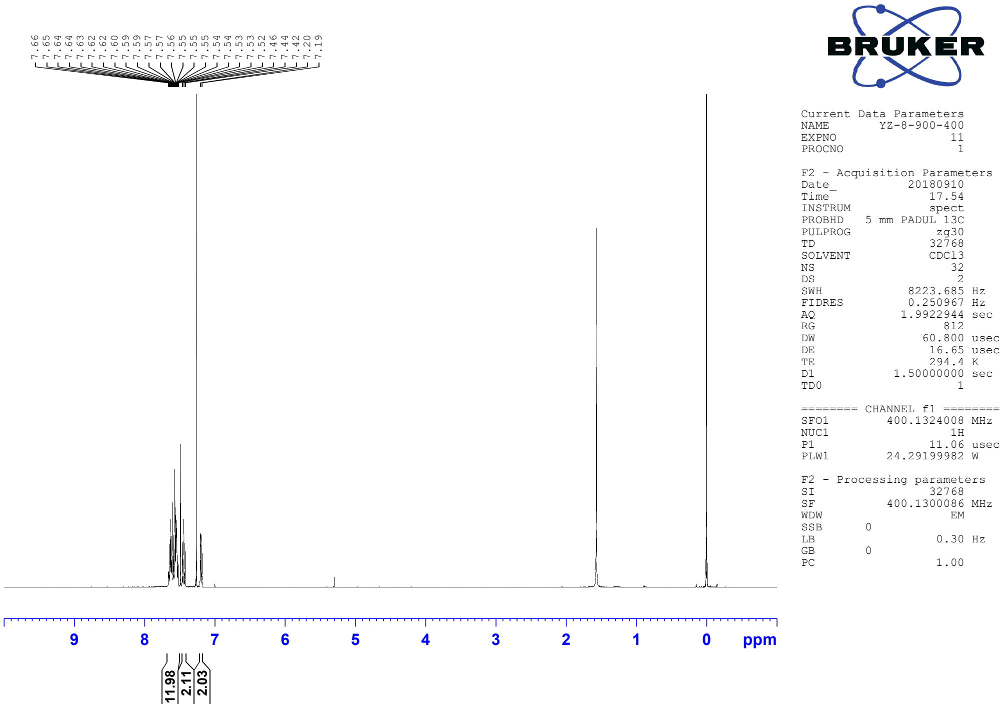

Supplementary Figure S 15. *^{1}H NMR spectrum of* ***8b****.*

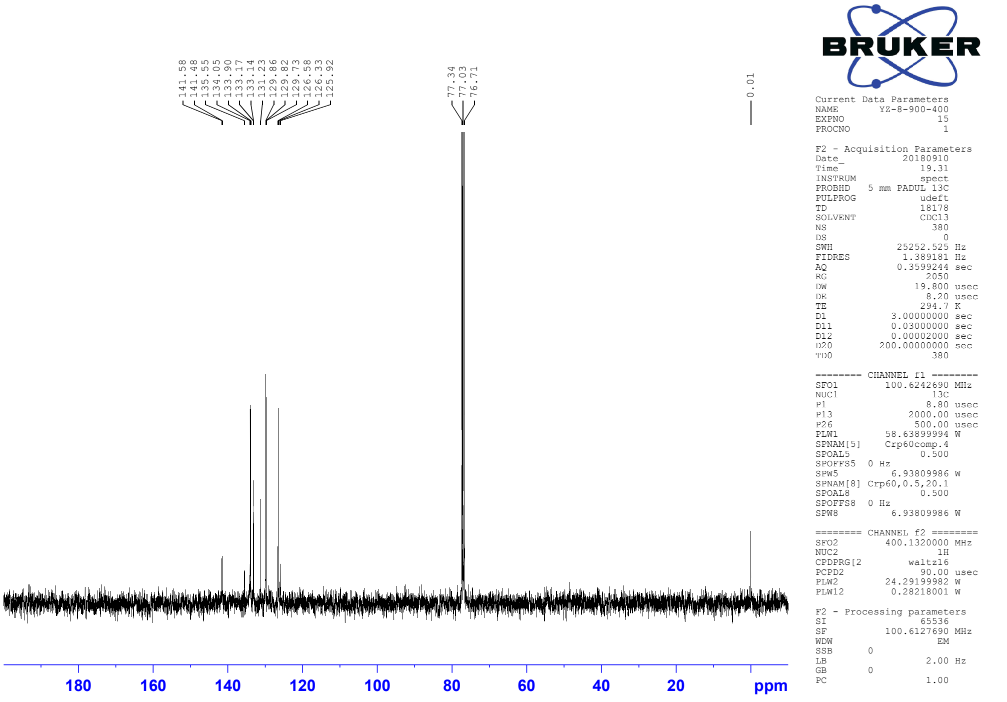

Supplementary Figure S 16. *^{13}C NMR spectrum of* ***8b****.*

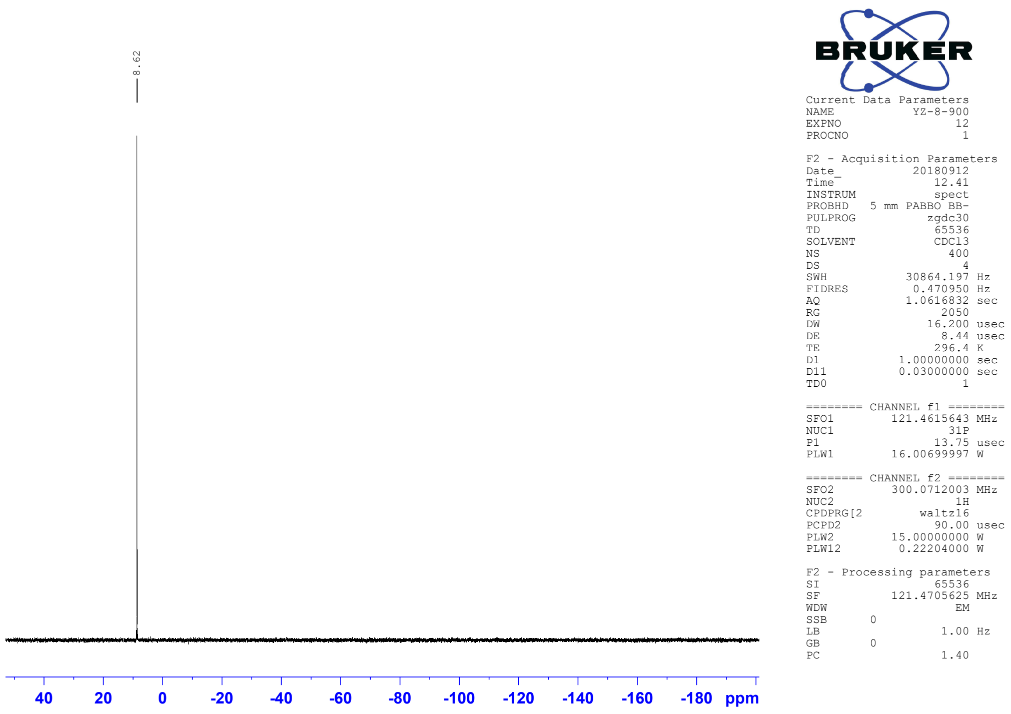

Supplementary Figure S 17. *^{31}P NMR spectrum of **8b**.*

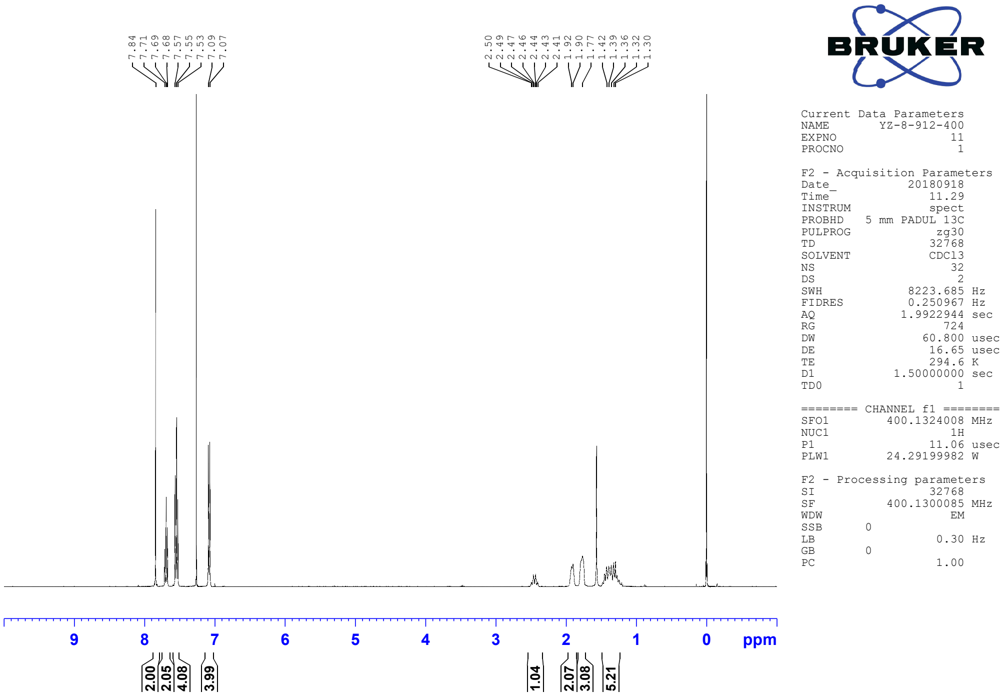

Supplementary Figure S 18. ^1H NMR spectrum of **9a**.

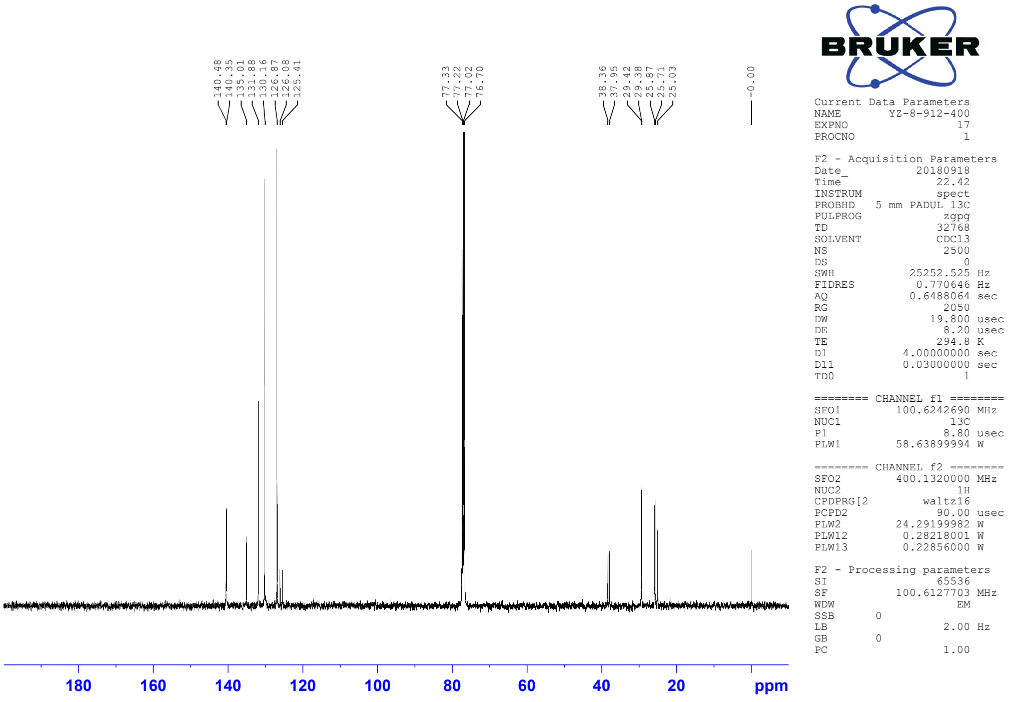

Supplementary Figure S 19. ^{13}C NMR spectrum of **9a**.

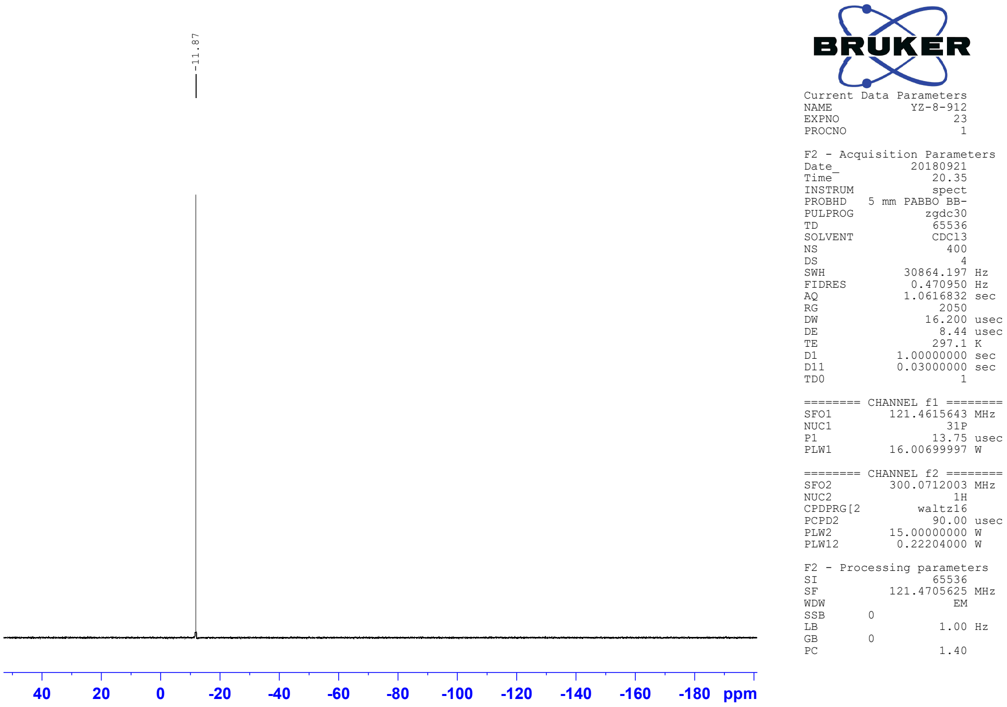

Supplementary Figure S 20. *^{31}P NMR spectrum of* ***9a***.

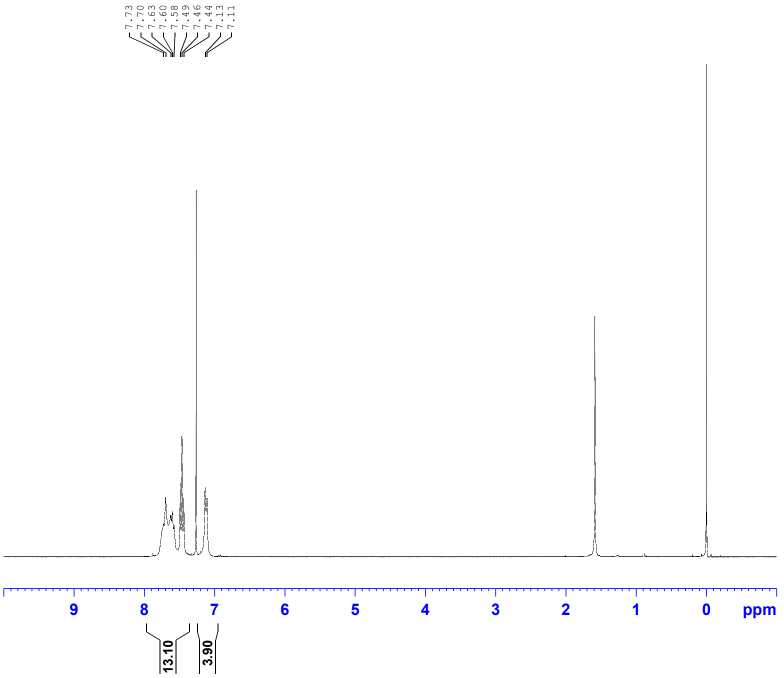

Supplementary Figure S 21. *^{1}H NMR spectrum of* ***9b***.

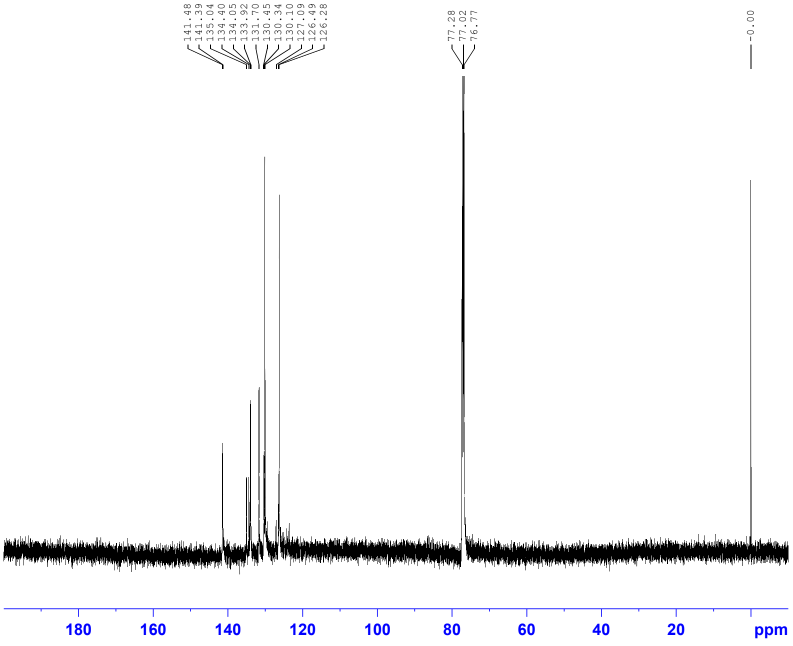

Supplementary Figure S 22. *^{13}C NMR spectrum of* ***9b***.

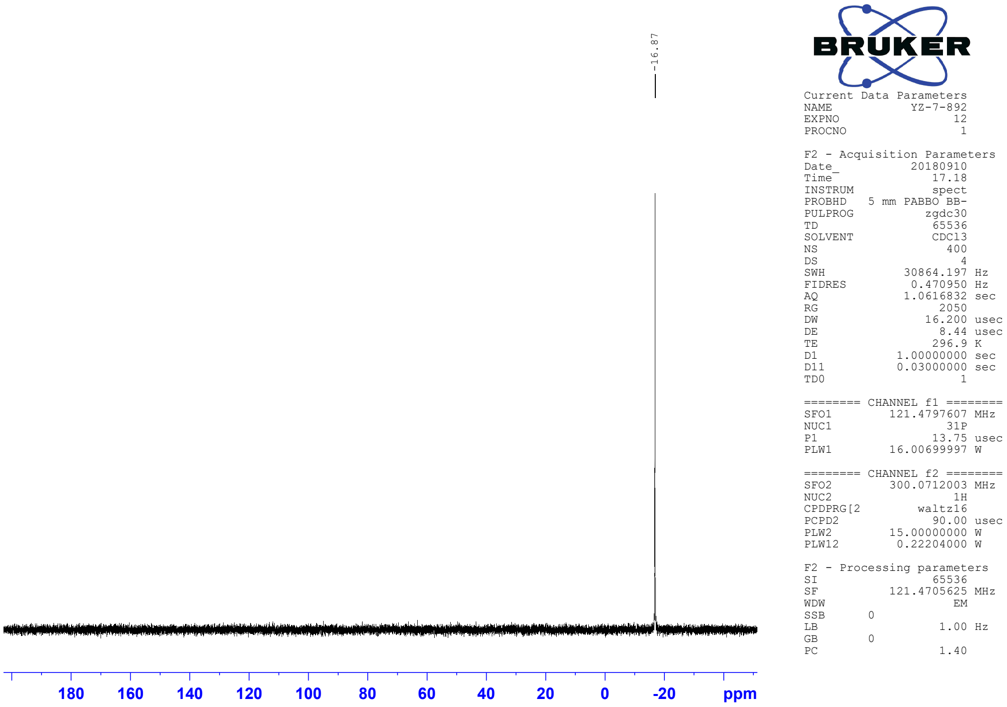

Supplementary Figure S 23. ^{31}P NMR spectrum of ***9b***.

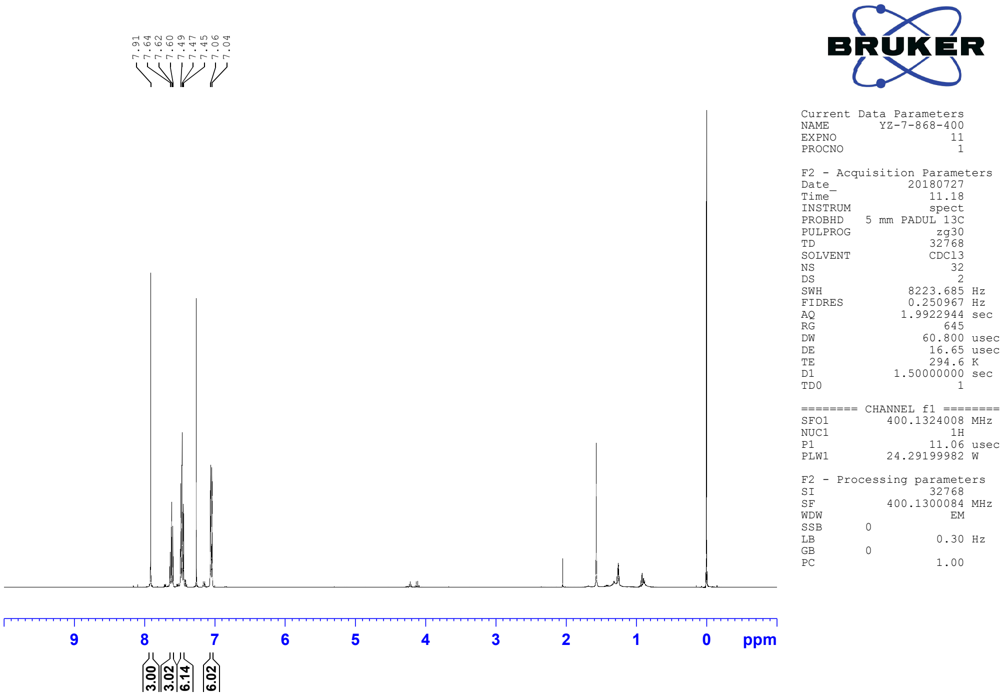

Supplementary Figure S 24. *^{1}H NMR spectrum of **10**.*

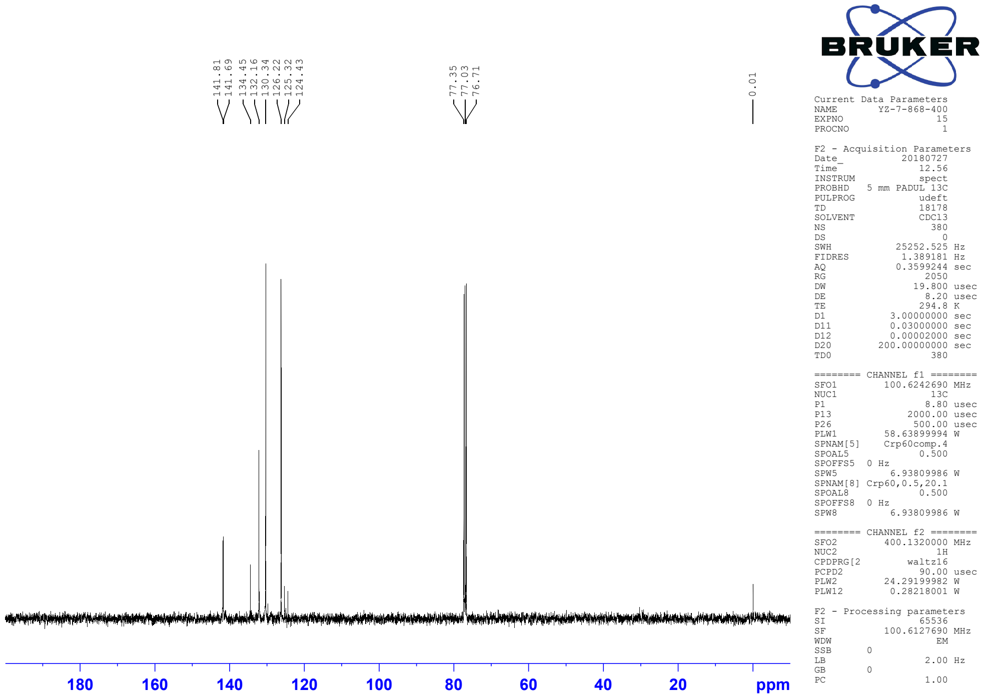

Supplementary Figure S 25. *^{13}C NMR spectrum of **10**.*

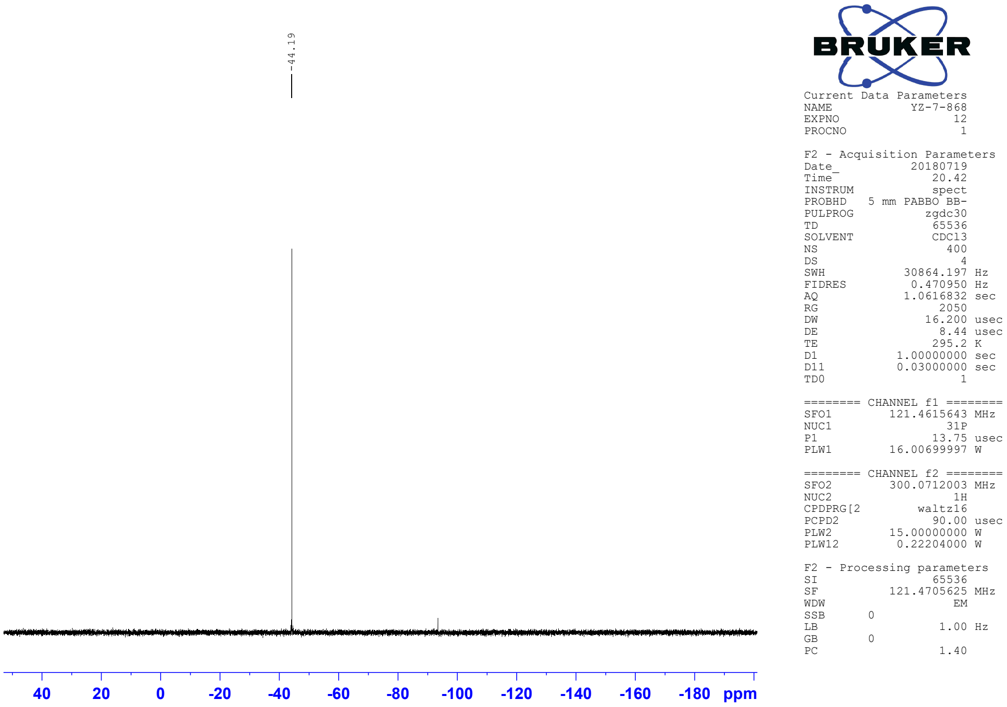

Supplementary Figure S 26. *^{31}P NMR spectrum of* ***10***.

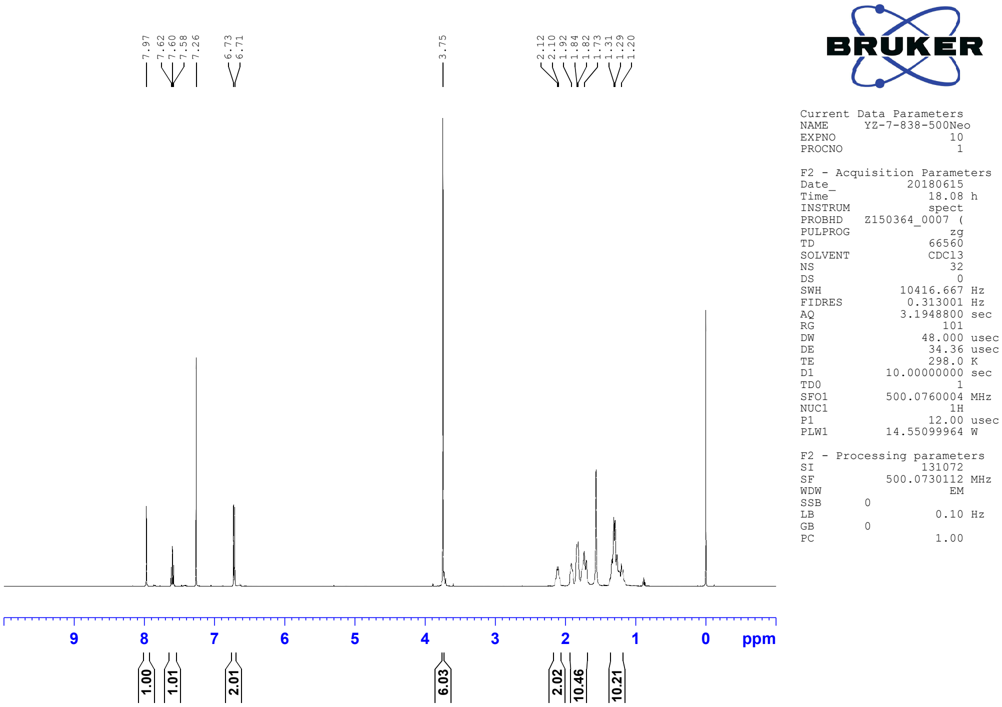

***Supplementary Figure S 27**. ^{1}H NMR spectrum of **11**.*

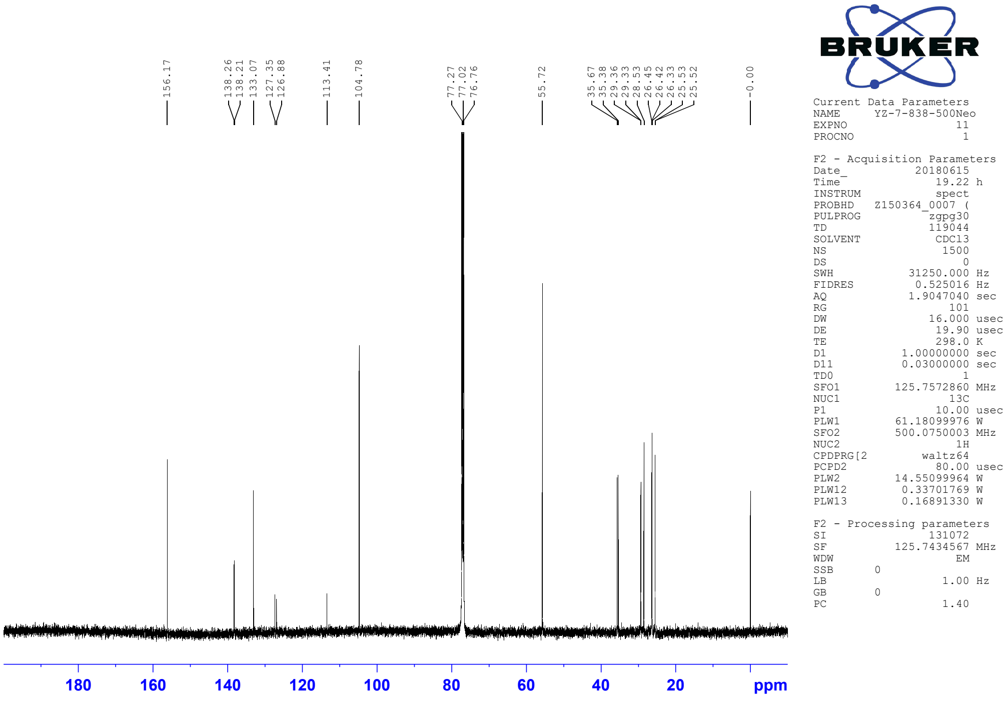

***Supplementary Figure S 28**. ^{13}C NMR spectrum of **11**.*

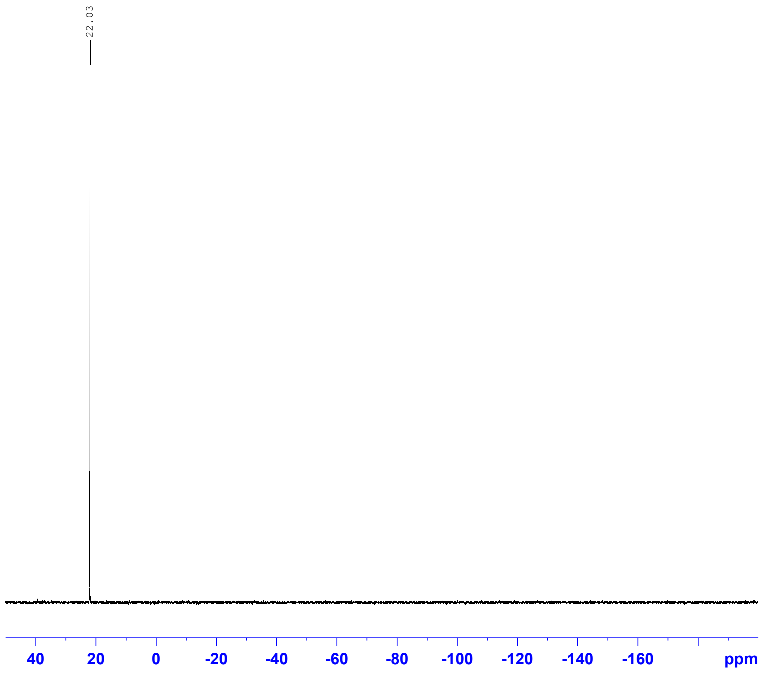

BRUKER

```
Current Data Parameters
NAME             YZ-7-838
EXPNO                  11
PROCNO                  1

F2 - Acquisition Parameters
Date_            20180614
Time                14.41
INSTRUM             spect
PROBHD   5 mm PABBO BB-
PULPROG           zgdc30
TD                  65536
SOLVENT             CDCl3
NS                    400
DS                      4
SWH             30864.197 Hz
FIDRES           0.470950 Hz
AQ              1.0616832 sec
RG                   2050
DW                 16.200 usec
DE                   8.44 usec
TE                  300.0 K
D1             1.00000000 sec
D11            0.03000000 sec
TD0                     1

======== CHANNEL f1 ========
SFO1          121.4858510 MHz
NUC1                  31P
P1                  13.75 usec
PLW1          16.00699997 W

======== CHANNEL f2 ========
SFO2          300.1312005 MHz
NUC2                   1H
CPDPRG[2          waltz16
PCPD2               90.00 usec
PLW2           9.57730007 W
PLW12          0.19372000 W

F2 - Processing parameters
SI                  65536
SF            121.4948510 MHz
WDW                    EM
SSB      0
LB                   1.00 Hz
GB       0
PC                   1.40
```

Supplementary Figure S 29. *^{31}P NMR spectrum of* ***11***.

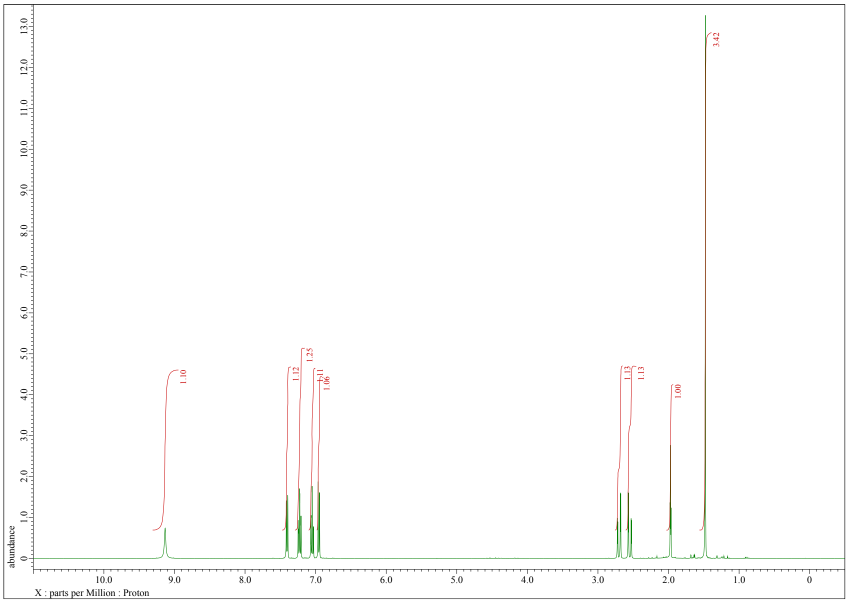

Filename = TJS-015 TT26 pure_Proton-1-2.jdf
Author = delta
Experiment = proton.jxp
Sample_Id = TJS-015 TT26 pure
Solvent = CHLOROFORM-D
Actual_Start_Time = 26-AUG-2017 03:56:46
Revision_Time = 26-AUG-2017 03:58:19

Comment = single_pulse
Data_Format = 1D COMPLEX
Dim_Size = 32768
X_Domain = Proton
Dim_Title = Proton
Dim_Units = [s]
Dimensions = X
Site = JNM-ECS400
Spectrometer = JNM-ECS400

Field_Strength = 9.38977[T] (400[MHz])
X_Acq_Duration = 2.42745[s]
X_Freq = 399.7822[MHz]
X_Offset = 9.0[ppm]
X_Points = 32768
X_Prescans = 1
X_Resolution = 0.41195[Hz]
X_Sweep = 13.49892[kHz]
X_Sweep_Clipped = 10.79914[kHz]
Irr_Domain = Proton
Irr_Freq = 399.7822[MHz]
Irr_Offset = 5[ppm]
Tri_Domain = Proton
Tri_Freq = 399.7822[MHz]
Tri_Offset = 5[ppm]
Clipped = FALSE
Scans = 16
Total_Scans = 16

Relaxation_Delay = 3[s]
Recvr_Gain = 34
Temp_Get = 19.6[dC]
X_90_Width = 10.9[us]
X_Acq_Time = 2.42745[s]
X_Angle = 45[deg]
X_Atn = 0.8[dB]
X_Pulse = 5.45[us]
Irr_Mode = Off
Tri_Mode = Off
Dante_Presat = FALSE
Initial_Wait = 1[s]
Phase = {0, 90, 270, 180, 180, 270, 90, 0}
Repetition_Time = 5.42745[s]

Supplementary Figure S 30. *^{1}H NMR spectrum of* ***13j***.

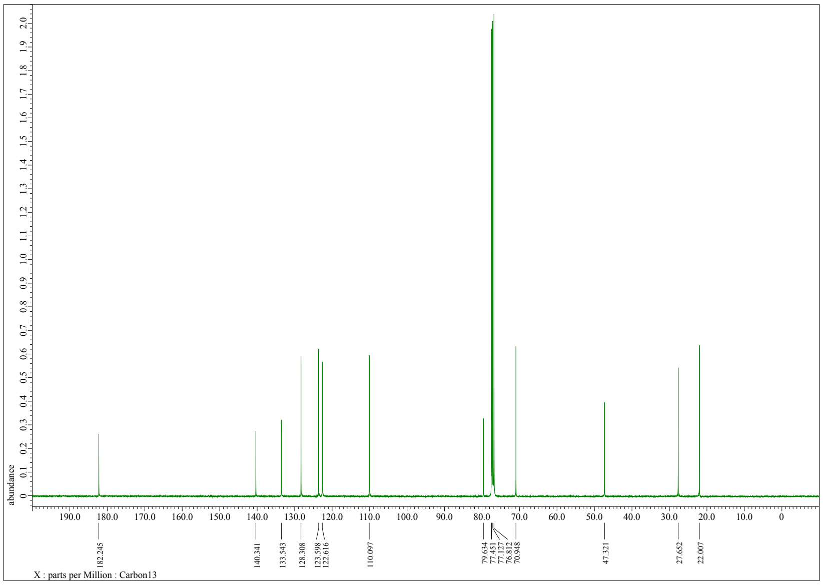

Filename = TJS-015 TT26 pure_Carbon-1-1.jdf
Author = delta
Experiment = carbon.jxp
Sample_Id = TJS-015 TT26 pure
Solvent = CHLOROFORM-D
Actual_Start_Time = 26-AUG-2017 03:59:45
Revision_Time = 26-AUG-2017 10:30:57

Comment = single pulse decoupled gated NOE
Data_Format = 1D COMPLEX
Dim_Size = 32768
X_Domain = Carbon13
Dim_Title = Carbon13
Dim_Units = [s]
Dimensions = X
Site = JNM-ECS400
Spectrometer = JNM-ECS400

Field_Strength = 9.38977[T] (400[MHz])
X_Acq_Duration = 1.04333[s]
X_Freq = 100.5253[MHz]
X_Offset = 100[ppm]
X_Points = 32768
X_Prescans = 4
X_Resolution = 0.95847[Hz]
X_Sweep = 31.40704[kHz]
X_Sweep_Clipped = 25.12563[kHz]
Irr_Domain = Proton
Irr_Freq = 399.7822[MHz]
Irr_Offset = 5[ppm]
Clipped = FALSE
Scans = 5801
Total_Scans = 5801

Relaxation_Delay = 3[s]
Recvr_Gain = 60
Temp_Get = 21.3[dC]
X_90_Width = 8[us]
X_Acq_Time = 1.04333[s]
X_Angle = 30[deg]
X_Atn = 4.5[dB]
X_Pulse = 2.66667[us]
Irr_Atn_Dec = 13.9[dB]
Irr_Atn_Noe = 13.9[dB]
Irr_Noise = WALTZ
Irr_Pwidth = 52[us]
Decoupling = TRUE
Initial_Wait = 1[s]
Noe = TRUE
Noe_Time = 3[s]
Repetition_Time = 4.04333[s]

Supplementary Figure S 31. *^{13}C NMR spectrum of* ***13j***.

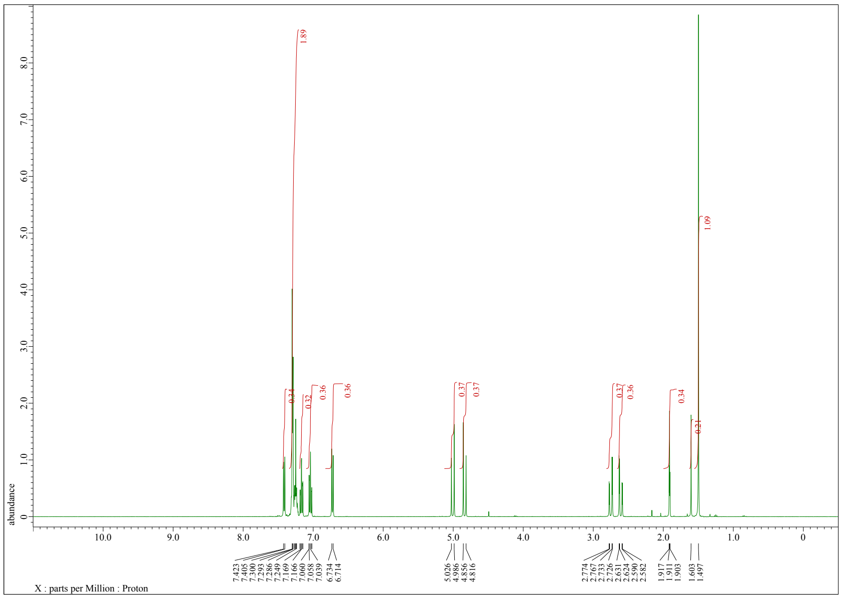

Filename = TJS-033 recyryst pure_Proton-1-1.jdf
Author = delta
Experiment = proton.jxp
Sample_Id = TJS-033 recyryst pure
Solvent = CHLOROFORM-D
Actual_Start_Time = 27-AUG-2017 00:12:27
Revision_Time = 27-AUG-2017 00:14:35

Comment = single_pulse
Data_Format = 1D COMPLEX
Dim_Size = 32768
X_Domain = Proton
Dim_Title = Proton
Dim_Units = [s]
Dimensions = X
Site = JNM-ECS400
Spectrometer = JNM-ECS400

Field_Strength = 9.38977[T] (400[MHz])
X_Acq_Duration = 2.42745[s]
X_Freq = 399.7822[MHz]
X_Offset = 9.0[ppm]
X_Points = 32768
X_Prescans = 1
X_Resolution = 0.41195[Hz]
X_Sweep = 13.49892[kHz]
X_Sweep_Clipped = 10.79914[kHz]
Irr_Domain = Proton
Irr_Freq = 399.7822[MHz]
Irr_Offset = 5[ppm]
Tri_Domain = Proton
Tri_Freq = 399.7822[MHz]
Tri_Offset = 5[ppm]
Clipped = FALSE
Scans = 16
Total_Scans = 16

Relaxation_Delay = 5[s]
Recvr_Gain = 40
Temp_Get = 19.3[dC]
X_90_Width = 10.9[us]
X_Acq_Time = 2.42745[s]
X_Angle = 45[deg]
X_Atn = 0.8[dB]
X_Pulse = 5.45[us]
Irr_Mode = Off
Tri_Mode = Off
Dante_Presat = FALSE
Initial_Wait = 1[s]
Phase = {0, 90, 270, 180, 180, 270, 90, 0}
Repetition_Time = 7.42745[s]

Supplementary Figure S 32. *^{1}H NMR spectrum of* ***13k***.

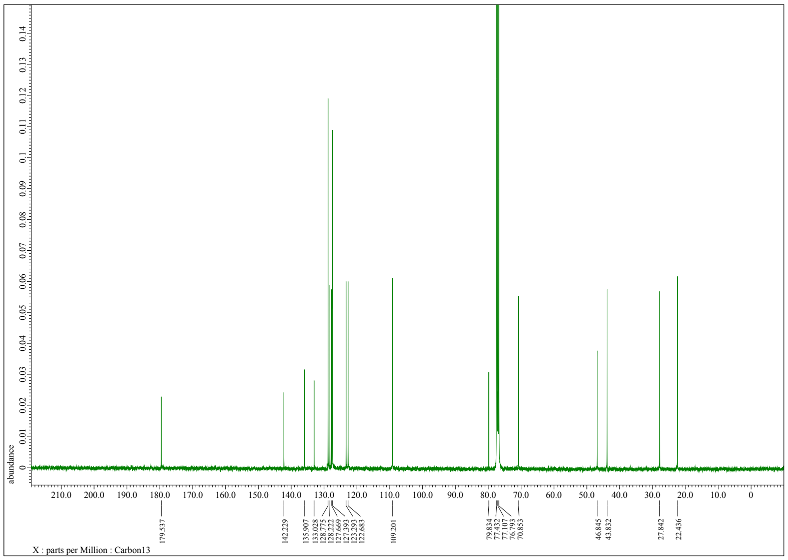

Filename = TJS-033 recyryst pure_Carbon-1-1.jdf
Author = delta
Experiment = carbon.jxp
Sample_Id = TJS-033 recyryst pure
Solvent = CHLOROFORM-D
Actual_Start_Time = 27-AUG-2017 00:14:42
Revision_Time = 27-AUG-2017 07:10:27

Comment = single pulse decoupled gated NOE
Data_Format = 1D COMPLEX
Dim_Size = 32768
X_Domain = Carbon13
Dim_Title = Carbon13
Dim_Units = [s]
Dimensions = X
Site = JNM-ECS400
Spectrometer = JNM-ECS400

Field_Strength = 9.38977[T] (400[MHz])
X_Acq_Duration = 1.04333[s]
X_Freq = 100.5253[MHz]
X_Offset = 100[ppm]
X_Points = 32768
X_Prescans = 4
X_Resolution = 0.95847[Hz]
X_Sweep = 31.40704[kHz]
X_Sweep_Clipped = 25.12563[kHz]
Irr_Domain = Proton
Irr_Freq = 399.7822[MHz]
Irr_Offset = 5[ppm]
Clipped = TRUE
Scans = 8192
Total_Scans = 8192

Relaxation_Delay = 2[s]
Recvr_Gain = 50
Temp_Get = 21.1[dC]
X_90_Width = 8[us]
X_Acq_Time = 1.04333[s]
X_Angle = 30[deg]
X_Atn = 4.5[dB]
X_Pulse = 2.66667[us]
Irr_Atn_Dec = 13.9[dB]
Irr_Atn_Noe = 13.9[dB]
Irr_Noise = WALTZ
Irr_Pwidth = 52[us]
Decoupling = TRUE
Initial_Wait = 1[s]
Noe = TRUE
Noe_Time = 2[s]
Repetition_Time = 3.04333[s]

Supplementary Figure S 33. *^{13}C NMR spectrum of* ***13k***.

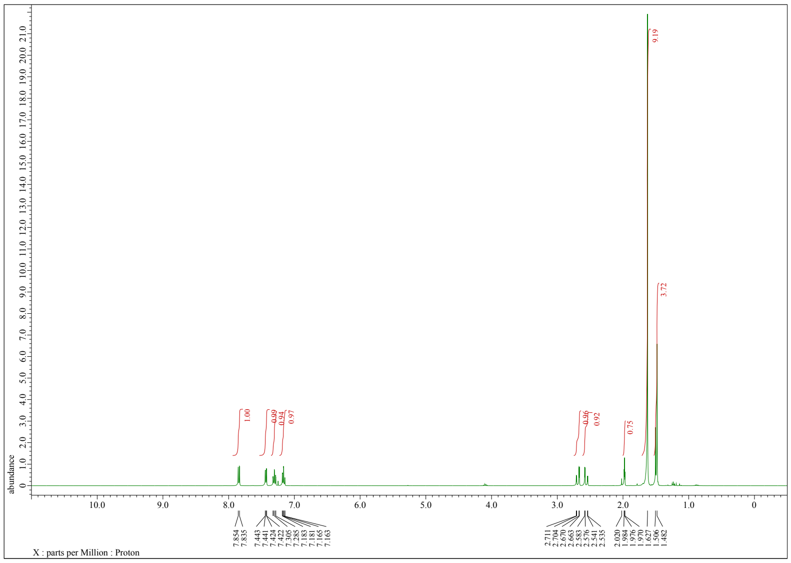

Filename	= TJS-043 TT9-10 pure_proton-1-1.jdf
Author	= delta
Experiment	= proton.jxp
Sample_Id	= TJS-043 TT9-10 pure
Solvent	= CHLOROFORM-D
Actual_Start_Time	= 31-AUG-2017 13:04:54
Revision_Time	= 31-AUG-2017 13:06:22
Comment	= single_pulse
Data_Format	= 1D COMPLEX
Dim_Size	= 32768
X_Domain	= Proton
Dim_Title	= Proton
Dim_Units	= [s]
Dimensions	= X
Site	= JNM-ECS400
Spectrometer	= JNM-ECS400
Field_Strength	= 9.38977[T] (400[MHz])
X_Acq_Duration	= 2.42745[s]
X_Freq	= 399.7822[MHz]
X_Offset	= 9.0[ppm]
X_Points	= 32768
X_Prescans	= 0
X_Resolution	= 0.41195[Hz]
X_Sweep	= 13.49892[kHz]
X_Sweep_Clipped	= 10.79914[kHz]
Irr_Domain	= Proton
Irr_Freq	= 399.7822[MHz]
Irr_Offset	= 5[ppm]
Tri_Domain	= Proton
Tri_Freq	= 399.7822[MHz]
Tri_Offset	= 5[ppm]
Clipped	= FALSE
Scans	= 16
Total_Scans	= 16
Relaxation_Delay	= 3[s]
Recvr_Gain	= 28
Temp_Get	= 18.8[dC]
X_90_Width	= 10.9[us]
X_Acq_Time	= 2.42745[s]
X_Angle	= 45[deg]
X_Atn	= 0.8[dB]
X_Pulse	= 5.45[us]
Irr_Mode	= Off
Tri_Mode	= Off
Dante_Presat	= FALSE
Initial_Wait	= 1[s]
Phase	= {0, 90, 270, 180, 180, 270, 90, 0}
Repetition_Time	= 5.42745[s]

Supplementary Figure S 34. *^{1}H NMR spectrum of* ***13l***.

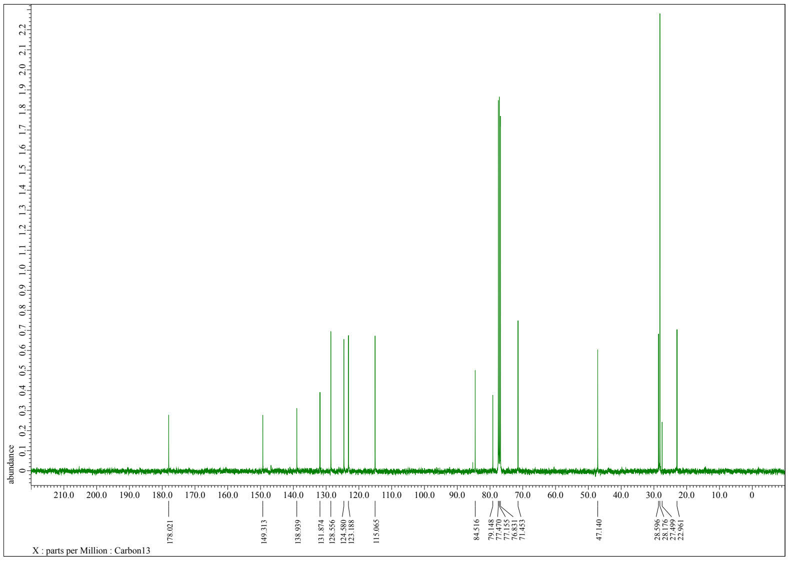

Filename	= TJS-043 TT9-10 pure_carbon-1-1.jdf
Author	= delta
Experiment	= carbon.jxp
Sample_Id	= TJS-043 TT9-10 pure
Solvent	= CHLOROFORM-D
Actual_Start_Time	= 1-SEP-2017 11:25:12
Revision_Time	= 1-SEP-2017 11:35:44
Comment	= single pulse decoupled gated NOE
Data_Format	= 1D COMPLEX
Dim_Size	= 32768
X_Domain	= Carbon13
Dim_Title	= Carbon13
Dim_Units	= [s]
Dimensions	= X
Site	= JNM-ECS400
Spectrometer	= JNM-ECS400
Field_Strength	= 9.38977[T] (400[MHz])
X_Acq_Duration	= 1.04333[s]
X_Freq	= 100.5253[MHz]
X_Offset	= 100[ppm]
X_Points	= 32768
X_Prescans	= 4
X_Resolution	= 0.95847[Hz]
X_Sweep	= 31.40704[kHz]
X_Sweep_Clipped	= 25.12563[kHz]
Irr_Domain	= Proton
Irr_Freq	= 399.7822[MHz]
Irr_Offset	= 5[ppm]
Clipped	= FALSE
Scans	= 152
Total_Scans	= 152
Relaxation_Delay	= 3[s]
Recvr_Gain	= 60
Temp_Get	= 20.3[dC]
X_90_Width	= 8[us]
X_Acq_Time	= 1.04333[s]
X_Angle	= 30[deg]
X_Atn	= 4.5[dB]
X_Pulse	= 2.66667[us]
Irr_Atn_Dec	= 13.9[dB]
Irr_Atn_Noe	= 13.9[dB]
Irr_Noise	= WALTZ
Irr_Pwidth	= 52[us]
Decoupling	= TRUE
Initial_Wait	= 1[s]
Noe	= TRUE
Noe_Time	= 3[s]
Repetition_Time	= 4.04333[s]

Supplementary Figure S 35. *^{13}C NMR spectrum of* ***13l***.

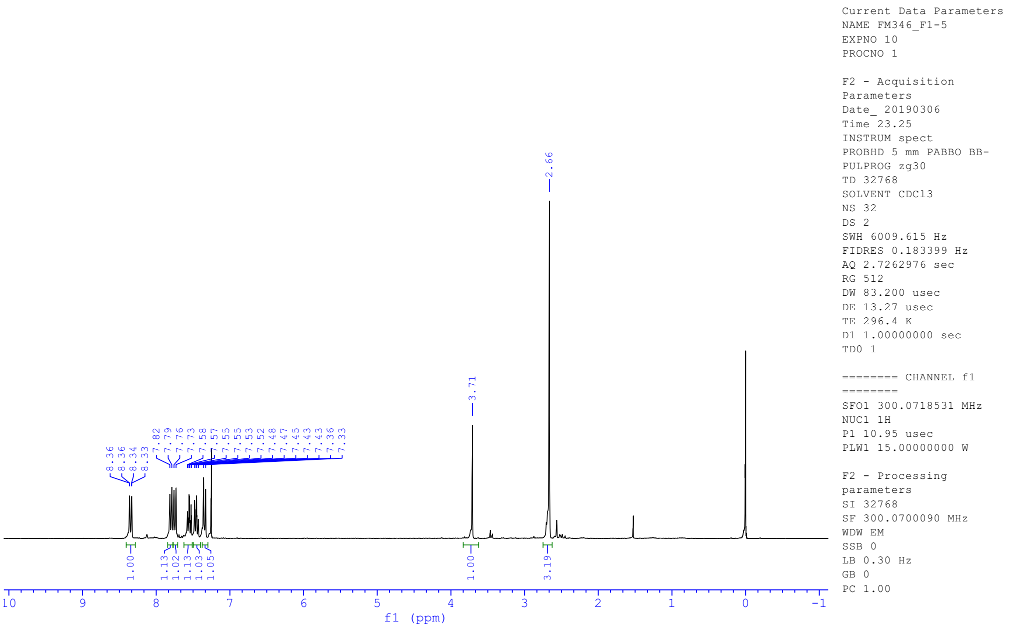

Supplementary Figure S 36. *¹H NMR spectrum of **13e**.*

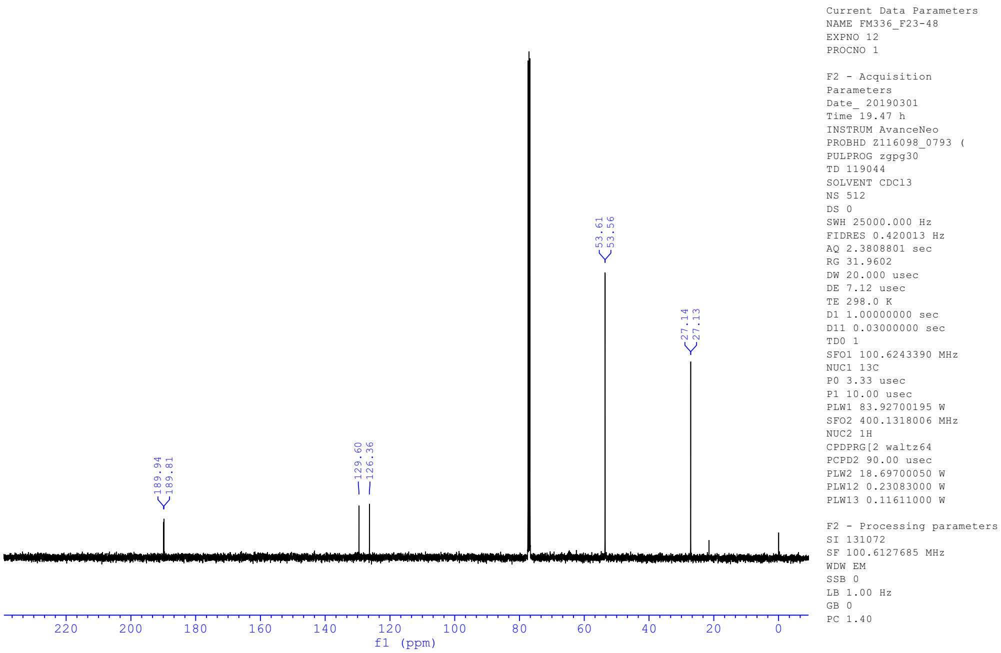

Supplementary Figure S 37. *¹³C NMR spectrum of **13e**.*

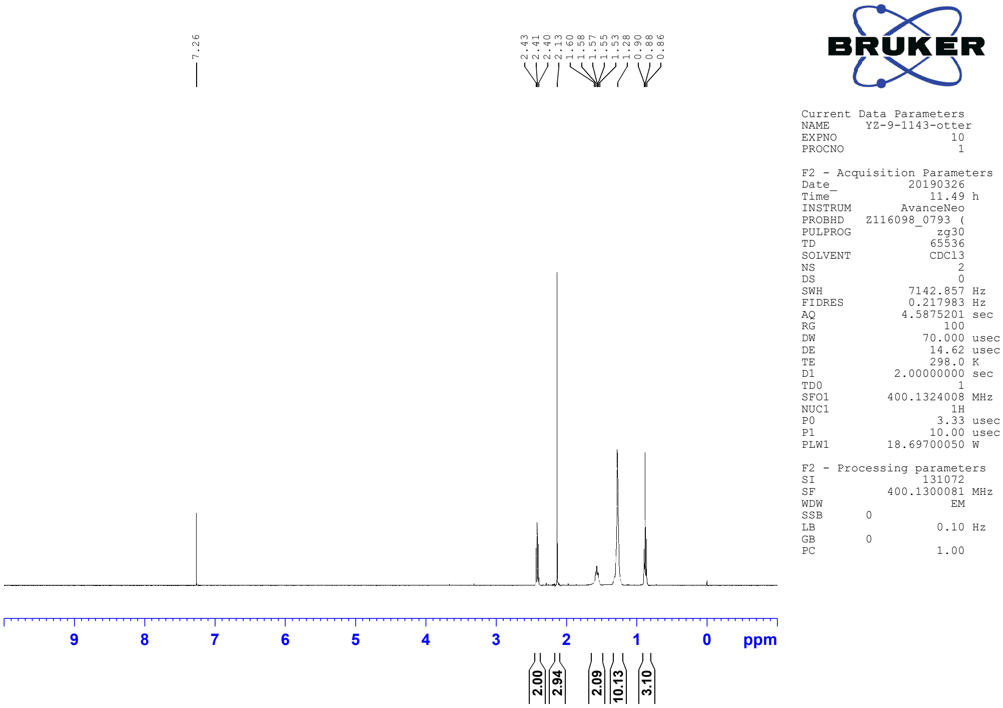

Supplementary Figure S 38. *^{1}H NMR spectrum of* ***14a****.*

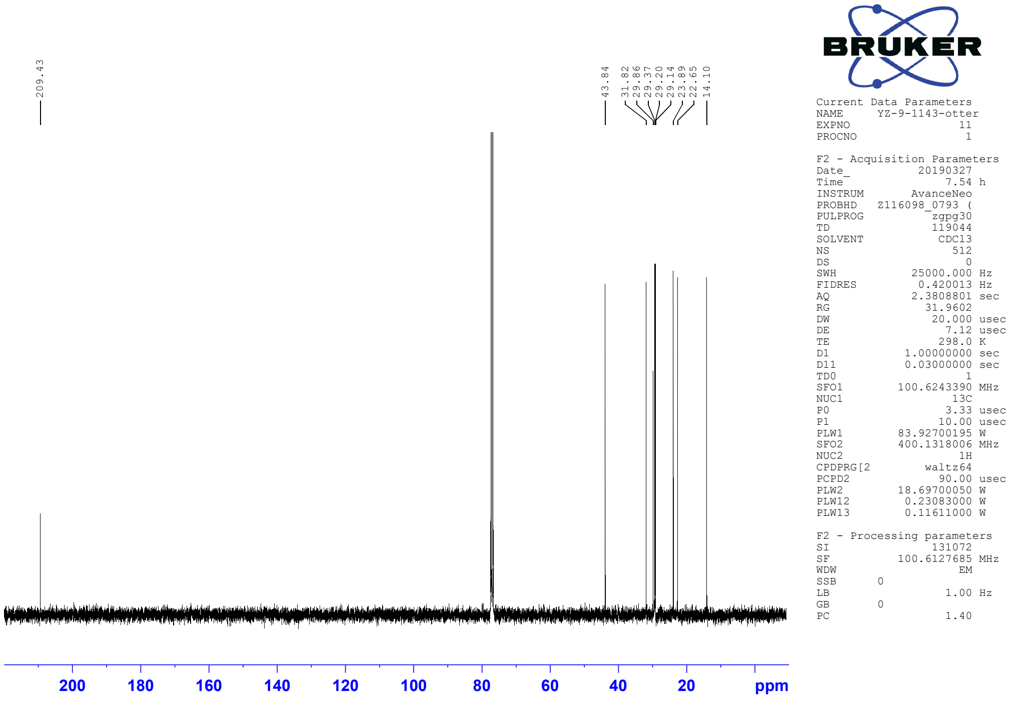

Supplementary Figure S 39. *^{13}C NMR spectrum of* ***14a****.*

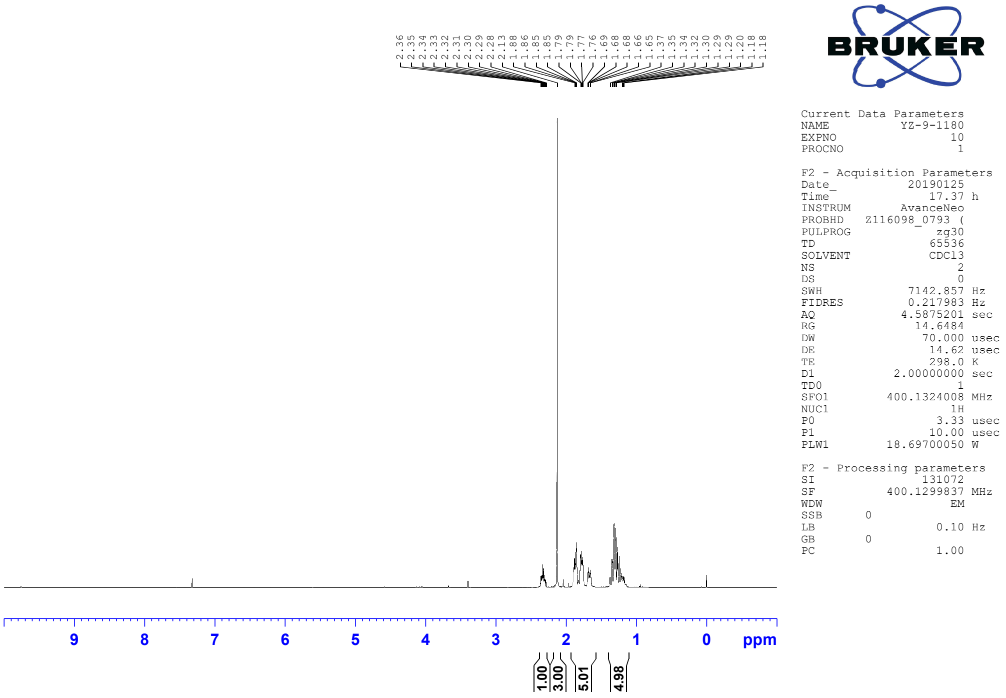

Supplementary Figure S 40. *^{1}H NMR spectrum of* ***14b***.

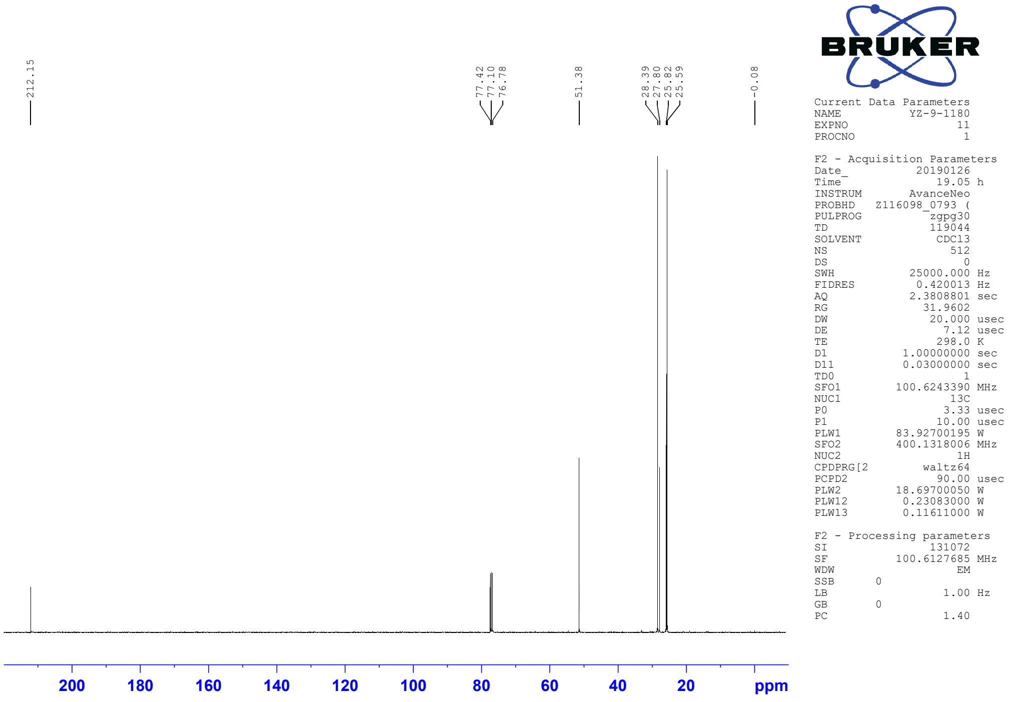

Supplementary Figure S 41. *^{13}C NMR spectrum of* ***14b***.

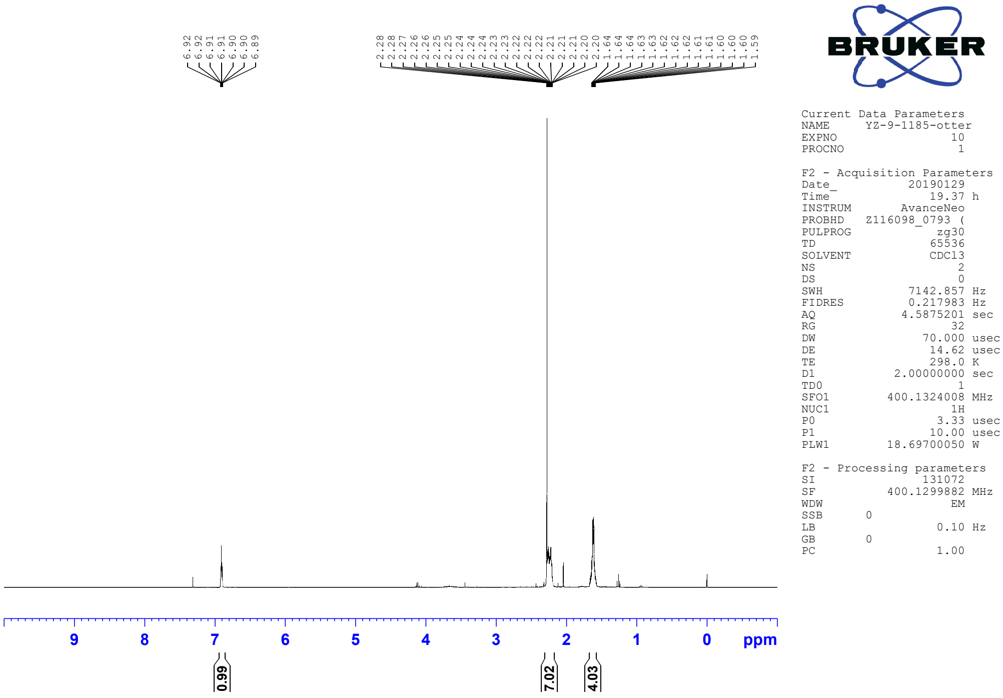

Supplementary Figure S 42. ^{1}H *NMR spectrum of* ***14c***.

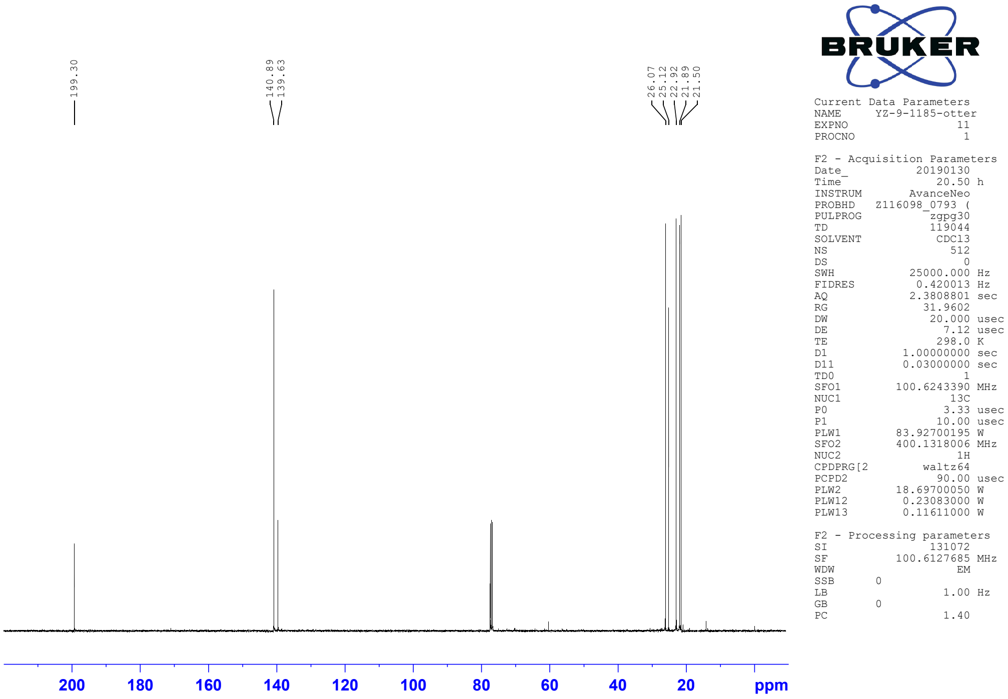

Supplementary Figure S 43. ^{13}C *NMR spectrum of* ***14c***.

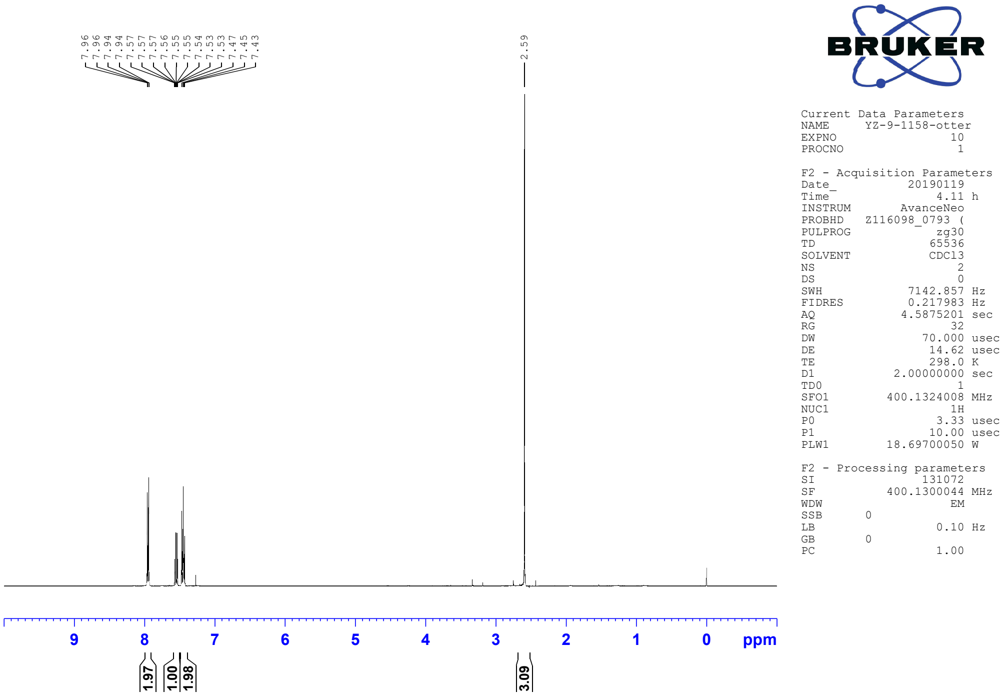

Supplementary Figure S 44. *^{1}H NMR spectrum of* ***14d****.*

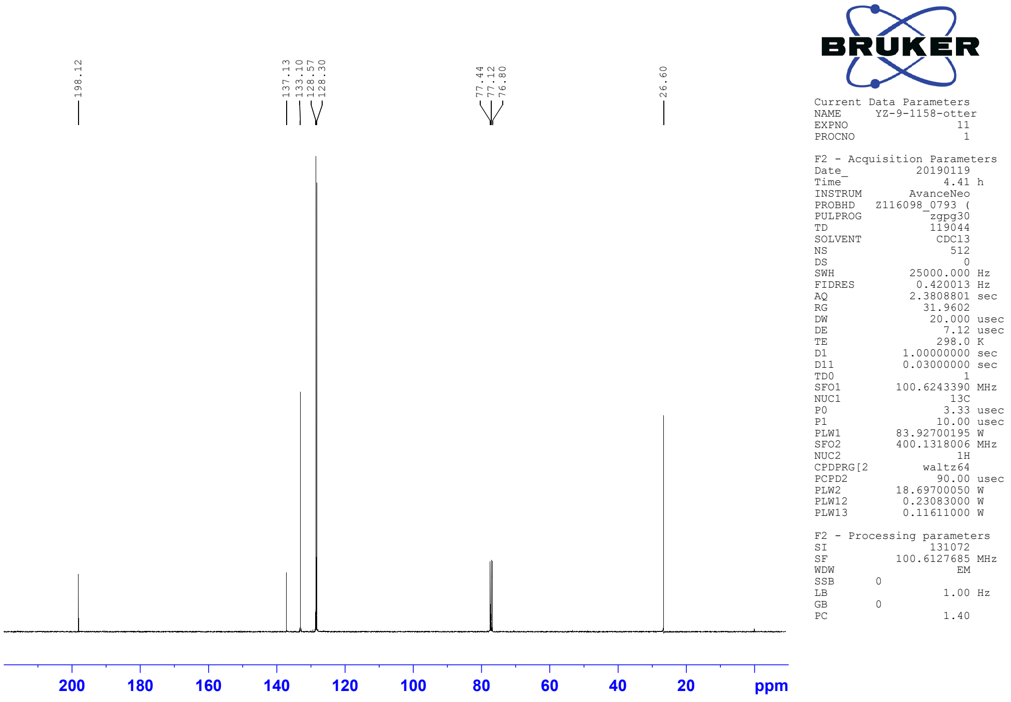

Supplementary Figure S 45. *^{13}C NMR spectrum of* ***14d****.*

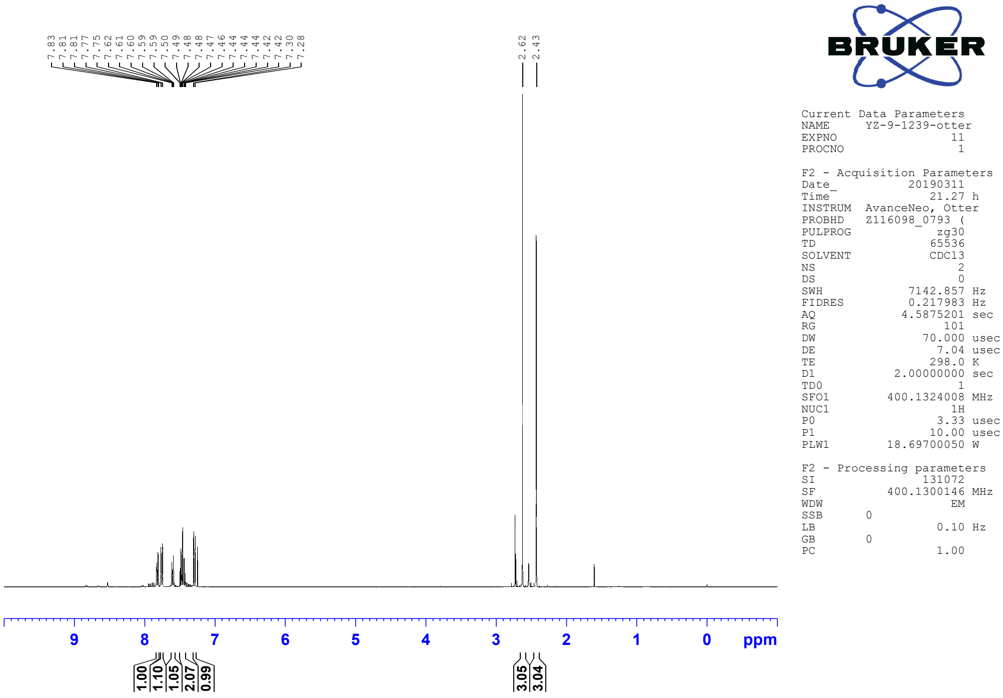

Supplementary Figure S 46. *^{1}H NMR spectrum of* ***14e****.*

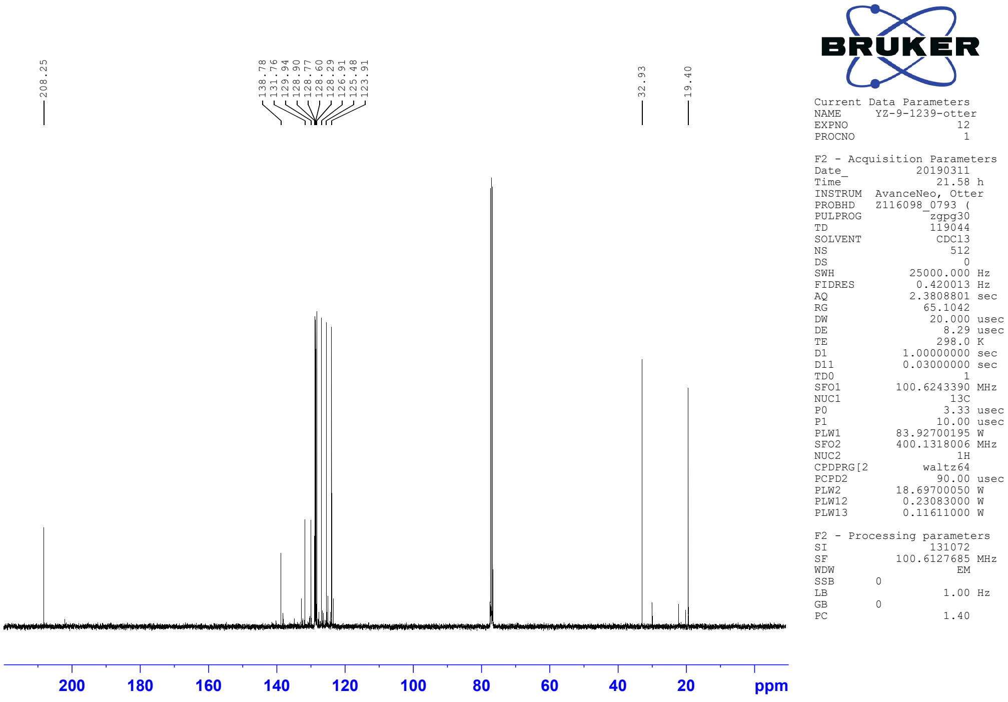

Supplementary Figure S 47. *^{13}C NMR spectrum of* ***14e****.*

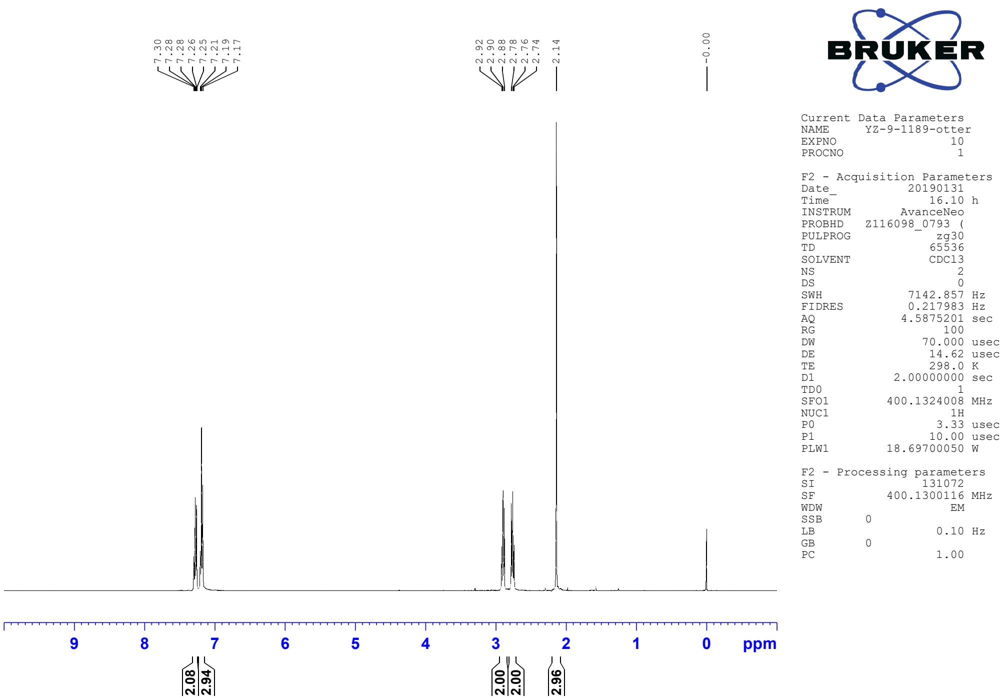

Supplementary Figure S 48. *^{1}H NMR spectrum of* ***14f***.

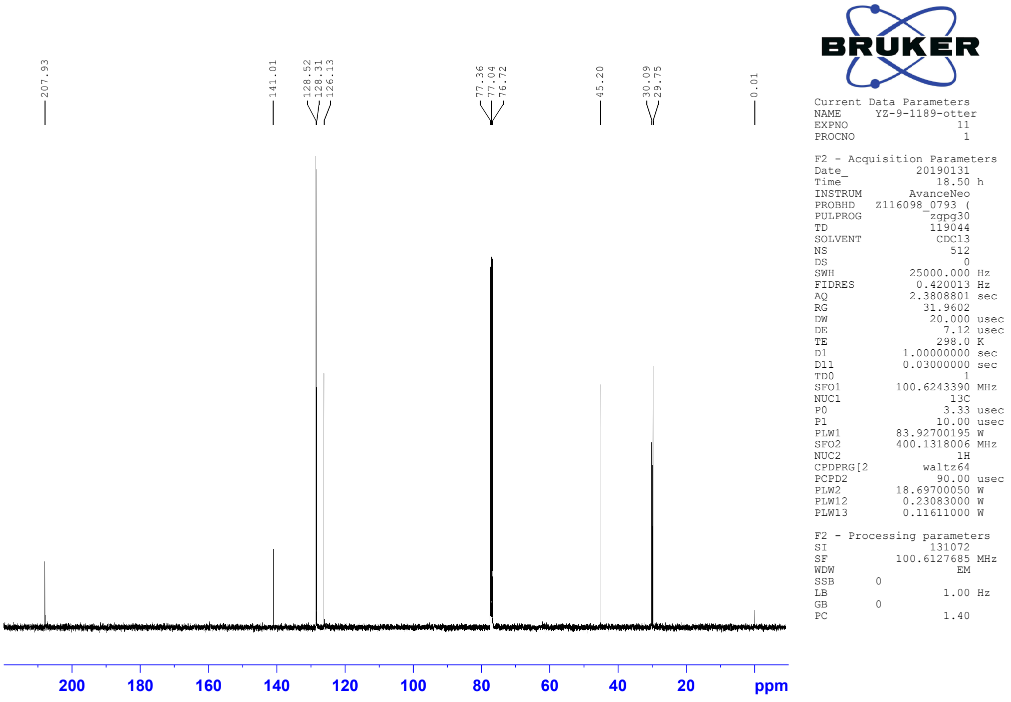

Supplementary Figure S 49. *^{13}C NMR spectrum of* ***14f***.

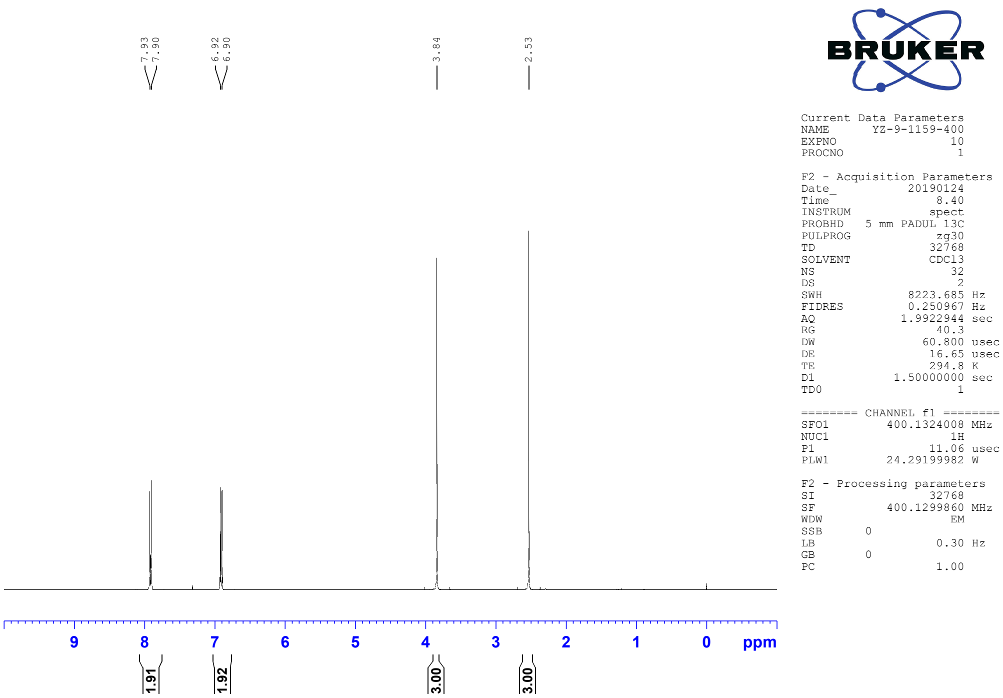

Supplementary Figure S 50. ^{1}H *NMR spectrum of* ***14g***.

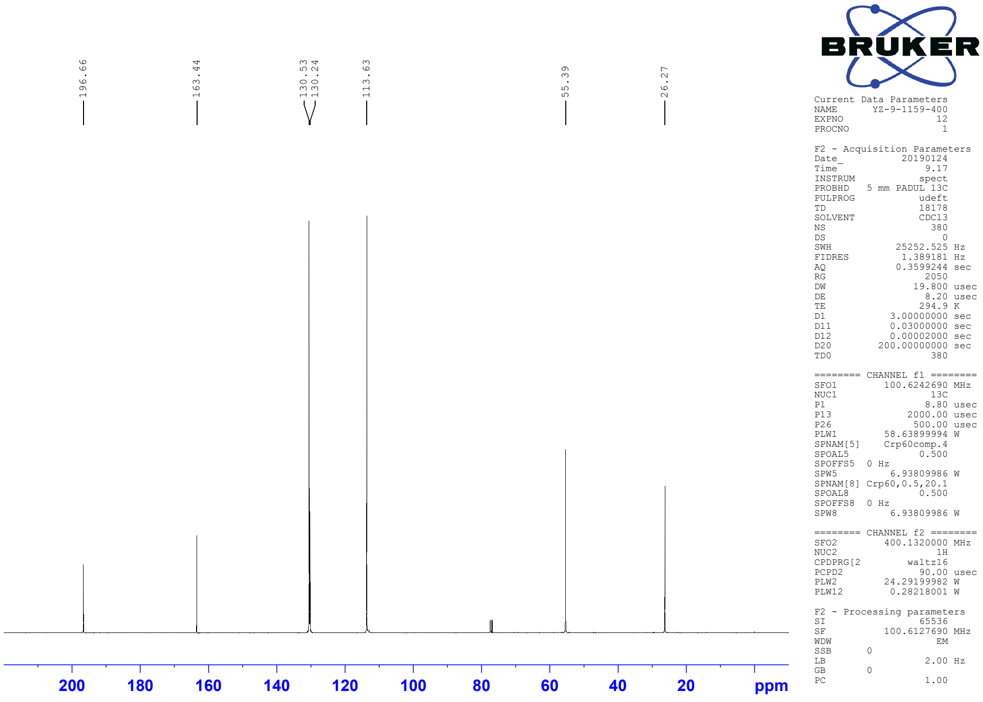

Supplementary Figure S 51. ^{13}C *NMR spectrum of* ***14g***.

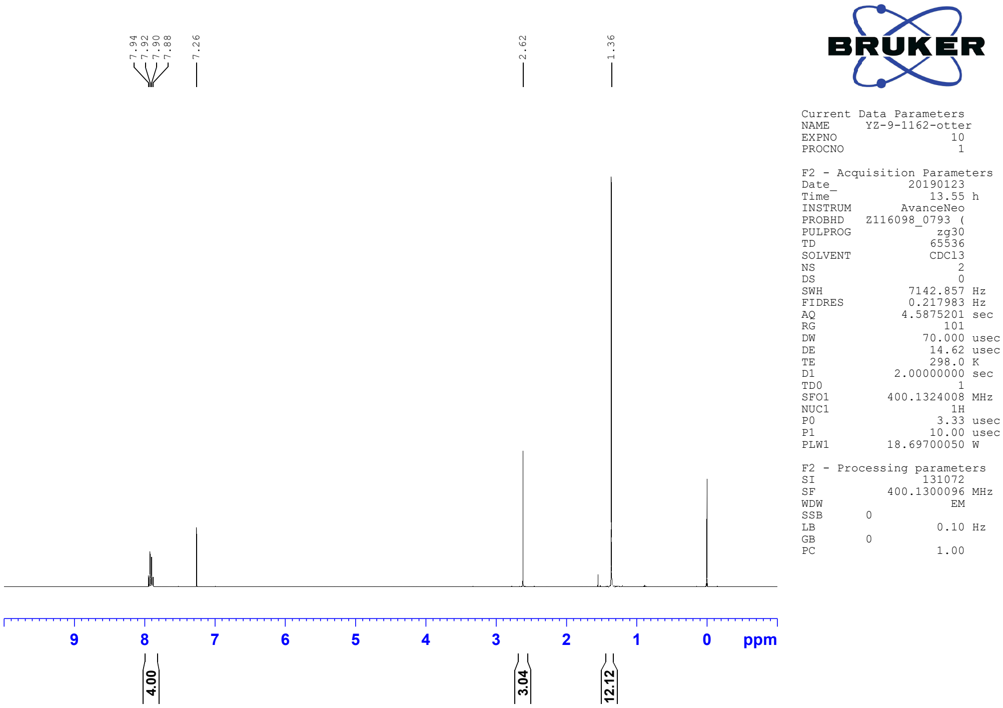

Supplementary Figure S 52. [1]*H NMR spectrum of* ***14h****.*

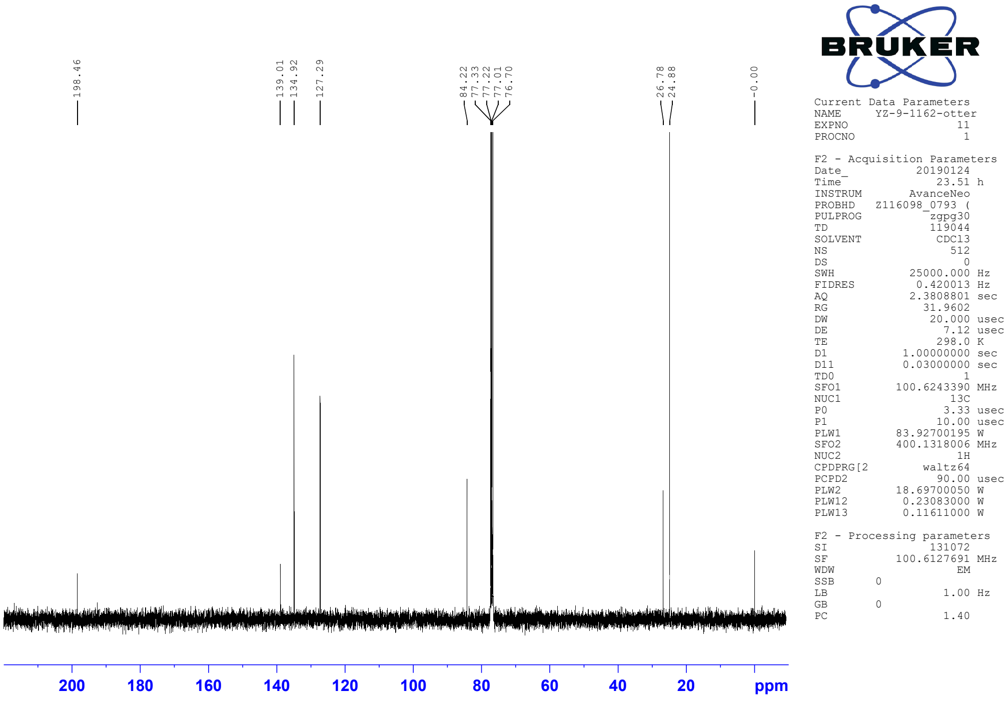

Supplementary Figure S 53. [13]*C NMR spectrum of* ***14h****.*

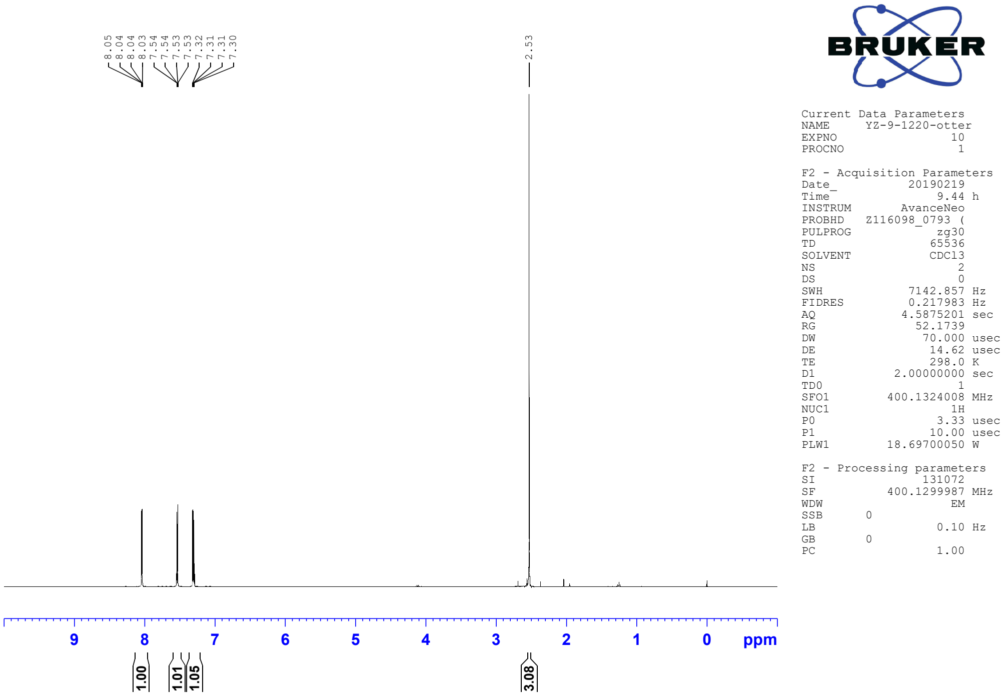

Supplementary Figure S 54. *^{1}H NMR spectrum of* ***14i****.*

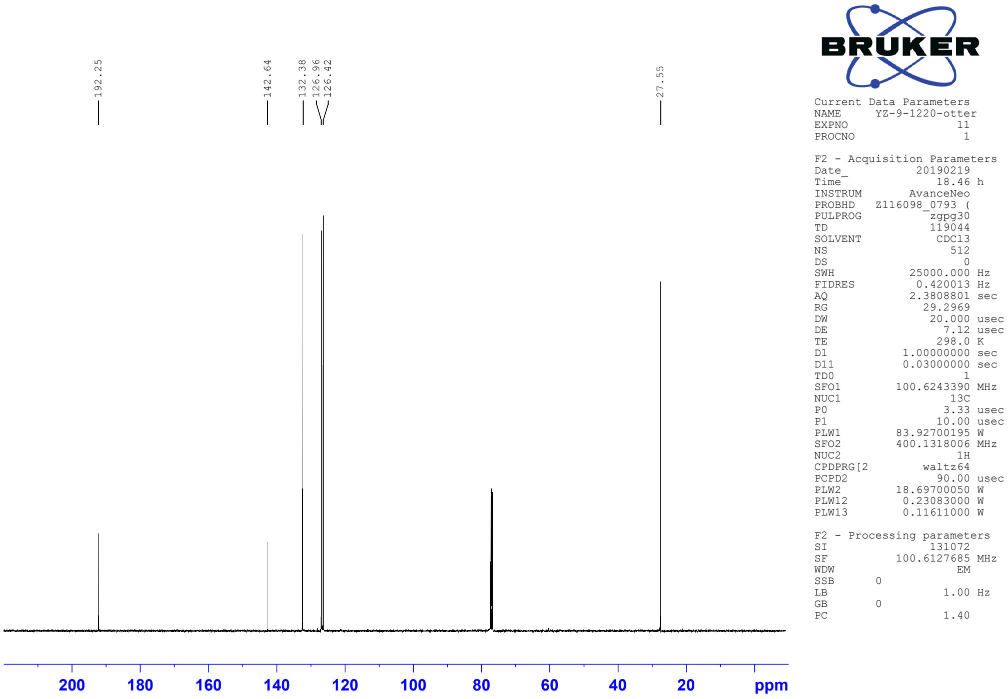

Supplementary Figure S 55. *^{13}C NMR spectrum of* ***14i****.*

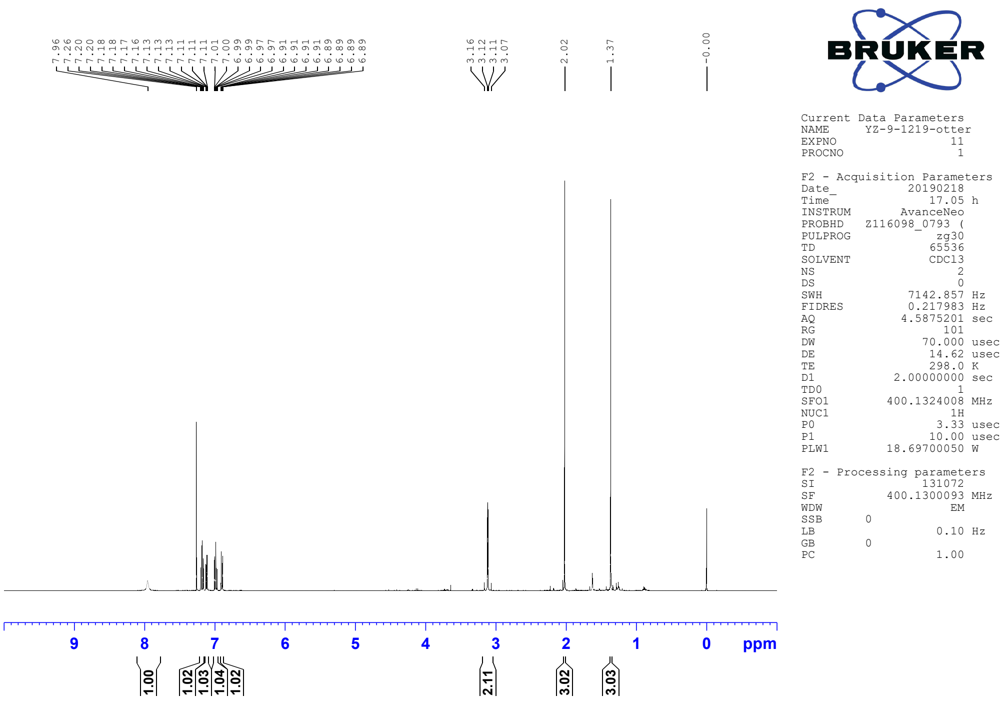

Supplementary Figure S 56. *^{1}H NMR spectrum of* ***14j***.

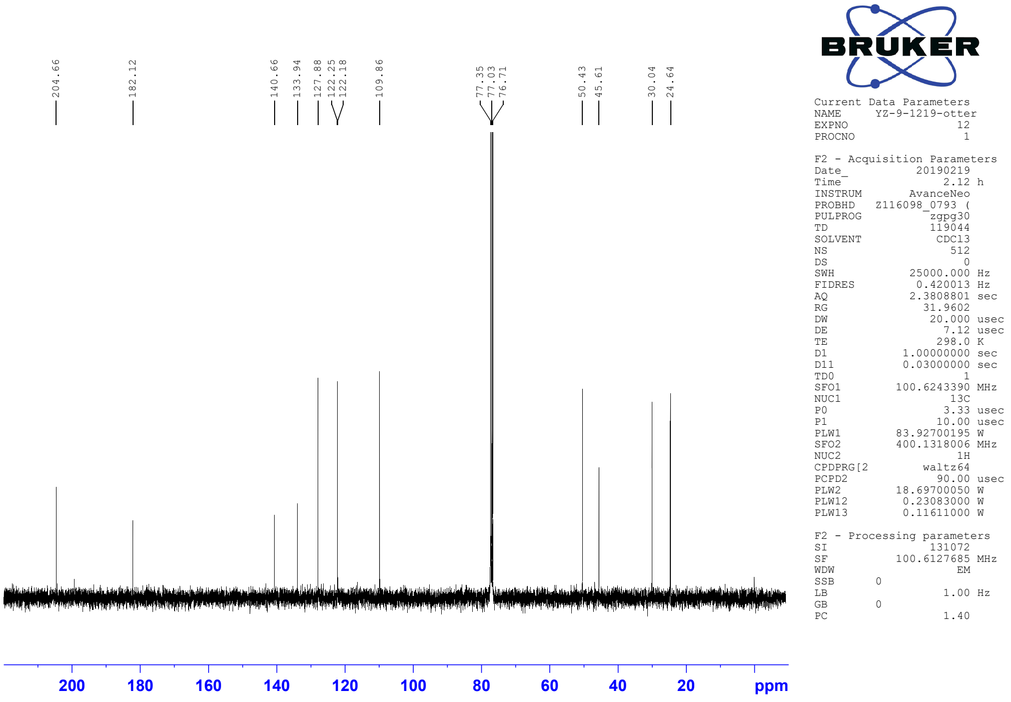

Supplementary Figure S 57. *^{13}C NMR spectrum of* ***14j***.

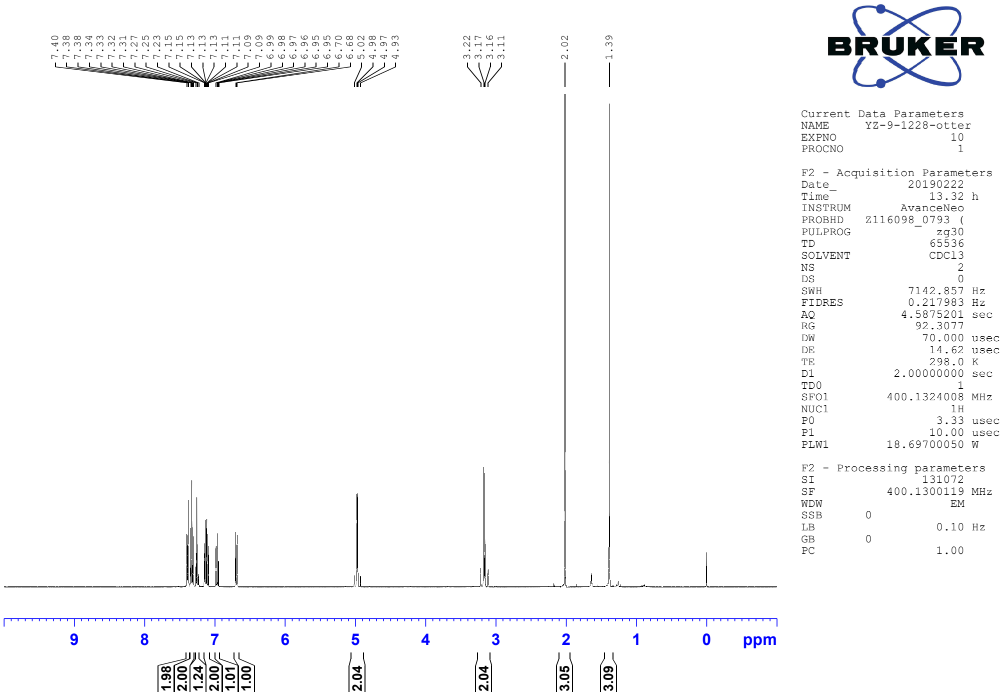

Supplementary Figure S 58. *^{1}H NMR spectrum of* ***14k***.

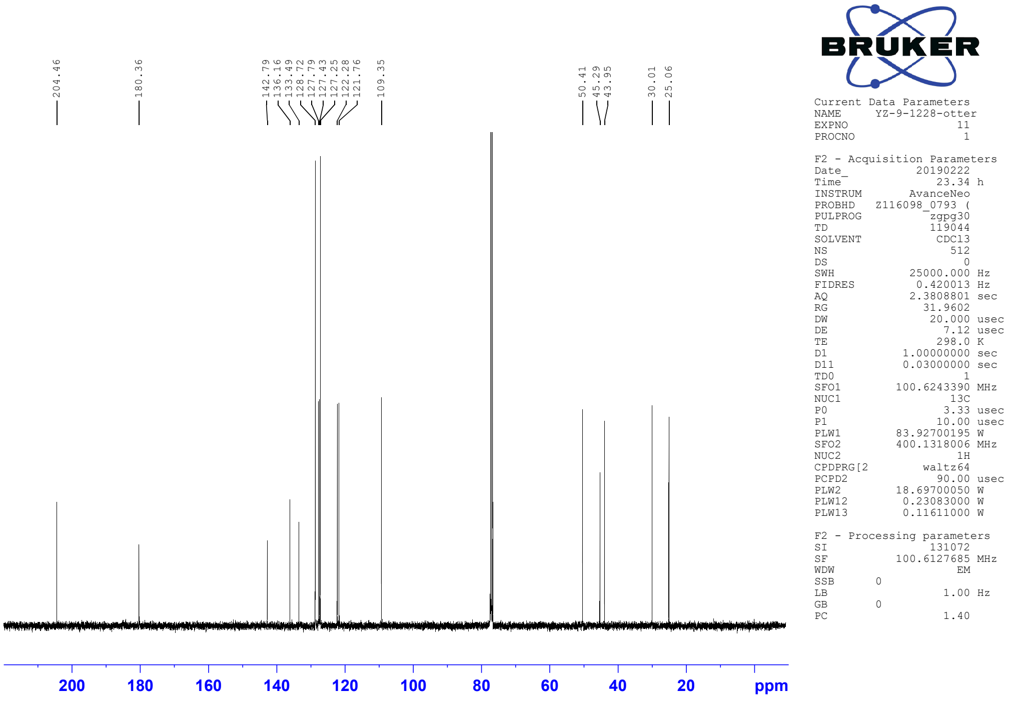

Supplementary Figure S 59. *^{13}C NMR spectrum of* ***14k***.

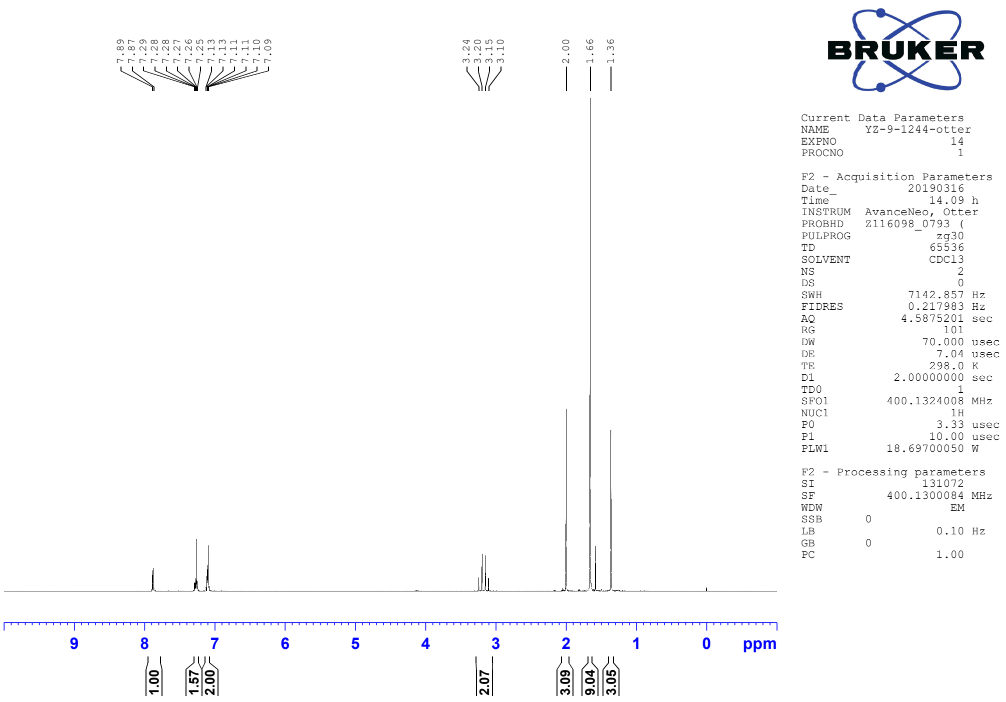

*Supplementary Figure S 60. ¹H NMR spectrum of **14l**.*

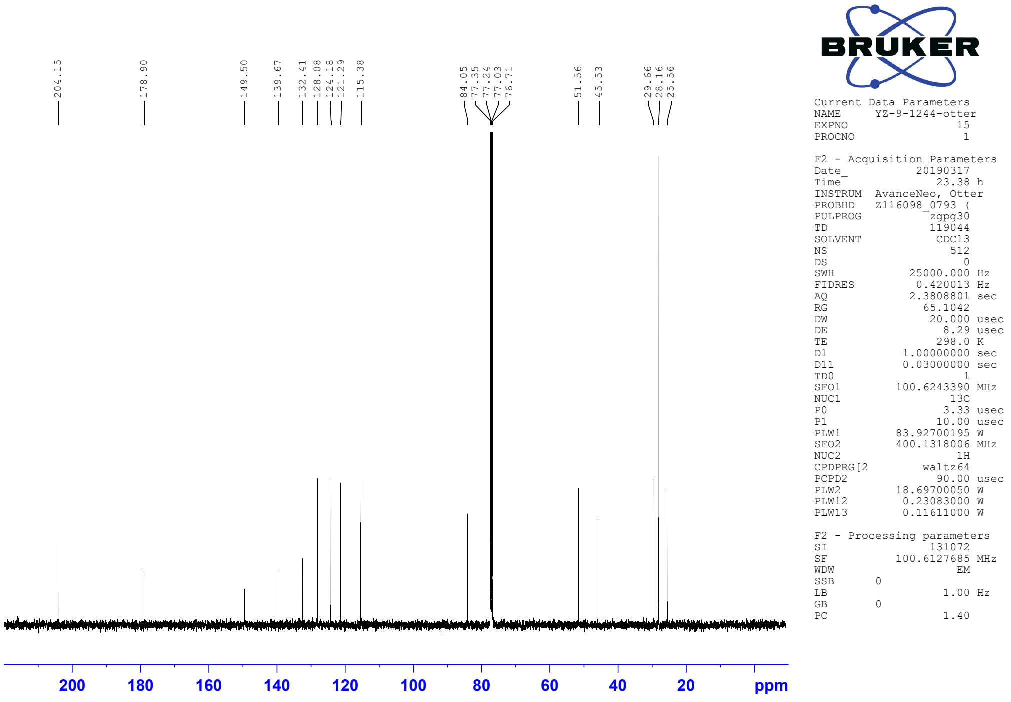

*Supplementary Figure S 61. ¹³C NMR spectrum of **14l**.*

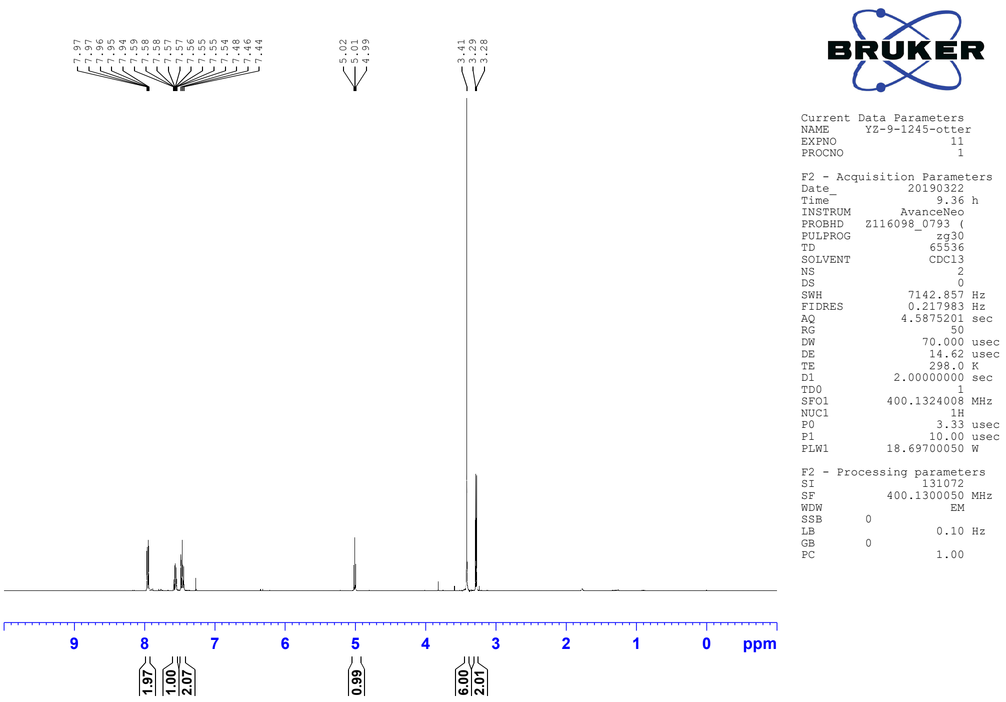

Supplementary Figure S 62. *^{1}H NMR spectrum of* ***14m'***.

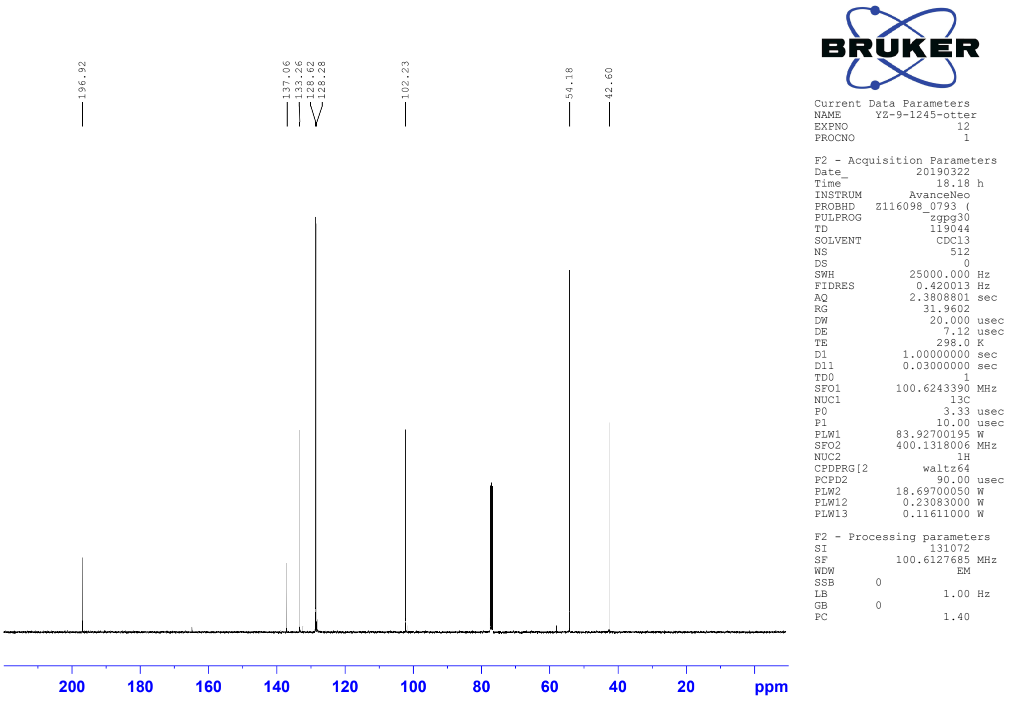

Supplementary Figure S 63. *^{13}C NMR spectrum of* ***14m'***.

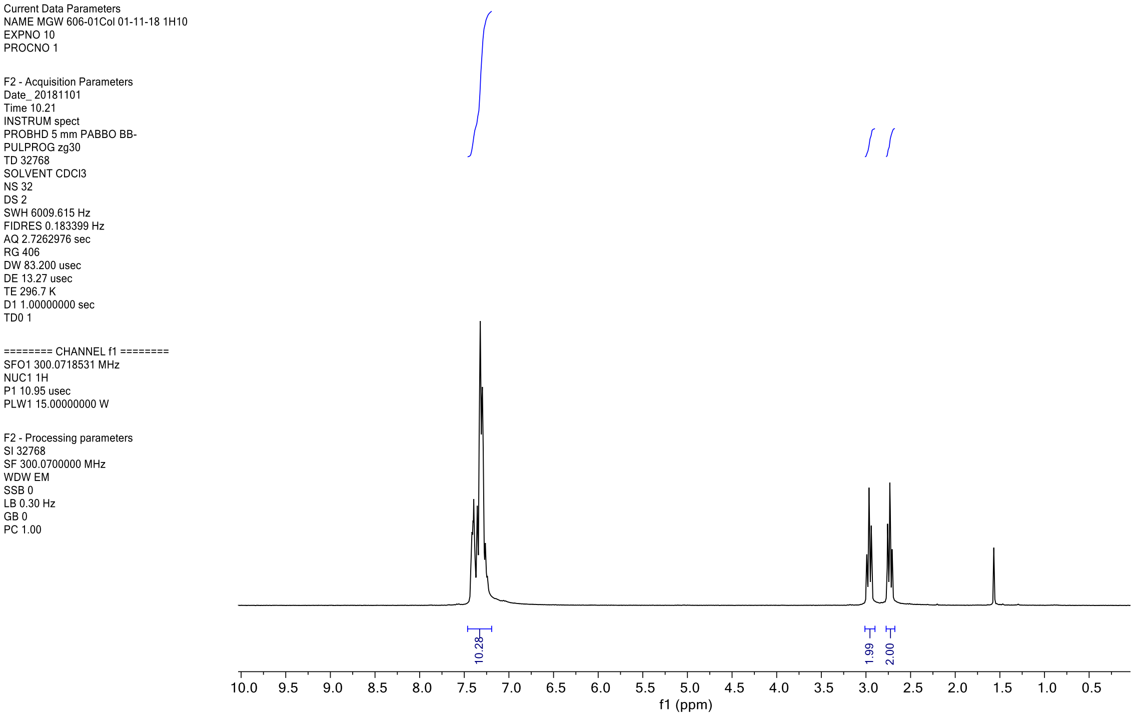

Supplementary Figure S 64. *^{1}H NMR spectrum of* ***15***.

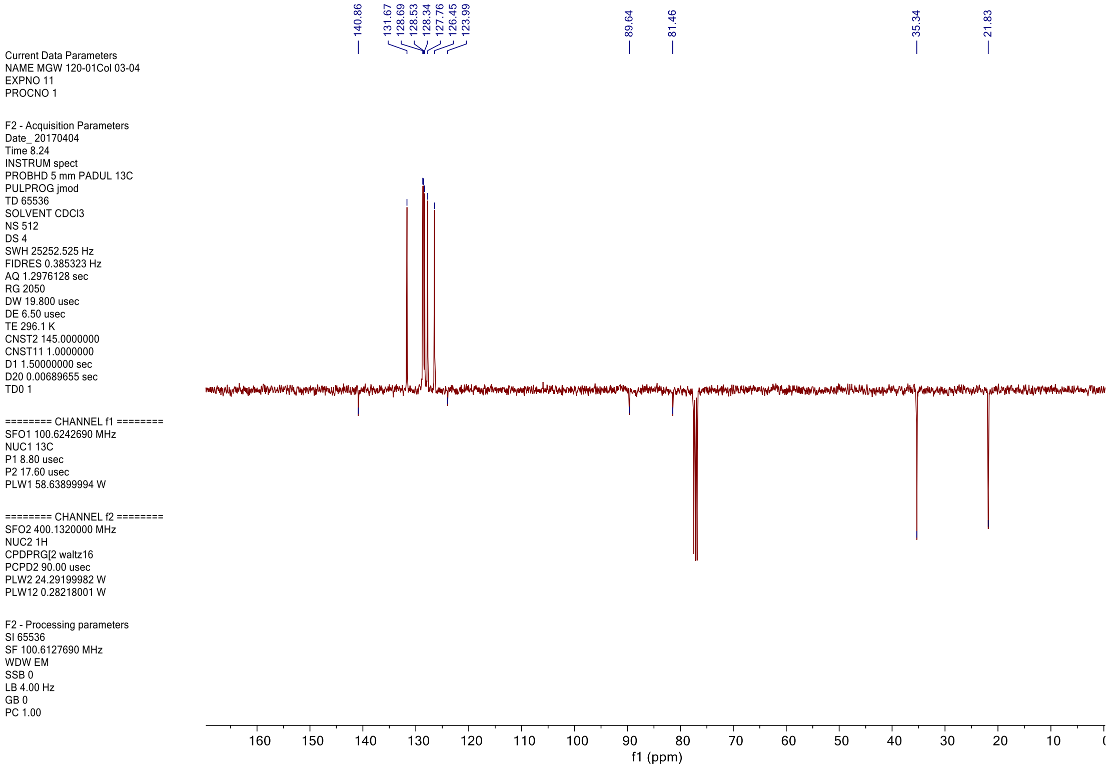

Supplementary Figure S 65. *^{13}C (J-MOD) NMR spectrum of* ***15***.

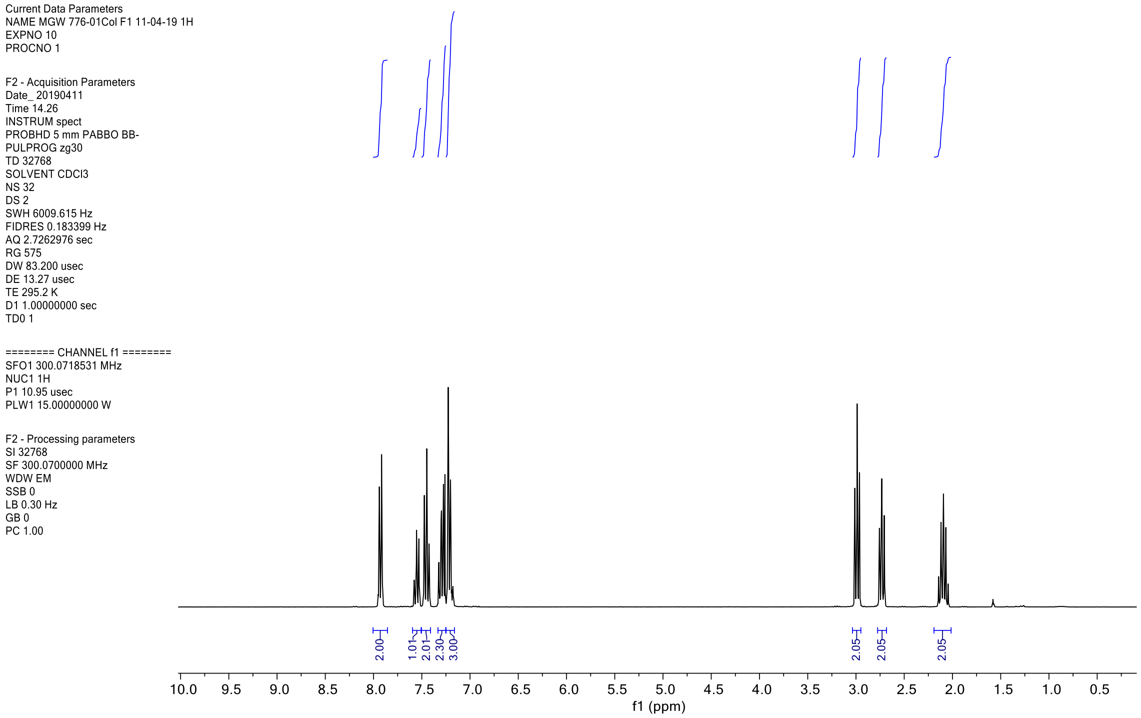

Supplementary Figure S 66. *^{1}H NMR spectrum of* ***16a***.

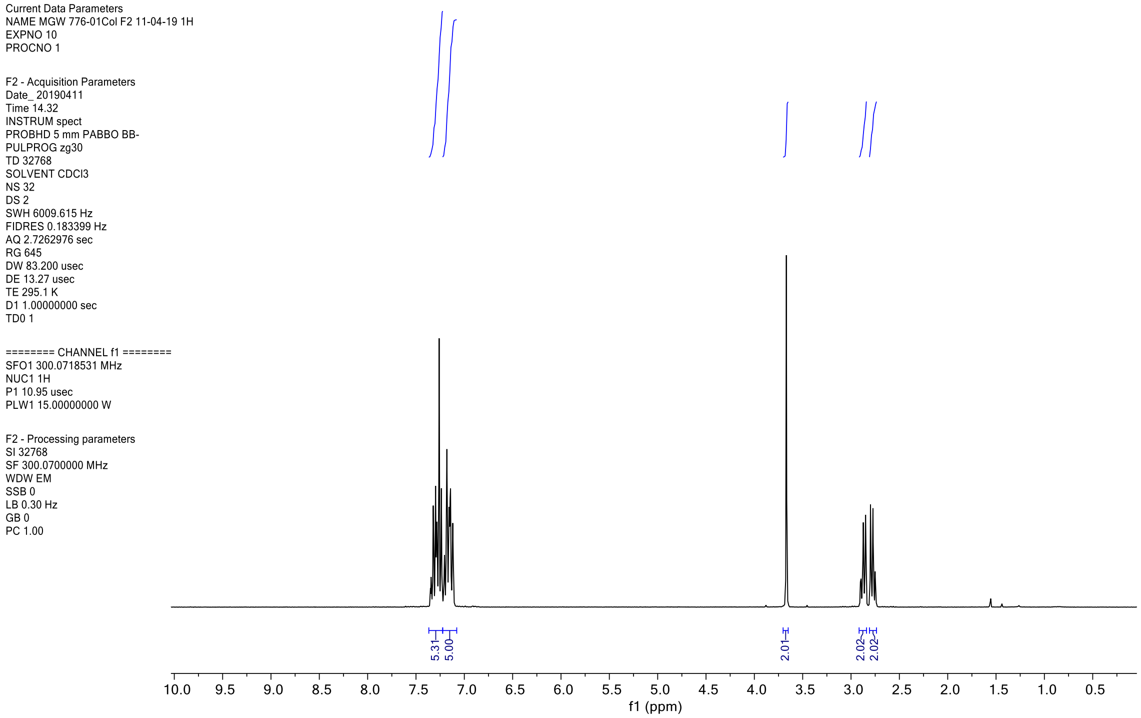

Supplementary Figure S 67. *^{1}H NMR spectrum of* ***16b***.

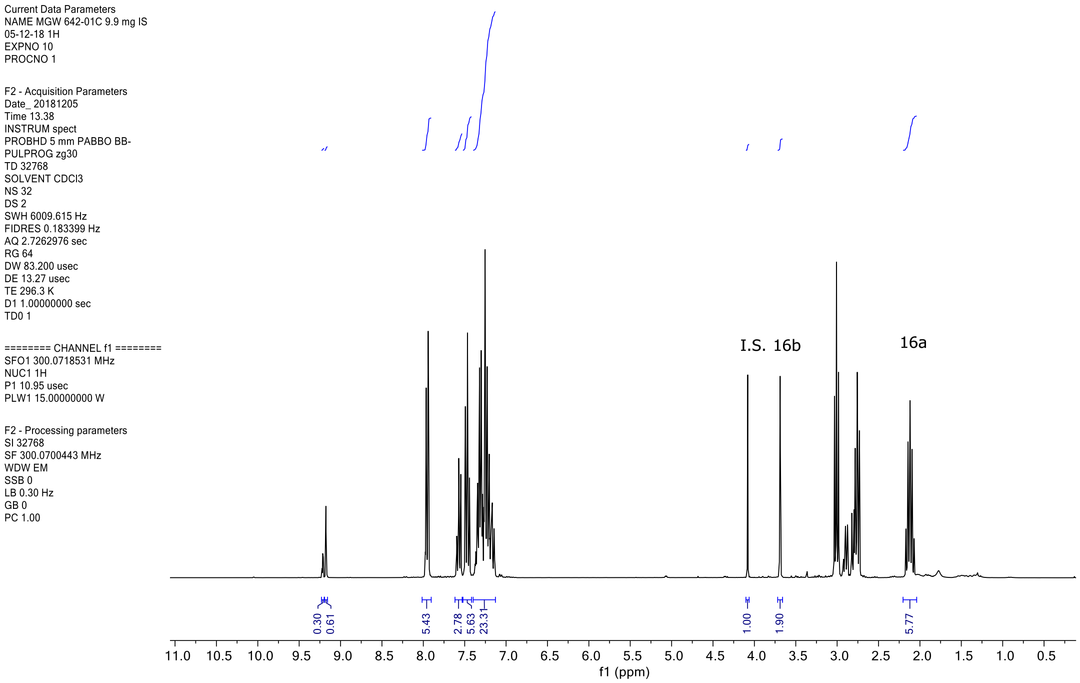

Supplementary Figure S 68. *Representative* [1]*H NMR spectrum of a mixture of **16a** and **16b** resulting obtained directly from a catalysed reaction, includes internal standard (methyl 3,5-dinitrobenzoate)), corresponding to Table 2, entry 1, main text.*

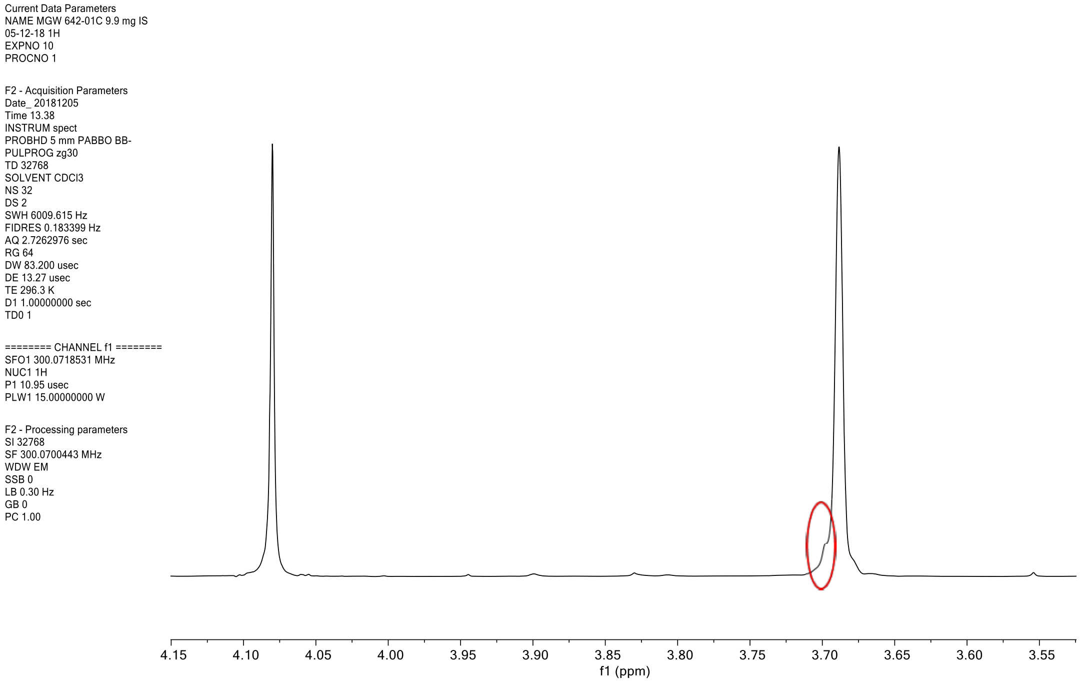

Supplementary Figure S 69. *Overlapping resonance that prevents accurate determination of the proportion of minor regioisomer from NMR spectroscopic analysis (Table 2, entry 1, main text). Signal at 4.08 ppm internal standard 3,5-dinitrobenzoate. Signal at 3.69 benzylic methylene of **16b**.*

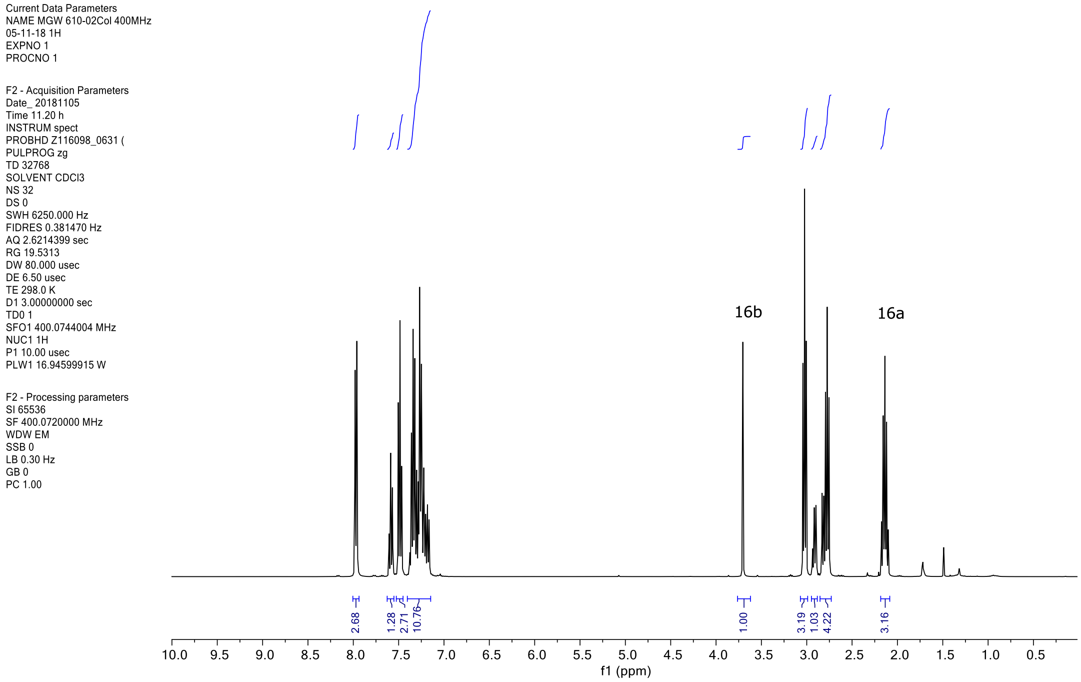

Supplementary Figure S 70. *Representative ^{1}H NMR spectrum of a mixture of* ***16a*** *and* ***16b*** *resulting obtained directly from a catalysed reaction, includes internal standard (methyl 3,5-dinitrobenzoate), corresponding to Table 2, entry 2, main text.*

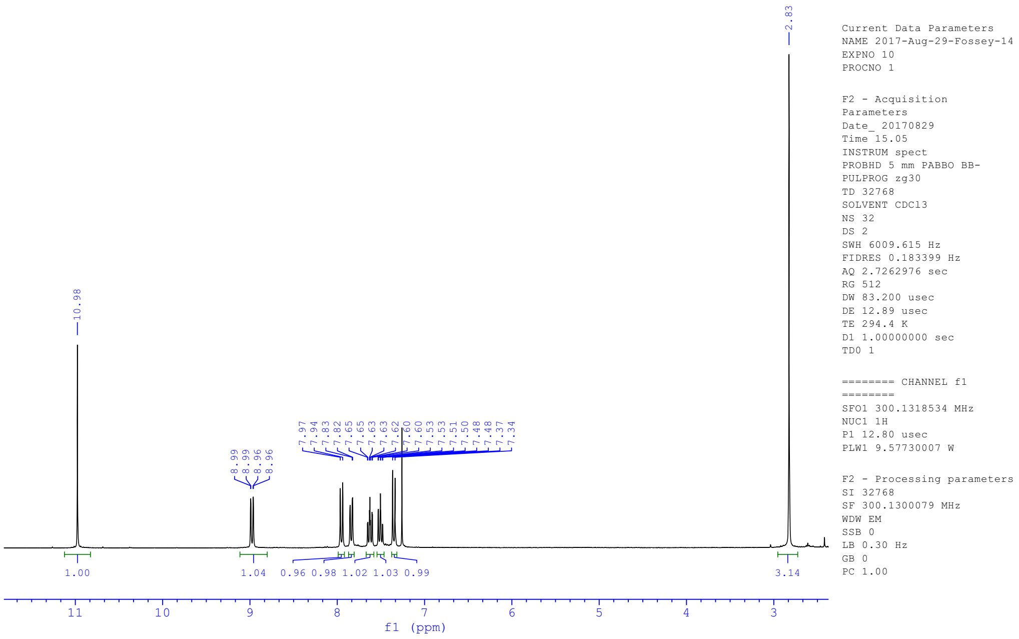

Supplementary Figure S 71. *^{1}H NMR spectrum of* ***S2***.

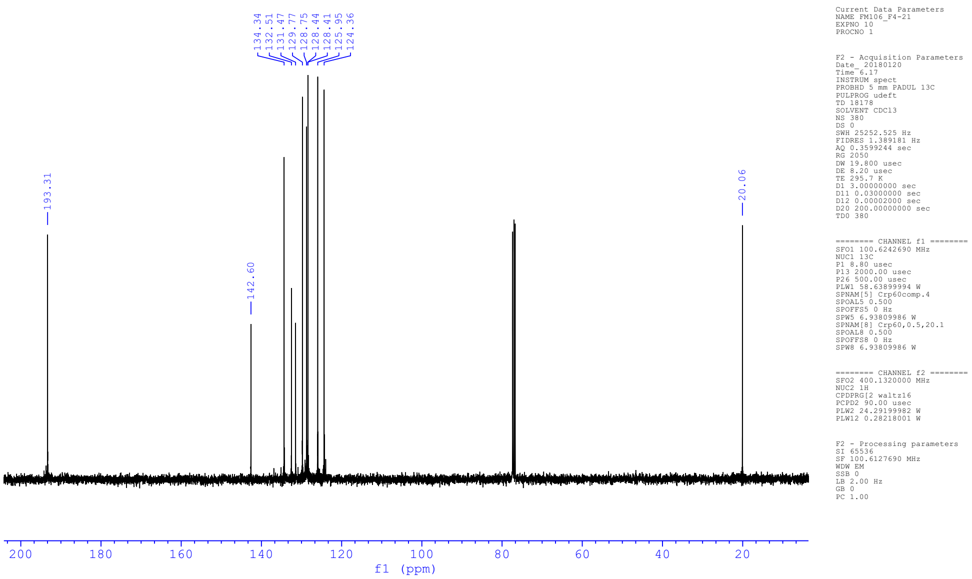

Supplementary Figure S 72. *^{13}C NMR spectrum of* ***S2***.

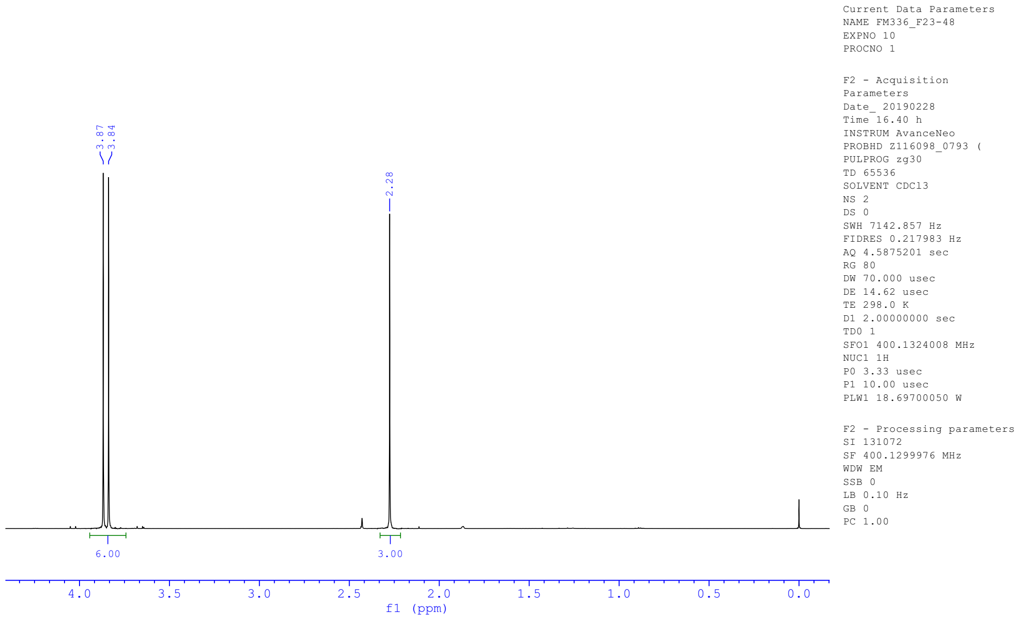

Supplementary Figure S 73. *^{1}H NMR spectrum of* ***S3***.

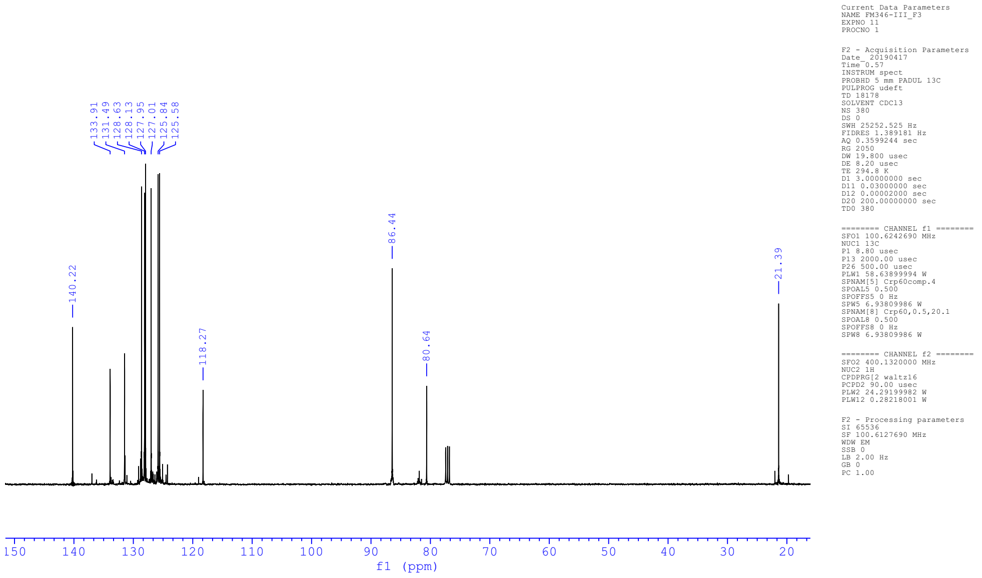

Supplementary Figure S 74. *^{13}C NMR spectrum of* ***S3***.

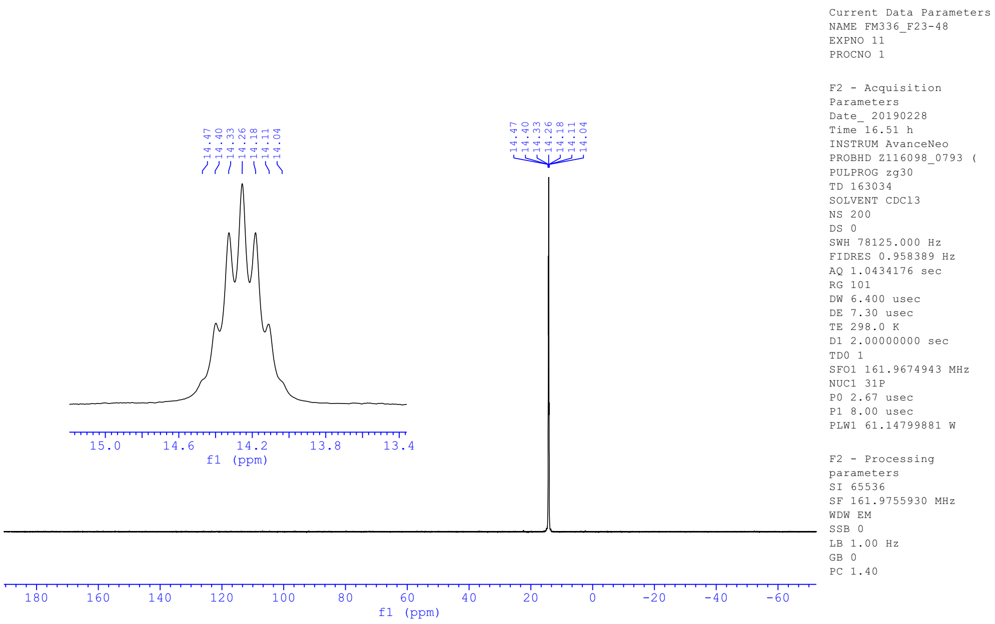

Supplementary Figure S 75. *^{31}P NMR spectrum of **S3**.*

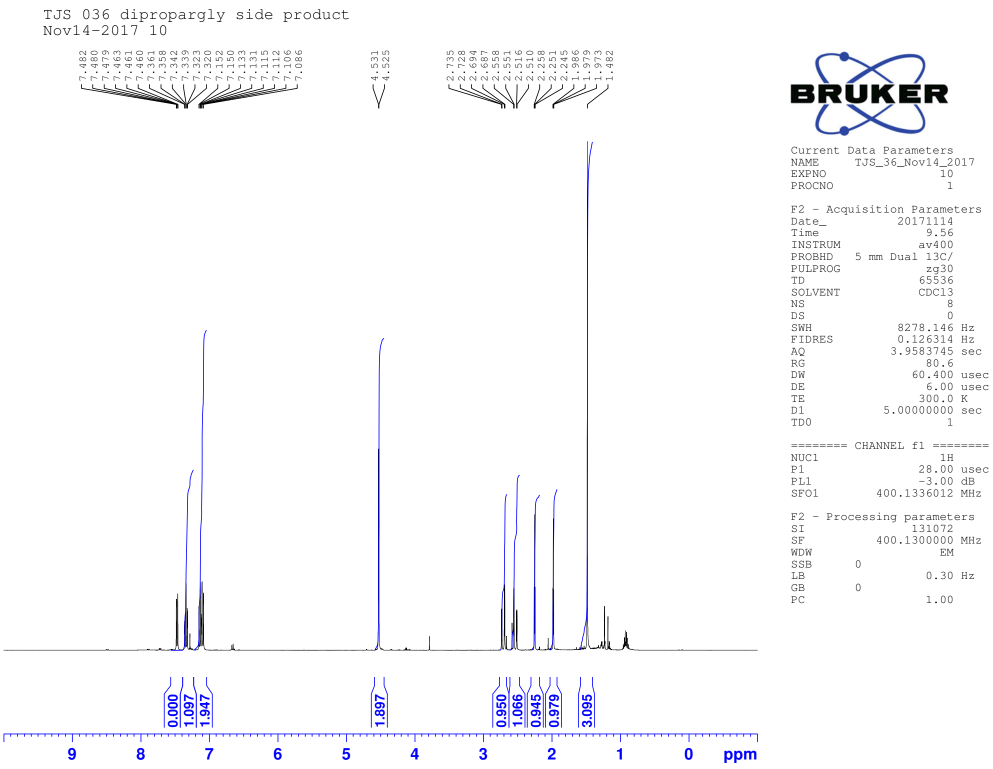

Supplementary Figure S 76. *^{1}H NMR spectrum of **S3**.*

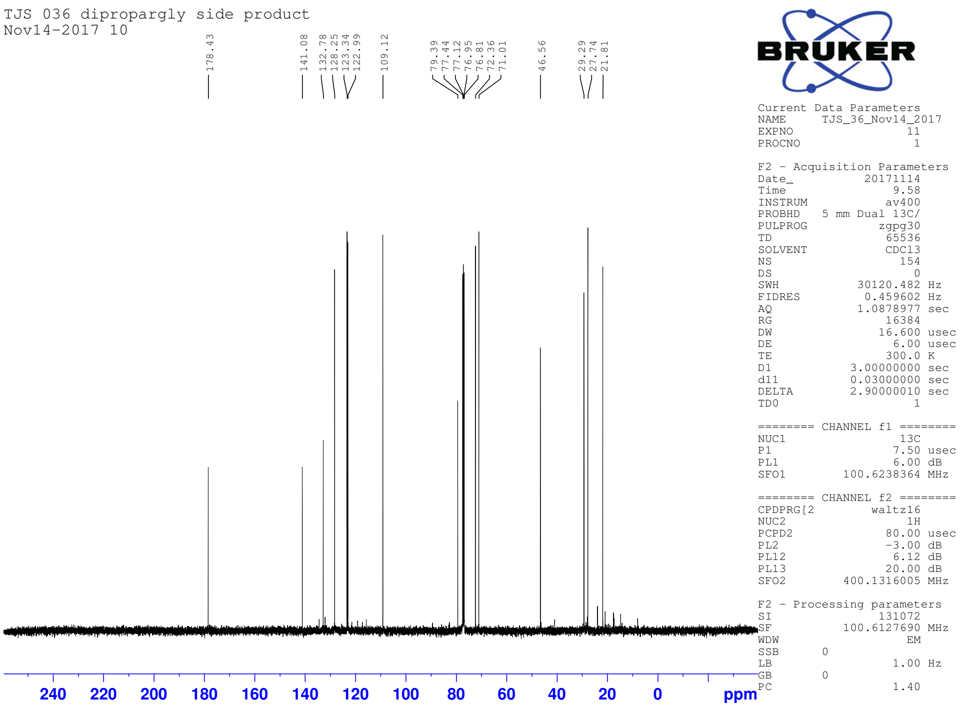

Supplementary Figure S 77. *^{13}C NMR spectrum of **S4**.*

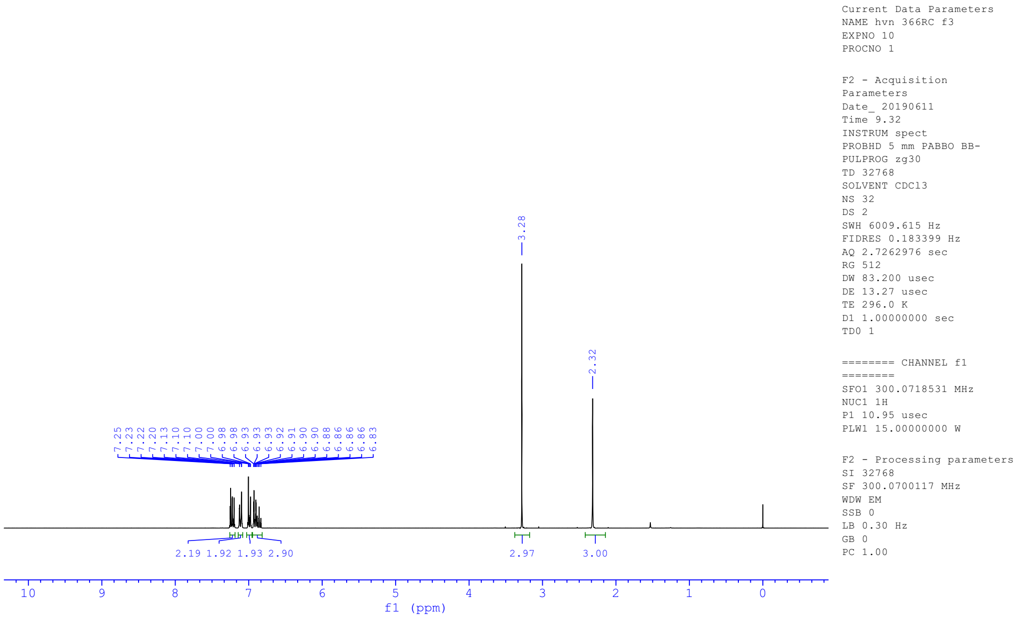

Supplementary Figure S 78. ^{1}H *NMR spectrum of* ***S8a***.

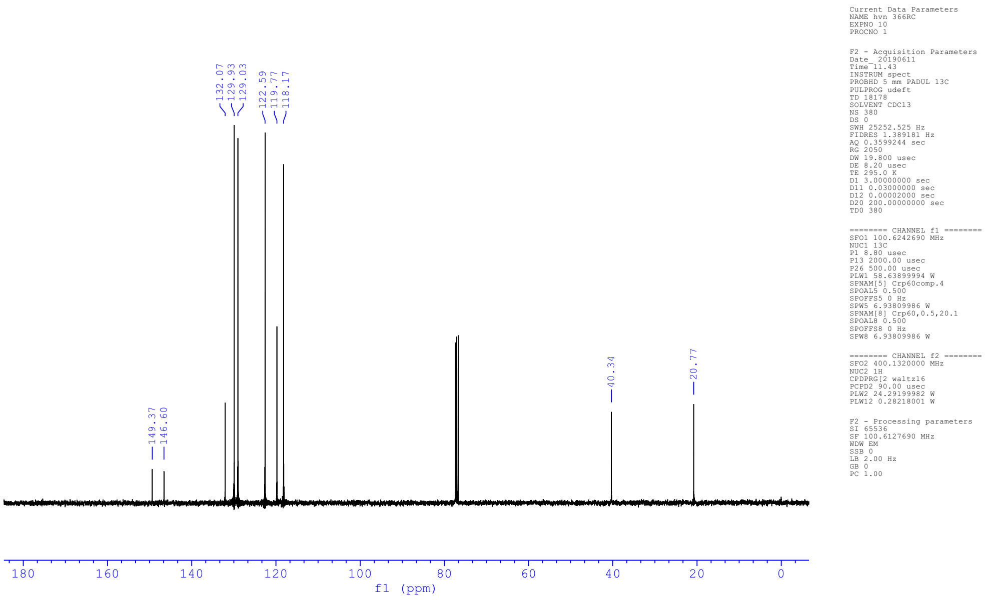

Supplementary Figure S 79. ^{13}C *NMR spectrum of* ***S8a***.

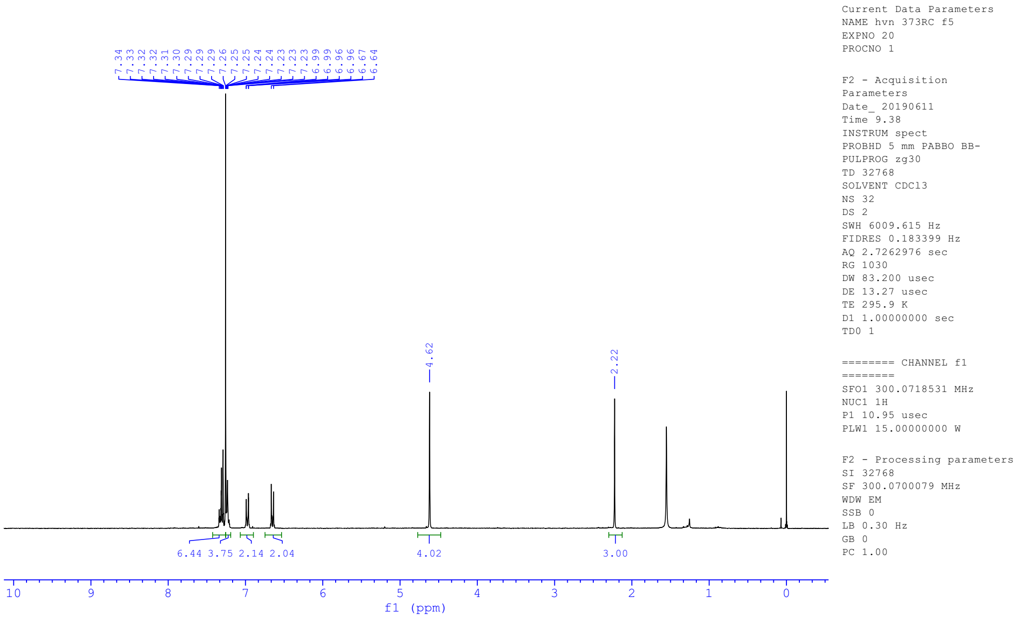

Supplementary Figure S 80. *^{1}H NMR spectrum of **S8b***.

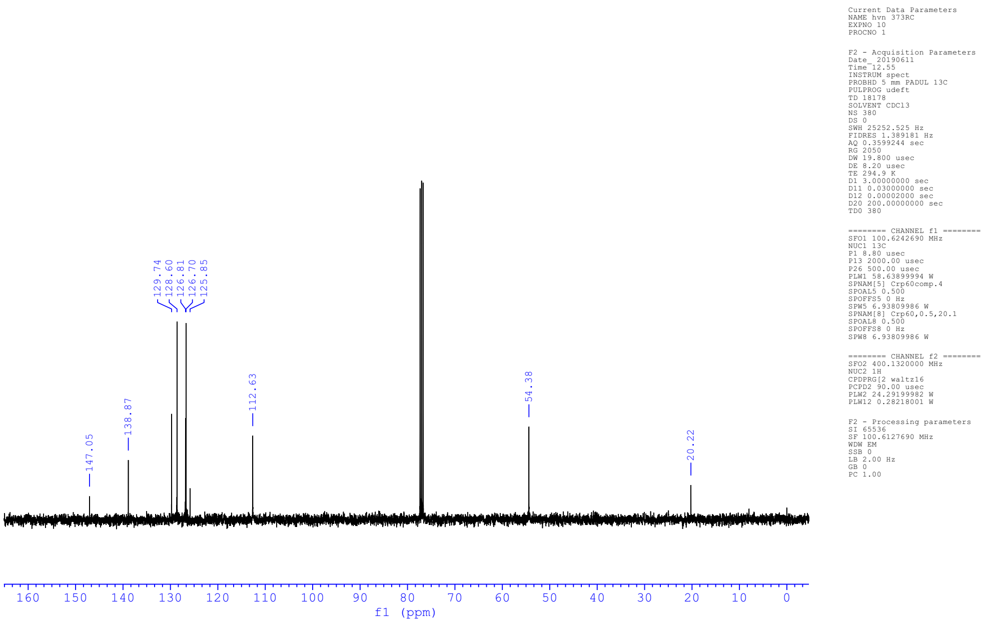

Supplementary Figure S 81. *^{13}C NMR spectrum of **S8b***.

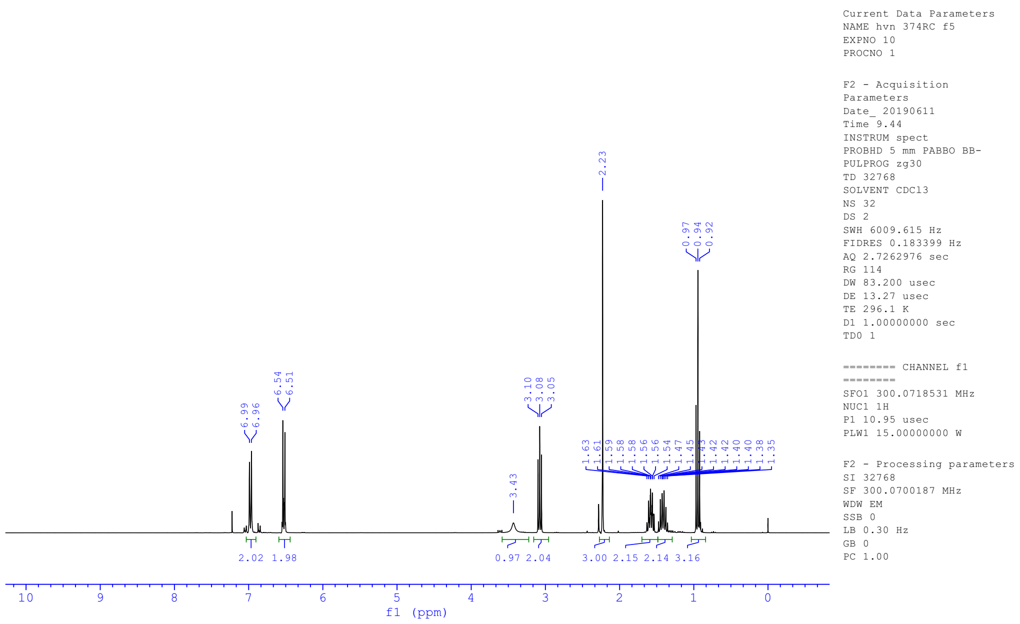

Supplementary Figure S 82. *^{1}H NMR spectrum of* ***S8c***.

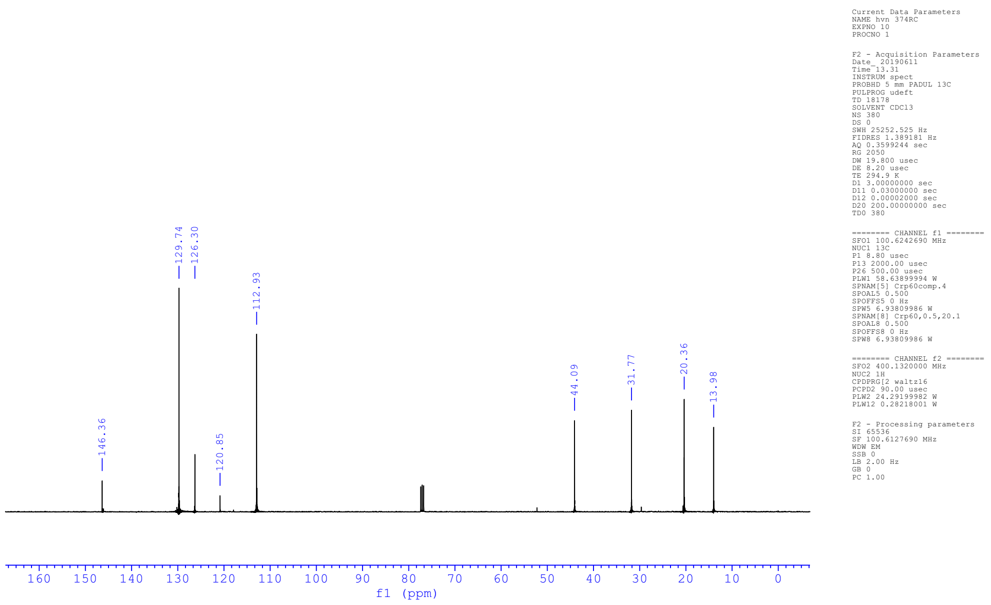

Supplementary Figure S 83. *^{13}C NMR spectrum of* ***S8c***.

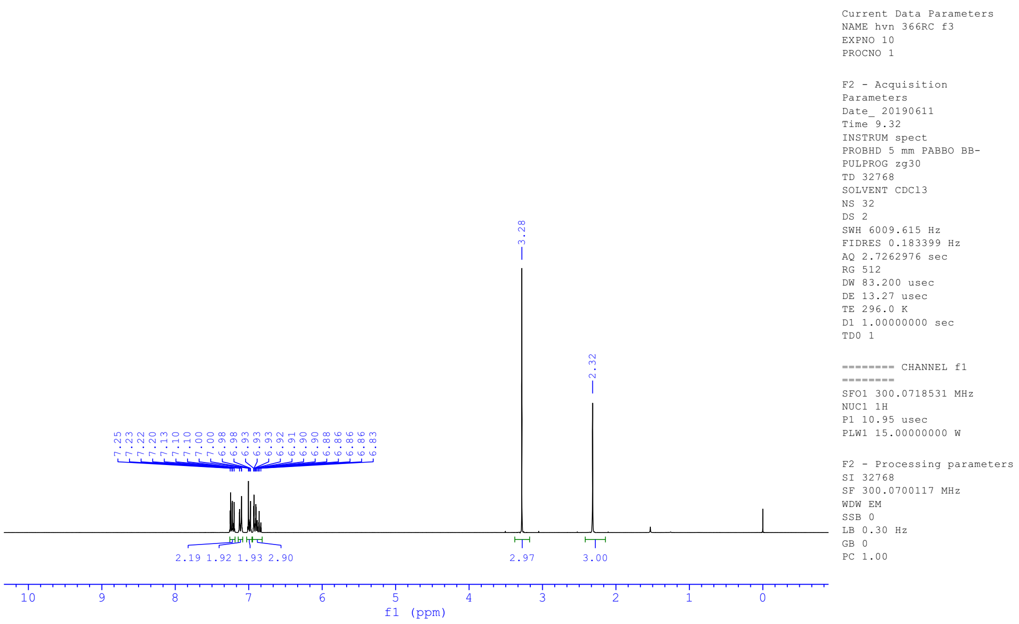

Supplementary Figure S 84. *^{1}H NMR spectrum of* ***S8d***.

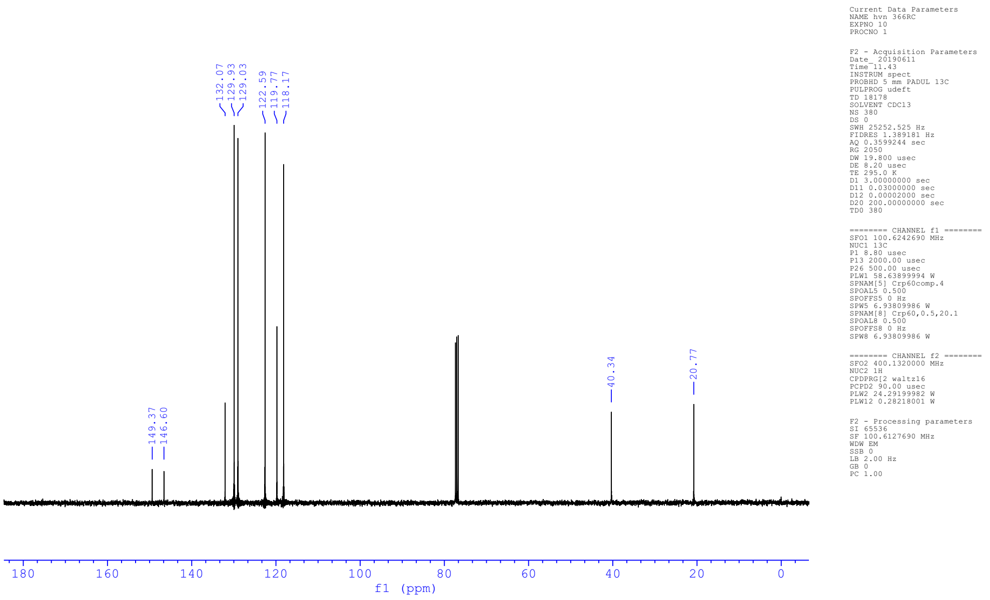

Supplementary Figure S 85. *^{13}C NMR spectrum of* ***S8d***.

X-Ray Crystallographic Information

The datasets were measured on an Agilent SuperNova diffractometer using an Atlas detector. The data collections were driven and processed and absorption corrections were applied using CrysAlisPro.[22] All structure were solved using ShelXT.[23] All structures were refined by a full-matrix least-squares procedure on F^2 in ShelXL.[24] All non-hydrogen atoms were refined with anisotropic displacement parameters. All hydrogen atoms were added at calculated positions and refined by use of a riding model with isotropic displacement parameters based on the equivalent isotropic displacement parameter (U_{eq}) of the parent atom. Reports were produced using OLEX2.[25]

The CIFs for **4a, 4b, 5, 8a, 8b, 9a, 9b, 10, 11, 12** and **S5** have been deposited with the CCDC and have been given the deposition numbers 1922101-1922111 respectively. These numbers contain the supplementary crystallographic data for this paper. These data can be obtained free of charge from The Cambridge Crystallographic Data Centre *via* www.ccdc.cam.ac.uk/data_request/cif.

Compound **4a**

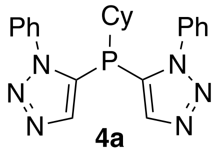

4a

$C_{22}H_{23}N_6P$ (M =402.43 g/mol): monoclinic, space group $P2_1/n$ (no. 14), a = 8.5878(6) Å, b = 30.5588(18) Å, c = 8.7236(7) Å, β = 115.922(9)°, V = 2059.0(3) Å^3, Z = 4, T = 100.01(10) K, μ(CuKα) = 1.343 mm^{-1}, $Dcalc$ = 1.298 g/cm^3, 7580 reflections measured (11.584° ≤ 2Θ ≤ 143.892°), 3925 unique (R_{int} = 0.0346, R_{sigma} = 0.0518) which were used in all calculations. The final R_1 was 0.0467 (I > 2σ(I)) and wR_2 was 0.1327 (all data).

Compound **4b**

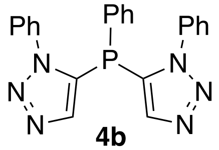

4b

$C_{22}H_{17}N_6P$ (M =396.38 g/mol): triclinic, space group P-1 (no. 2), a = 8.8796(11) Å, b = 10.1744(12) Å, c = 11.6292(13) Å, α = 74.241(10)°, β = 75.180(10)°, γ = 87.296(10)°, V = 977.2(2) Å^3, Z = 2, T = 100.00(10) K, μ(MoKα) = 0.162 mm^{-1}, $Dcalc$ = 1.347 g/cm^3, 7802 reflections measured (6.762° ≤ 2Θ ≤ 52.742°), 3983 unique (R_{int} = 0.0301, R_{sigma} = 0.0495) which were used in all calculations. The final R_1 was 0.0481 (I > 2σ(I)) and wR_2 was 0.1300 (all data).

Compound **5**

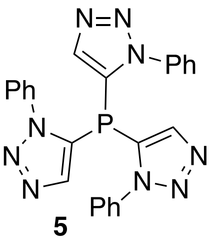

5

$C_{24}H_{18}N_9P$, (CH_3COCH_3) (M =521.52 g/mol): triclinic, space group P-1 (no. 2), a = 10.2901(4) Å, b = 10.5099(5) Å, c = 13.7597(7) Å, α = 101.908(4)°, β = 110.581(4)°, γ = 93.969(4)°, V = 1346.71(11) Å^3, Z = 2, T = 100.01(10) K, μ(MoKα) = 0.140 mm^{-1}, Dcalc = 1.286 g/cm^3, 10967 reflections measured (5.394° ≤ 2Θ ≤ 59.17°), 6298 unique (R_{int} = 0.0248, R_{sigma} = 0.0448) which were used in all calculations. The final R_1 was 0.0454 (I > 2σ(I)) and wR_2 was 0.1080 (all data).

Compound **8a**

$C_{20}H_{28}N_3PClAu$ (M =573.84 g/mol): triclinic, space group P-1 (no. 2), a = 9.3253(3) Å, b = 10.7200(5) Å, c = 11.7307(7) Å, α = 108.817(5)°, β = 98.520(4)°, γ = 103.935(4)°, V = 1044.28(9) $Å^3$, Z = 2, T = 100.01(10) K, μ(MoKα) = 7.257 mm^{-1}, $Dcalc$ = 1.825 g/cm^3, 9349 reflections measured (6.726° ≤ 2Θ ≤ 58.854°), 4915 unique (R_{int} = 0.0334, R_{sigma} = 0.0513) which were used in all calculations. The final R_1 was 0.0277 (I > 2σ(I)) and wR_2 was 0.0538 (all data).

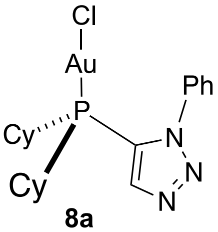

8a

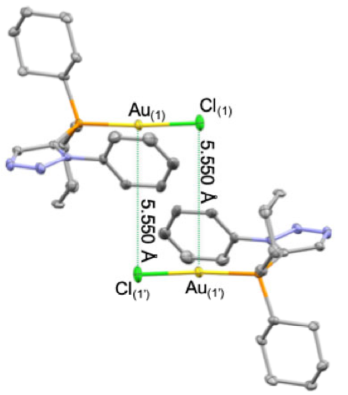

Supplementary Figure S 86. *Phenyl centroid metal distances in **8a** 3.502 Å. Shortest intermolecular Au…Cl distance 5.550 Å*

Compound **8b**

$C_{20}H_{16}AuClN_3P$ (M =561.74 g/mol): triclinic, space group P-1 (no. 2), a = 9.1495(3) Å, b = 13.5665(5) Å, c = 15.9837(6) Å, α = 77.761(3)°, β = 82.352(3)°, γ = 79.954(3)°, V = 1899.64(12) $Å^3$, Z = 4, T = 100.01(10) K, μ(MoKα) = 7.978 mm^{-1}, $Dcalc$ = 1.964 g/cm^3, 19415 reflections measured (5.242° ≤ 2Θ ≤ 58.738°), 9018 unique (R_{int} = 0.0251, R_{sigma} = 0.0395) which were used in all calculations. The final R_1 was 0.0342 (I > 2σ(I)) and wR_2 was 0.0621 (all data).

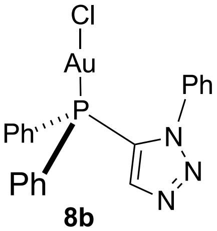

8b

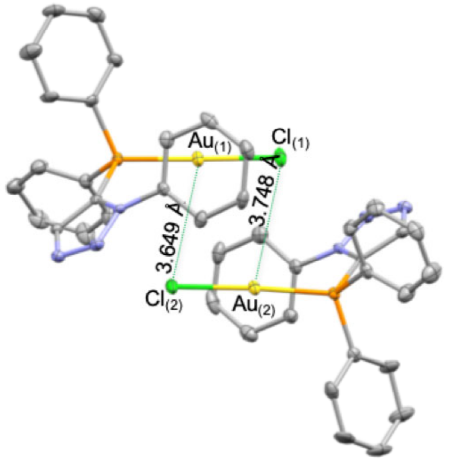

Supplementary Figure S 87. *Centroid metal distances of the two molecules **8b** within the unit cell 3.664 Å and 3.365 Å. Shortest intermolecular Au…Cl distance 3.649 Å.*

Compound **9a**

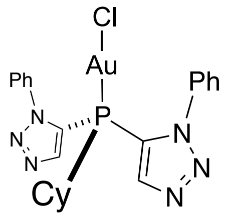

9a

$C_{22}H_{23}AuClN_6P$ (*M* =634.85 g/mol): monoclinic, space group P2$_1$/n (no. 14), *a* = 15.1740(13) Å, *b* = 9.0970(5) Å, *c* = 17.9495(17) Å, *β* = 114.837(11)°, *V* = 2248.5(4) Å^3, *Z* = 4, *T* = 100.01(10) K, μ(CuKα) = 14.241 mm^{-1}, *Dcalc* = 1.875 g/cm^3, 25510 reflections measured (10.002° ≤ 2Θ ≤ 136.466°), 4116 unique (R_{int} = 0.0965, R_{sigma} = 0.0400) which were used in all calculations. The final R_1 was 0.0366 (I > 2σ(I)) and wR_2 was 0.1032 (all data).

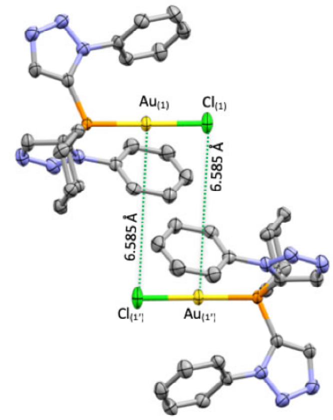

Supplementary Figure S 88*. Phenyl centroid metal distances in* ***9a*** *3.513 Å and 3.517 Å. Shortest intermolecular Au…Cl distance 6.585 Å.*

Compound **9b**

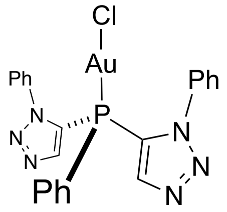

9b

$C_{22}H_{17}N_6PClAu$ (M =628.80 g/mol): monoclinic, space group P2$_1$/c (no. 14), a = 8.2902(2) Å, b = 14.1064(4) Å, c = 19.1489(6) Å, β = 99.717(3)°, V = 2207.24(11) Å^3, Z = 4, T = 100.01(10) K, μ(CuKα) = 14.506 mm^{-1}, Dcalc = 1.892 g/cm^3, 8034 reflections measured (7.824° ≤ 2Θ ≤ 143.504°), 4217 unique (R_{int}= 0.0314, R_{sigma} = 0.0405) which were used in all calculations. The final R_1 was 0.0277 (I > 2σ(I)) and wR_2 was 0.0719 (all data).

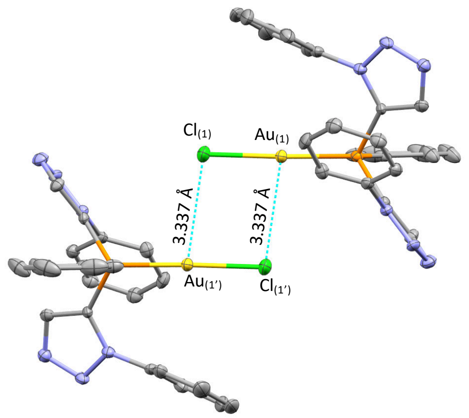

Supplementary Figure S 89*. Phenyl centroid metal distances in* ***9b*** *3.662 Å and 4.675. Shortest intermolecular Au…Cl distance 3.337 Å.*

Compound **10**

$C_{24}H_{18}AuClN_9P[CH_2Cl_2]$ (*M* =780.79 g/mol): triclinic, space group P-1 (no. 2), *a* = 10.2434(4) Å, *b* = 14.6032(6) Å, *c* = 19.0803(7) Å, *α* = 92.986(3)°, *β* = 93.407(3)°, *γ* = 100.172(3)°, *V* = 2798.63(19) Å^3, *Z* = 4, *T* = 100.00(10) K,

μ(MoKα) = 5.634 mm^{-1}, *Dcalc* = 1.853 g/cm^3, 28543 reflections measured (4.508° ≤ 2Θ ≤ 59.02°), 13281 unique (R_{int} = 0.0252, R_{sigma} = 0.0392) which were used in all calculations. The final R_1 was 0.0253 (I > 2 σ (I)) and wR2 was 0.0544 (all data).

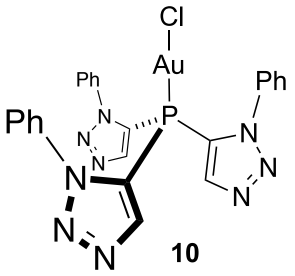

10

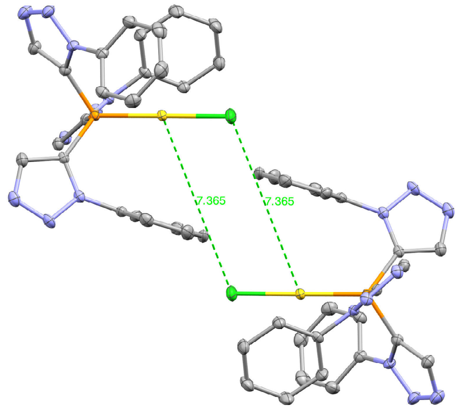

Supplementary Figure S 90*. Centroid metal distances of the two molecules* ***10*** *within the unit cell: (3.456 Å, 3.581 Å and 3.634 Å) and (3.351 Å, 3.692 Å and 3.759 Å). Shortest intermolecular Au…Cl distance 7.365 Å.*

Compound **11**

$C_{22}H_{32}AuClN_3O_2P[CHCl_3]$ (*M* =753.26 g/mol): monoclinic, space group P2₁/c (no. 14), *a* = 16.3596(7) Å, *b* = 19.1615(7) Å, *c* = 9.1916(4) Å, *β* = 94.769(4)°, *V* = 2871.4(2) $Å^3$, *Z* = 4, *T* = 100.01(10) K, μ(CuKα) = 13.785 mm^{-1}, *Dcalc* = 1.742 g/cm^3, 10995 reflections measured (7.12° ≤ 2Θ ≤ 140.136°), 5404 unique (R_{int} = 0.0587, R_{sigma} = 0.0713) which were used in all calculations. The final R_1 was 0.0527 (I > 2σ(I)) and wR_2 was 0.1441 (all data).

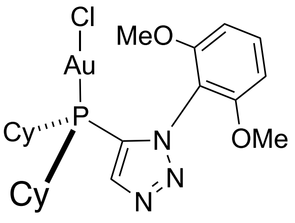

11

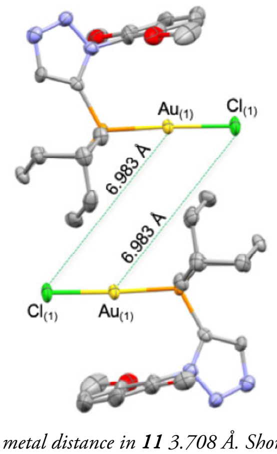

Supplementary Figure S 91*. Phenyl centroid metal distance in* ***11*** *3.708 Å. Shortest intermolecular Au…Cl distance 6.983 Å*

Compound **12**

$C_{44}H_{40}AuClN_6O_4P_2$, $3(CH_2Cl_2)$ (M =1265.95 g/mol): monoclinic, space group $P2_1/c$ (no. 14), a = 11.7669(5) Å, b = 41.4044(10) Å, c = 11.5627(4) Å, β = 115.166(5)°, V = 5098.6(4) Å^3, Z = 4, T = 100.01(10) K, μ(MoKα) = 3.364 mm^{-1}, $Dcalc$ = 1.649 g/cm^3, 28129 reflections measured (4.362° ≤ 2Θ ≤ 52.742°), 10437 unique (R_{int} = 0.0360, R_{sigma} = 0.0493) which were used in all calculations. The final R_1 was 0.0320 (I > 2σ(I)) and wR_2 was 0.0575 (all data).

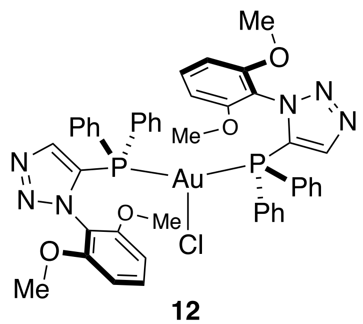

12

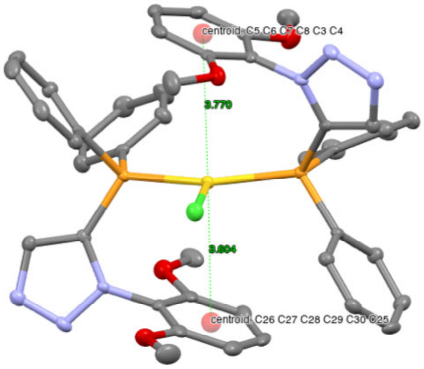

Supplementary Figure S 92. *Centroid metal distances of the two molecules of* ***12*** *within the unit cell, 3.770 Å and 3.604 Å.*

Compound **S5** (S-Phos Gold(I) chloride complex)

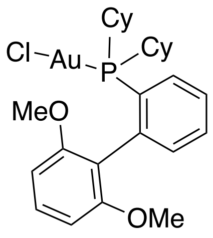

S5

$C_{52}H_{70}Au_2Cl_2O_4P_2$, (CH_2Cl_2) (M =1370.77 g/mol): triclinic, space group P-1 (no. 2), a = 10.0987(3) Å, b = 12.4469(4) Å, c = 22.7136(7) Å, α = 96.723(3)°, β = 99.291(2)°, γ = 107.669(2)°, V = 2642.47(15) Å^3, Z = 2, T = 100.01(10) K, μ(MoKα) = 5.852 mm^{-1}, $Dcalc$ = 1.723 g/cm^3, 27290 reflections measured (5.044° ≤ 2Θ ≤ 52.744°), 10815 unique (R_{int} = 0.0259, R_{sigma} = 0.0358) which were used in all calculations. The final R_1 was 0.0224 (I > 2σ(I)) and wR_2 was 0.0418 (all data). This structure has been reported previously by Rabaa *et al.* from a twinned dataset measured at 200 K.[26] The structure presented here is refined from a dataset measured at 100 K from a single crystal.

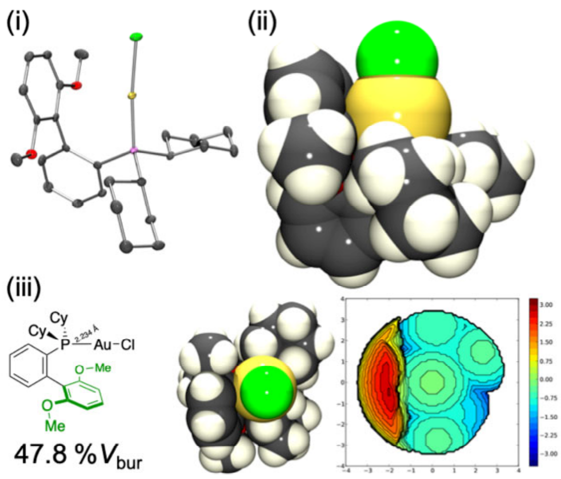

Supplementary Figure S 93.

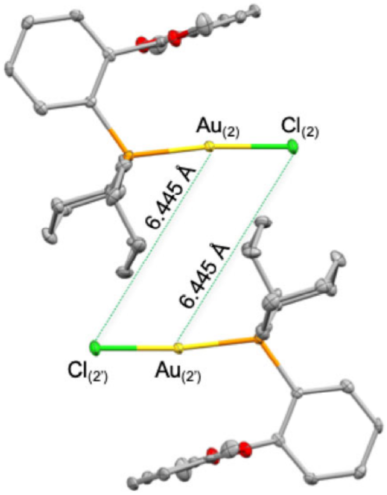

Supplementary Figure S 94. *Centroid metal distances of the two molecules of* ***S5*** *within the unit cell, 3.365 Å and 3.429. Shortest intermolecular Au…Cl distance 6.445 Å.*

Supplementary References

[1] U. Eggenberger, G. Bodenhausen, *Angew. Chem. Int. Ed.* **1990**, *29*, 374-383.

[2] M. Piotto, M. Bourdonneau, K. Elbayed, J. M. Wieruszeski, G. Lippens, *Magn. Reson. Chem.* **2006**, *44*, 943-947.

[3] J. A. Parkinson, *eMagRes* **2015**, *4*, 69-82.

[4] L. Landucci Lawrence, *Holzforschung* **1991**, *45*, 425.

[5] For example: Compound **29b** where 'app br s' refers to signal that appears as a broad singlet, over a given range, but arises due to overlap of three signal of similar chemical shift within that range.

[6] H. E. Gottlieb, V. Kotlyar, A. Nudelman, *J. Org. Chem.* **1997**, *62*, 7512-7515.

[7] Y. Zhao, H. van Nguyen, L. Male, P. Craven, B. R. Buckley, J. S. Fossey, *Organometallics* **2018**, *37*, 4224-4241.

[8] H. Gilman, F. K. Cartledge, *J. Organomet. Chem.* **1964**, *2*, 447-454.

[9] X. Cong, H. Tang, X. Zeng, *J. Am. Chem. Soc.* **2015**, *137*, 14367-14372.

[10] E. Quesada, S. A. Raw, M. Reid, E. Roman, R. J. K. Taylor, *Tetrahedron* **2006**, *62*, 6673-6680.

[11] Y. Zhou, F. Ye, Q. Zhou, Y. Zhang, J. Wang, *Org. Lett.* **2016**, *18*, 2024-2027.

[12] N. Kadoya, M. Murai, M. Ishiguro, J. i. Uenishi, M. Uemura, *Tetrahedron Lett.* **2013**, *54*, 512-514.

[13] C. Nájera, J. Sansano, A. Ortega-Martínez, C. Molina, C. Moreno-Cabrerizo, *Synthesis* **2017**, *49*, 5203-5210.

[14] D. A. Chaudhari, R. A. Fernandes, *J. Org. Chem.* **2016**, *81*, 2113-2121.

[15] S. A. Van Arman, A. J. Zimmet, I. E. Murray, *J. Org. Chem.* **2016**, *81*, 3528-3532.

[16] S. Ma, L. Wang, *J. Org. Chem.* **1998**, *63*, 3497-3498.

[17] R. E. Ebule, D. Malhotra, G. B. Hammond, B. Xu, *Adv. Synth. Catal.* **2016**, *358*, 1478-1481.

[18] J.-C. Hsieh, Y.-C. Chen, A.-Y. Cheng, H.-C. Tseng, *Org. Lett.* **2012**, *14*, 1282-1285.

[19] C. Zhao, X. Jia, X. Wang, H. Gong, *J. Am. Chem. Soc.* **2014**, *136*, 17645-17651.

[20] Y. Zhao, H. Van Nguyen, L. Male, P. Craven, B. R. Buckley, J. S. Fossey, *ChemRxiv* **2018**, *http://doi.org/10.26434/chemrxiv.6823259*

[21] Q. Dai, W. Gao, D. Liu, L. M. Kapes, X. Zhang, *J. Org. Chem.* **2006**, *71*, 3928-3934.

[22] CrysAlisPro, **2013**, *Version 1.171.36.28*, Agilent Technologies.

[23] G. M. Sheldrick, *Acta Crystallogr. Sect. A: Found. Crystallogr.* **2015**, *A71*, 3-8.

[24] G. M. Sheldrick, *Acta Crystallogr. Sect. C* **2015**, *C71*, 3-8.

[25] O. V. Dolomanov, L. J. Bourhis, R. J. Gildea, J. A. K. Howard, H. Puschmann, *J. Appl. Crystallogr.* **2009**, *42*, 339-341.

[26] M. Touil, B. Bechem, A. S. K. Hashmi, B. Engels, M. A. Omary, H. Rabaâ, *J. Mol. Struct-Theochem.* **2010**, *957*, 21-25.
